# Genome-based classification of micromonosporae with a focus on their biotechnological and ecological potential

**DOI:** 10.1038/s41598-017-17392-0

**Published:** 2018-01-11

**Authors:** Lorena Carro, Imen Nouioui, Vartul Sangal, Jan P. Meier-Kolthoff, Martha E. Trujillo, Maria del Carmen Montero-Calasanz, Nevzat Sahin, Darren Lee Smith, Kristi E. Kim, Paul Peluso, Shweta Deshpande, Tanja Woyke, Nicole Shapiro, Nikos C. Kyrpides, Hans-Peter Klenk, Markus Göker, Michael Goodfellow

**Affiliations:** 1 School of Biology, Newcastle University, Newcastle upon Tyne, UK; 2 Department of Biomedical Sciences, Northumbria University, Newcastle upon Tyne, UK; 3 Leibniz Institute DSMZ–German Collection of Microorganisms and Cell Cultures, Inhoffenstraße 7B, Braunschweig, Germany; 4 Departamento de Microbiologia y Genetica, Lab 214, Universidad de Salamanca, Salamanca, Spain; 5 Department of Biology, Faculty of Art and Science, Ondokuz Mayis University, Kurupelit-Samsun, Turkey; 6 Pacific Biosciences, 1380 Willow Rd, Menlo Park, California USA; 7 DOE Joint Genome Institute, Walnut Creek, California USA

## Abstract

There is a need to clarify relationships within the actinobacterial genus *Micromonospora*, the type genus of the family *Micromonosporaceae*, given its biotechnological and ecological importance. Here, draft genomes of 40 *Micromonospora* type strains and two non-type strains are made available through the *Genomic Encyclopedia of Bacteria and Archaea* project and used to generate a phylogenomic tree which showed they could be assigned to well supported phyletic lines that were not evident in corresponding trees based on single and concatenated sequences of conserved genes. DNA G+C ratios derived from genome sequences showed that corresponding data from species descriptions were imprecise. Emended descriptions include precise base composition data and approximate genome sizes of the type strains. antiSMASH analyses of the draft genomes show that micromonosporae have a previously unrealised potential to synthesize novel specialized metabolites. Close to one thousand biosynthetic gene clusters were detected, including NRPS, PKS, terpenes and siderophores clusters that were discontinuously distributed thereby opening up the prospect of prioritising gifted strains for natural product discovery. The distribution of key stress related genes provide an insight into how micromonosporae adapt to key environmental variables. Genes associated with plant interactions highlight the potential use of micromonosporae in agriculture and biotechnology.

## Introduction

Prokaryotic systematics is a core scientific discipline that encompasses classification, nomenclature, identification, and evolutionary processes^1^. The subject is practiced by few but its applications are relevant to most, if not all, microbiologists^2^. The discipline began as a largely empirical science but became increasingly objective due to the introduction of new concepts and practices, especially the development of chemotaxonomic, numerical phenetic and molecular systematic methods^3,4^. These developments led to the concept of polyphasic taxonomy, that is, the integrated use of genotypic and phenotypic data to generate classifications of prokaryotes^5^, an approach that was dependent on rapid data acquisition and improved data handling techniques^6,7^. Genotypic data tend to be derived from analyses of nucleic acids and phenotypic characteristics from chemotaxonomic, cultural, morphological and other expressed features^8^. The selection of methods for polyphasic studies, while critical, is somewhat subjective though 16S rRNA gene sequencing has proved to be a powerful tool for establishing relationships between prokaryotes at generic and suprageneric ranks^9–11^, but tends to be of limited use in distinguishing between closely related species^12–14^. In contrast, DNA-DNA pairing, molecular fingerprinting, multilocus sequence typing and phenotypic studies provide valuable data for circumscribing such species^14–18^. The widespread application of polyphasic taxonomy led to marked improvements in the classification of archaea and bacteria which, in turn, provided a sound basis for a stable nomenclature and improved identification, as exemplified by the current state of actinobacterial systematics^19^. The need to build upon these developments has been raised by those pressing for step-changes in prokaryotic systematics through “embracing the genome”^20–23^.

The application of low cost whole genome sequencing (WGS) technologies and associated bioinformatic tools is not only providing grist to the taxonomic mill^24–26^, but is furthering our knowledge of developmental and evolutionary processes^27–29^, as well as underpinning the ecological, physiological and biotechnological potential of prokaryotes^25,26,30–32^ thereby repositioning prokaryotic systematics as a fundamental scientific discipline. However, it is essential that taxonomies based on whole genome sequence data follow sound taxonomic practice, notably by following the nomenclatural type concept and the requirement to deposit type strains in two public culture collections in different countries^33,34^. In this context, the analysis of whole genome sequences of type strains under the auspices of the *Genetic Encyclopaedia of Bacteria and Archaea* (GEBA) project is greatly improving our understanding of phylogenetic relationships within and between these taxa, as well as generating an invaluable framework, technology and organisation for large scale genome sequencing of prokaryotes that will lead to an unprecedented coverage of prokaryotic diversity on the planet^35–39^. The application of innovative phylogenetic and taxonomic methods is also providing new metrics for the recognition of generic and species boundaries^20,23,40,41^, as well as resolving the structure of complex prokaryotic taxa, such as the actinobacterial genera *Amycolatopsis*, *Rhodococcus* and *Streptomyces* ^18,25,26^. Members of all of these taxa are a rich source of novel specialized metabolites, notably antibiotics^42,43^.

This study was designed to explore the extent to which whole genome sequence data derived from type strains of the genus *Micromonospora* can be used to clarify relationships within this taxon and provide insights into the biological properties and biotechnological potential of micromonosporae. The genus *Micromonospora* ^44^ is the type genus of the family *Micromonosporaceae* ^45^ of the order *Micromonosporales* ^46^; the family encompasses 31 validly named genera which can be distinguished using a combination of chemotaxonomic, morphological and phylogenetic criteria^45,47^. The genus was proposed by Ørskov in 1923^48^ for strains isolated from air that had been designated as “*Streptothrix chalcea*” by Foulerton^49^ and then reclassified as *Micromonospora chalcea*, the type species of the genus. At the time of writing the genus encompasses 79 species with validly published names (http://www.bacterio.net/micromonospora.html)^50^, the majority of which have been described using polyphasic methods^44,51^ though there is evidence that the taxon remains underspeciated^52,53^. Initially, micromonosporae were associated with soil, freshwater and marine habitats^44^ but novel strains have been isolated from animal^54–56^ and plant tissues^57–64^, as well as from limestone^65^, Antarctic sandstone^66^ and from a nickel mining site^67^. Micromonosporae form a tight cluster within the *Micromonosporaceae* 16S rRNA gene tree^44,51^ though 16S rRNA gene sequences are not sufficiently divergent to distinguish between closely related strains thereby drawing upon the need for associated DNA-DNA relatedness studies^68,69^. It is now apparent that phylogenies showing greater resolution between *Micromonospora* species can be generated using *gyr*B sequences^70^ and multilocus sequence analysis (MLSA) of housekeeping genes^53^.

Despite the advances outlined above there is a clear need to devise an improved framework for the classification and identification of *Micromonospora* strains, partly because of their importance in biotechnology, bioprospecting and ecology^42,44^. Amongst actinobacteria, micromonosporae are second only to streptomycetes in their ability to synthesize specialized metabolites; they are a particularly rich source of antibiotics, as exemplified by the production of the aminoglycosides: gentamicin, sagamicin, sisomicin and verdamicin from *Micromonospora purpurea* ^71^ (reclassified as *Micromonospora echinospora* ^70^), *Micromonospora sagamiensis* ^72^, *Micromonospora inyonensis* ^73^ and “Micromonospora grisea”^74^, respectively; everninomicin, an oligosaccharide antibiotic from *Micromonospora carbonacea* ^75^; the ansamycin antibiotic halomicin from *Micromonospora halophytica* ^76^; and the new macrolide antibiotics megalomicin^77^ and mycinamicin^78^ from *Micromonospora nigra* and “Micromonospora griseorubida”, respectively. Other specialized metabolites synthesized by micromonosporae include the antitumour compounds calicheamicin and lupinacidin C, these enediyne and anthraquinone antibiotics are produced by a *M. echinospora* NRRL 15839^79^ and *Micromonospora lupini* ^80^, respectively; and retymicin, galtamycin B, saquayamycin Z and ribofuranosyllumichrome from *Micromonospora* strain Tü 6368^81^. On a broader front *Micromonospora* strains have been considered to be a potential source of biocontrol agents, biofuels, plant growth products and plant probiotics^82–85^.

The metabolic potential of micromonosporae has been underlined in a few whole genome studies which show that a large proportion of the genetic potential of the tested strains code for the biosynthesis of natural products^85–87^. One of the drivers of the present study was to build upon these pioneering investigations to provide an insight into the potential of micromonosporae to produce new natural products thereby paving the way for developments in applied genomics with particular reference to genome mining^88–90^ and methods for activating silent biosynthetic gene clusters^91–93^. Investigations like these also highlight genomic features of potential ecological significance, as exemplified by the work on *M. lupini* strain Lupac 08, an endophyte able to colonise internal plant tissues^94^.

Here, whole genome sequences generated from 40 *Micromonospora* type strains and two strains related to *Micromonospora aurantiaca* and *M. echinospora* were generated and used to construct a phylogenomic tree together with the available genomes of *M. aurantiaca* ATCC 27029^T^ and L5^87^, and *M. lupini* Lupac 08^86^. The resultant data were used to determine the distribution of genes considered to code for natural products and for environmental adaptation, including stress responses. Little congruence was found between the structure of the phylogenomic tree and corresponding single gene trees based on 16S rRNA and conserved housekeeping gene sequences but congruence considerably increased when the single genes were combined in an MLSA of the conserved genes. The genomes of the strains were found to be rich in biosynthetic gene clusters many of which were discontinuously distributed. This study provides further evidence that the taxogenomic-approach to prokaryotic systematics can clarify relationships with complex actinobacteria taxa and provide invaluable insights into the biotechnological and ecological potential of the defined groups.

## Results

### General genome properties

**Figure 1:**
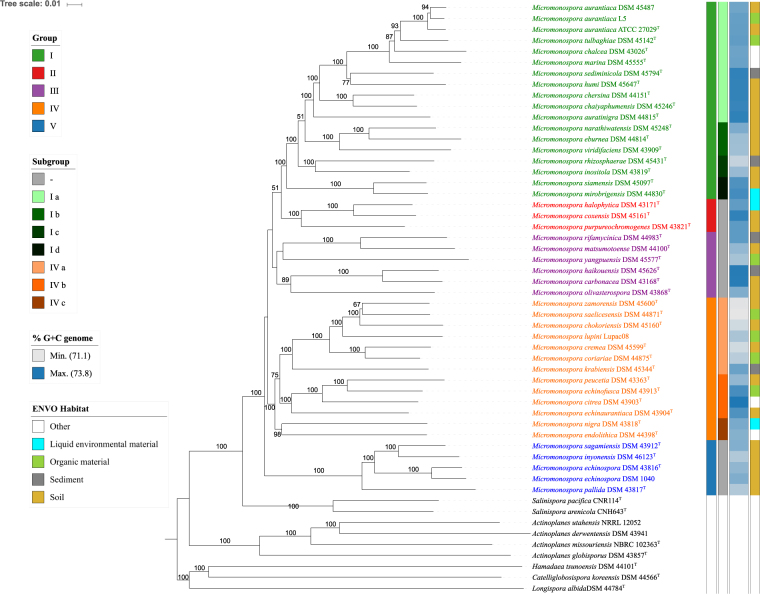
*Micromonospora* phylogeny inferred using the Genome BLAST Distance Phylogeny (GBDP) approach. The tree was inferred using the FastME from the GBDP intergenomic distances calculated from whole proteomes. The numbers above branches are GBDP pseudo-bootstrap support values from a 100 replicates, only values above 50% are shown. Tip colours on the right indicate the habitats from which the strains were isolated, those in the middle-right indicate genomic DNA G+C content, as embedded in the legends. Tip colours on the left indicate selected clades within the genus and those on the middle-left indicate well-supported subgroups within this clades.

**Figure 2:**
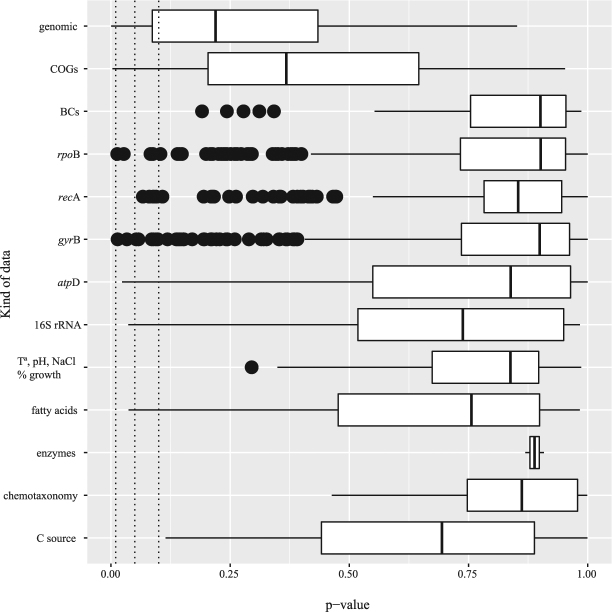
Tip permutation test analysis of *Micromonospora* features respect to phylogenomics. Shown are the p-values from the tests of individual characters arranged by kind of character. The dotted lines represent alpha levels (0.01, 0.05, 0.10). With exceptions for some fatty acids, no correlation was observed between phenotypic data (C source: carbon sources; chemotaxonomy: other than fatty acid data; enzyme production; fatty acids; and growth at several temperatures, pH and percentage of salinity) and genome scale phylogeny. The single genes (16S rRNA, *atp*D, *gyr*B, *rec*A, *rpo*B) presented few significant phylogenetically conserved characters. The number of BCs (biosynthetic gene clusters) classified by type of organic compounds presented no detectable phylogeny conservation, whereas some COGs categories and the GC content (as part of the group of other genomic characters) were significantly conserved.

High quality draft genomes were obtained for 40 *Micromonospora* type strains, 17 of which were completely closed. Approximate genome sizes of the investigated strains varied from 6.1 Mbp for *Micromonospora marina* DSM 45555^T^, a strain isolated from sea sand in Thailand^95^ to 7.9 Mbp for *M. carbonacea* DSM 43168^T^, a strain recovered from a soil sample in the United States^96^ (Fig. 1); the average genome size for all of the *Micromonospora* strains was 7 ± 0.4 Mbp (Supplementary Table 1). In the following sections, we report on gene numbers indicated by IMG annotation, but these numbers need to be interpreted cautiously as not all of the genome sequences were complete. The number of genes ranged from 5,550 in the genome of the type strain of *M. marina* to 7,388 in that of *Micromonospora cremea* DSM 45599^T^, a strain isolated from the rhizosphere of *Pisum sativum* ^97^. RNA genes represented 1–2% of the whole genome sequences ranging from 63 genes in the type strain of *M. aurantiaca* to 133 genes in *Micromonospora humi* DSM 45647^T^, a strain isolated from peat swamp forest soil^98^. An average of ten genes were identified as encoding rRNA’s (from 6 to 15 genes) and an average of 58 for tRNA’s (from 48 to 87 genes). The number of pseudogenes varied from 0 in 14 out of the 45 genomes up to 445 in the genome of the *M. cremea* type strain. The number of genes with a predicted function averaged 4,600, these ranged from 3,934 in the genome of *M. nigra* DSM 43818^T^ to 5,266 in that of *M. cremea* DSM 45599^T^. Between 4 and 10% of the genes were associated with the expression of signal peptides while the percentage of transmembrane proteins varied from 21 to 27% (Supplementary Table 1). The number of Clustered Regularly Interspaced Short Palindromic Repeats (CRISPR) rose from nought in the genome of *Micromonospora inositola* DSM 43819^T^ to over ten, as exemplified by *M. sagamiensis* DSM 43912^T^ (11), *Micromonospora yangpuensis* DSM 45577^T^ (12), *Micromonospora olivasterospora* DSM 43868^T^ (16), *Micromonospora peucetia* DSM 43363^T^ (17) and *Micromonospora viridifaciens* DSM 43909^T^ (18) with an average of five per genome (Supplementary Table 1). None of these genomic characteristics were found to be phylogenetically conserved (α = 0.01) in the tip permutation test (Fig. 2, Supplementary Table 2).

More than 40% of the average number of 6,361 genes detected in the *Micromonospora* genomes were conserved as the core genome. The size of the core genome corresponded to around 50% of the smallest genome, as exemplified by *M. marina* DSM 45555^T^. Large differences were found in the Clusters of Orthologous Groups (COGs), notably in categories G (carbohydrate transport and metabolism), K (transcription), N (cell motility), S (function unknown), W (extracellular structures), and X (mobilome: prophages, transposons) (Supplementary Figure 1; Supplementary Table 3). Particularly large variations were seen in category X, ranging from 123 genes in the *M. inyonensis* DSM 46123^T^ genome, which mainly coded for transposases, to four phage related proteins in the genome of *Micromonospora chersina* DSM 44151^T^, none of which coded for transposases (Supplementary Figure 1; Supplementary Table 3); however, it is known that the number of transposases can increase quickly through autoreplication^99^. For instance, one of the largest bacterial genomes encountered up to date, *Ktedonobacter racemifer* SOSP1–21^T^, contains a huge number of transposases^100^. Among the COG counts, categories I (lipid transport and metabolism) and M (cell wall/membrane biogenesis) were seen to be phylogenetically conserved (α = 0.01) in the tip-permutation test (Fig. 2, Supplementary Table 2).

### Genome based classification

It can be seen from the phylogenomic tree (Fig. 1) that the *Micromonospora* strains form a monophyletic group supported by a 100% bootstrap value. This taxon is clearly separated from an adjacent lineage that encompasses the type strains of *Salinispora arenicola* and *Salinispora tropica*. It is also evident from Fig. 1 that the *Micromonospora* strains fall into four well supported sublineages, groups I, II, IV, and V, and the less well supported strains that for the sake of clarity, have been classified into group III, a taxon that may prove to be heterogeneous. The largest taxon, group I, encompasses 18 strains, including *M. chalcea* DSM 43026^T^, the type strain of the type species of the genus *Micromonospora*. These strains were assigned to four subgroups that were supported by 100% bootstrap values, group Ia encompasses the three *M. aurantiaca* strains and the type strains of *Micromonospora auratinigra*, *M. chalcea*, *Micromonospora chaiyaphumensis, M. chersina, M. humi*, *M. marina, Micromonospora sediminicola* and *Micromonospora tulbaghiae*, closely related organisms isolated from sea sand^95^, marine sediment^101^, plants^59,87^, peat swamp forest^98,102^, air^49^ and soil^44,103,104^; group Ib is composed of the type strains of *Micromonospora eburnea*, *Micromonospora narathiwatensis* and *M. viridifaciens*, also from soil^68,105,106^; group Ic includes the type strains of *M. inositola* and *Micromonospora rhizosphaerae* from soil^107^ and rhizosphere^108^, respectively, and group Id the type strains of *Micromonospora mirobrigensis* and *Micromonospora siamensis*, two highly related organisms isolated, in turn, from a pond^109^ and peat swamp forest soil^110^.

Group II encompasses the type strains of *Micromonospora coxensis*, *M. halophytica* and *Micromonospora purpureochromogenes*, isolates from saline habitats^76,111^ and adobe soil^44^, respectively, and group III *M. carbonacea* DSM 43168^T^ and *Micromonospora haikouensis* DSM 45626^T^, two highly related strains isolated from soil^44,112^, *Micromonospora matsumotoense* DSM 44100^T^ and *Micromonospora rifamycinica* DSM 44983^T^ from rhizosphere soil^107,108^, *M. olivasterospora* DSM 43868^T^ from soil^113^ and *M. yangpuensis* DSM 45577^T^, an isolate from a sponge^56^ that lies towards the periphery of the taxon. Group IV, the second largest taxon, contains thirteen strains which were recovered in three subgroups, the first of which, IVa, contains *M. lupini* Lupac 08 and the type strains of *Micromonospora coriariae, M. cremea, Micromonospora saelicesensis* and *Micromonospora zamorensis*, all of which were isolated from ecto- and endo-rhizospheres^62,69,97^, and *Micromonospora chokoriensis* DSM 45160^T^ and *Micromonospora krabiensis* DSM 45344^T^ isolated from sandy and marine soils^111,114^; in turn, group IVb is composed of the type strains of *Micromonospora citrea, Micromonospora echinaurantiaca, Micromonospora echinofusca* and *M. peucetia*, isolates from soil, chukar excrement and lake mud^68^, respectively, while group IVc encompasses *Micromonospora. endolithica* DSM 44398^T^ and *M. nigra* DSM 43818^T^, strains recovered from Antarctic sandstone^44^ and a saline pond, respectively. Group V was composed of the two strains of *M. echinospora* and the type strains of *M. inyonensis, Micromonospora pallida* and *M. sagamiensis*, all of which were isolated from soil^44,68^.

The *in silico* DNA G+C content of the *Micromonospora* genomes fell within the range 71.1 to 73.8 mol % though narrower ranges are apparent within some groups, as exemplified by the group Ia strains, which showed values within the limit 72.8–73.6 mol % (Fig. 1). The tip permutation test (Fig. 2, Supplementary Table 2) indicated that the G+C content is phylogenetically conserved (α = 0.01) when calculated from the genome sequences. The genomes of 8 strains showed differences of more than one percent in G+C content when the *in silico* data were compared with results derived using experimental procedures, namely *M. aurantiaca* ATCC 27029^T^ (72.9% against 71.6%)*, M. coriariae* DSM 44875^T^ (71.8% against 70.2%)*, M. endolithica* DSM 44398^T^ (72.4% against 70%)*, M. haikouensis* DSM 45626^T^ (73.7% against 71.5%)*, M. matsumotoense* DSM 44100^T^ (72.3% against 71%)*, Micromonospora mirobrigenesis* DSM 44830^T^ (72.4% against 70%)*, M. rifamycinica* DSM 44983^T^ (73.3% against 68.6%) and *M. sediminicola* DSM 45794^T^ (73.6% against 74.8%). The *in silico* G+C contents of *M. citrea* DSM 43903^T^, *M. echinaurantiaca* DSM 43904^T^, *M. echinofusca* DSM 43913^T^, *M. inyonensis* DSM 46123^T^ *, M. peucetia* DSM 43363^T^ *, M. sagamiensis* DSM 43912^T^, *M. tulbaghiae* DSM 45142^T^ and *M. viridifaciens* DSM 43909^T^ were 73.8%, 73.2%, 73.3%, 71.9%, 72.3%, 72.5%, 73.0% and 72.1%, respectively; G+C contents had not been previously estimated for these strains.

Six pairs of *Micromonospora* type strains were considered to be closely related as their GBDP distances (the log-transformed ratios of the total number of non-identical amino-acids within the hits to the overall length of the hits in their genomes) were below 0.09; in each case digital DNA-DNA (dDDH) values were determined. Each pair, namely *M. coriariae* DSM 44875^T^ and *M. cremea* DSM 45599^T^, *M. carbonacea* DSM 43168^T^ and *M. haikouensis* DSM45626^T^, *M. coxensis* DSM 45161^T^ and *M. halophytica* DSM 43171^T^, *M. inyonensis* DSM 46123^T^ and *M. sagamiensis* DSM 43912^T^, *M. mirobrigensis* DSM 44830^T^ and *M. siamensis* DSM 45097^T^, was found to share dDDH similarities values below the recommended 70% cut-off for the delineation of species^115^, namely 53.8%, 59.1%, 52.2%, 69.8% and 53.6%, respectively. Consequently, all of these strains can be considered to represent *bona fide* species. The group encompassing *M. aurantiaca* ATCC 27029^T^ *, M. chalcea* DSM 43026^T^ and *M. tulbaghiae* DSM 45142^T^ gave the following dDDH values: 51.5% between *M. aurantiaca* ATCC 27029^T^ and *M. chalcea* DSM 43026^T^, 51.3% between *M. chalcea* DSM 43026^T^ and *M. tulbaghiae* DSM 45142^T^, and 60.1% between *M. aurantiaca* ATCC 27029^T^ and *M. tulbaghiae* DSM 45142^T^ indicating that all of these taxa should retain their species status. Corresponding dDDH values were obtained for the three *M. aurantiaca* strains: *M. aurantiaca* DSM 45487 and L5 shared 89.8% and 89.9% dDDH values with the type strain of *M. aurantiaca* and a 91.6% with one another indicating that they all belong to the same genomic species^115^. Similarly, *M. echinospora* DSM 43816^T^ and DSM 1040 are members of the same genomic species as they shared a 78.4% dDDH value.

### Insights from genome sequences

#### Classification

**Figure 3:**
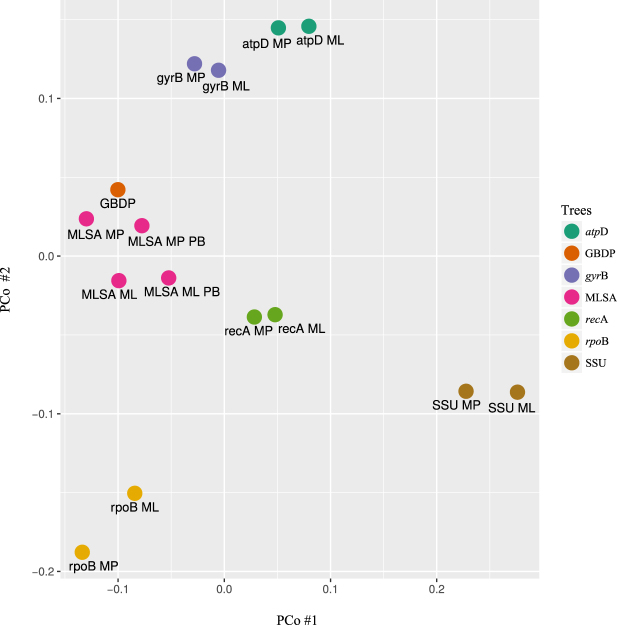
Principal coordinate analysis of topological distances. The analysis of the bootstrap-weighted relative Robinson-Foulds topological distances as calculated by RAxML shows that the lowest distances were between the whole genome sequence phylogeny (GBDP) and the MLSA phylogenies and the highest ones between the 16S rRNA gene phylogenies (SSU) and the GBDP tree; the distances with the other individual trees lay within these ranges. ML, maximum likelihood; MP, maximum parsimony; PB, partition bootstrap.

In general, little correlation was found between the groups circumscribed in the phylogenomic tree (Fig. 1) and those recovered in the trees based on single and concatenated gene sequences (Supplementary Figures 2–7). All of the groups were well supported in the GBDP analysis, apart from group III. Few of the groups delineated in the single and concatenated gene trees were supported by high bootstrap values though the five strains assigned to group V in the whole genome tree were supported by high bootstrap values in all of the other trees. The two largest groups recovered in the whole-genome tree, I and IV, were particularly fragmented in the individual and concatenated gene trees though the initial six strains assigned to group Ia, *M. aurantiaca* ATCC 27029^T^, DSM 45487 and L5, *M. chalcea* DSM 43026^T^, *M. marina* DSM 45555^T^ and *M. tulbaghiae* DSM 45142^T^, were found intact in all of the single and concatenated gene trees. Similarly, the type strains of *M. chokoriensis, M. coriariae, M. cremea, M. lupini, M. saelicesensis* and *M. zamorensis* (group IVa) were recovered with high bootstrap support in all but the 16S rRNA gene analysis. More importantly, it can be seen from the principal coordinates plot (Fig. 3) that there is a closer correspondence between the whole genome and MLSA trees than with any of the those based on individual gene sequences, notably with respect to the 16S rRNA gene tree. These results are in line with those from the tip-permutation test which revealed comparatively few significantly phylogenetically conserved characters within these genes (Fig. 2). Concatenating such moderately informative genes apparently had the expected effect that the signal added up whereas the noise cancelled out^20^.

#### Phenotypic properties

The *Micromonospora* strains share similar chemotaxonomic and morphological features. All of them contain *meso*-diaminopimelic in the cell wall peptidoglycan, xylose in whole-organism hydrolysates, complex mixtures of *iso*- and *anteiso*- branched fatty acids with predominant proportions of *iso*-C_15:0_ and *iso*-C_16:0_ and polar lipid patterns containing phophatidylethanolamine (diagnostic lipid), as shown in Supplementary Figure 8 and Supplementary Table 4. Most of the strains contain tetra- and hexa-hydrogenated menaquinones with ten isoprene units (MK-10 [H_4_, H_6_]), as shown in Supplementary Figure 8. In general, the strains grew at 20 and 37 °C, at pH 8.0 and 9.0 and in the presence of 1%, w/v sodium chloride, and produced catalase, hydrolysed aesculin and arbutin, degraded casein, starch, Tween 20 and xylan, but do not grow at 4 °C, pH 4.4 or in the presence of 5%, w/v sodium chloride (Supplementary Table 5). The tip permutation test showed that there was little evidence of correlation between the distribution of phenotypic features within the genus *Micromonospora* and the topology of the phylogenomic tree (Fig. 2, Supplementary Table 2). The lowest p-value recorded was for *iso*-C_17:0_ (0.0394).

There was little sign that the distribution of phenotypic properties across the *Micromonospora* strains was influenced by the habitats from which they were isolated (Supplementary Figure 8, Supplementary Table 6) though none of the isolates from aquatic habitats contained arabinose, rhamnose or ribose in whole-organism hydrolysates or used trehalose or xylose as sole carbon sources (Supplementary Figure 8). There was some indication that strains from habitats rich in organic matter, notably from plant tissues, grew within a narrower pH range than those from soil samples, at 4 °C and used alanine, propionic acid and valine as sole carbon sources (Supplementary Figure 8). The Chi-2-test shows that some of the phenotypic features were correlated, mainly due to the presence of common pathways, as exemplified by the utilization of cellobiose, melibiose, maltose and raffinose as carbon sources (Supplementary Figure 9).

#### Genes potentially associated with environmental adaptation

The genomes of *M. citrea* DSM 43903^T^, *M. coxensis* DSM 45161^T^ *M. echinofusca* DSM 43913^T^, *M. endolithica* DSM 44398^T^, *M. halophytica* DSM 43171^T^, *M. marina* DSM 45555^T^, *M. mirobrigensis* DSM 44830^T^, *M. nigra* DSM 43818^T^ and *M. siamensis* DSM 45097^T^, isolates from diverse habitats (Supplementary Table 6), contained genes associated with photosynthesis, as described for marine bacteria^116^; these genes belong to the proteorhodopsin family, which includes light-regulated transmembrane proteins. The genomes of all of these strains contained genes associated with the production of ß-carotene ketolase (*crt*O), lycopene ß-cyclase (*crt*Y), octaprenyl diphosphate synthase (*isp*B), phytoene dehydrogenase (*crt*I), phytoene synthase (*crt*B), proteorhodopsin (*pro*t) and spheroidene monooxygenase (*crt*A). Similarly, the genomes of the *M. coxensis, M. echinosfusca, M. halophytica* and *M. siamensis* strains include genes that encode for sensory rhodopsin II (SRII). The genomes of these organisms also contained 15 out of 25 genes implicated in carbon fixation in photosynthetic bacteria, 20 out of 41 genes associated with glycolysis/gluconeogenesis, 12–14 out of 58 genes associated with dicarboxylate and glyoxylate metabolism, 19 out of 31 genes implicated in phenylalanine, tyrosine and tryptophan biosynthesis and 11 out of 13 genes associated with CO_2_ fixation, according to the KEGG (Kyoto Encyclopedia of Genes and Genomes) pathway database^117^ (Supplementary Table 10). It is also evident from this Table that the genomes of 23 of the strains contained a CO dehydrogenase maturation factor gene (*cox*F) associated with CO_2_ fixation. In addition, the genomes of *M. aurantiaca* DSM 45487 and L5, *M. chalcea* DSM 43026^T^ and *M. tulbaghiae* DSM 45142^T^ contained a *cox*D gene, which codes for a carbon monoxide oxidation accessory protein (Supplementary Table 10).

*Micromonospora* strains are rich in degrading enzymes (Supplementary Table 10), as exemplified by the ability of the plant endophyte *M. lupini* Lupac 08 to produce amylases, cellulases, chitinases, pectinases and xylanases^94^. The genomes of all of the *Micromonospora* strains contained genes coding for amylases, notably α-amylases and glucoamylases. All of the micromonosporal genomes presented genes associated with cellulase production with the exception of those of *M. echinaurantiaca* DSM 43904^T^ *, M. inositola* DSM 43819^T^ and *M. peucetia* DSM 43363^T^. The genomes of *M. chalcea* DSM 43026^T^ *, M. chokoriensis* DSM 45160^T^ *, M. eburnea* DSM 44814^T^, *M. echinospora* DSM 1040 and DSM 43816^T^ *, M. haikouensis* DSM 45626^T^ and *M. rifamycinica* DSM 44983^T^ contained a gene encoding for a putative secreted cellulase. In turn, all of the genomes contained *chi*C genes, coding for chitinases, the number of these genes ranged from three in the genomes of *M. pallida* DSM 43817^T^ and *M. rhizosphaerae* DSM 45431^T^ up to 13 in the genome of *M. cremea* DSM 45599^T^. The genomes of most of the *Micromonospora* strains contained genes associated with the production of pectate lyases, including the *hrp*W gene, which codes for a harpin secreted effector that elicits the hypersensitive response in plants^118^, this gene was detected in *M. coxensis* DSM 45161^T^, *M. echinofusca* DSM 43913^T^ and *M. yangpuensis* DSM 45577^T^. The genomes of *M. aurantiaca* ATCC 27029^T^, DSM 45487 and L5, *M. carbonacea* DSM 43168^T^, *M. echinospora* DSM 43816^T^, *M. haikouensis* DSM 45626^T^, *M. matsumotoense* DSM 44100^T^, *M. rifamycinica* DSM 44983^T^ and *M. sagamiensis* DSM 43912^T^ also presented genes coding for pectinesterase. Similarly, all but the type strain of *M. olivasterospora* have genomes associated with the production of xylanases, notably for endo-1,4-β-xylanase A precursors with an average of 21 genes per genome. All of the micromonosporal genomes contained genes that code for ß-phosphoglucomutases, enzymes associated with starch degradation, as well as those that encode for trehalose phosphorylases, enzymes associated with trehalose degradation^94^. The genomes of the type strains of *M. carbonacea, M. chokoriensis, M. haikouensis, M. humi, M. lupini, M. matsumotoense, M. rifamycinica, M. saelicesensis* and *M. zamorensis* contained the trehalase gene (*tre*A) while that of *M. pallida* DSM 43817^T^ was alone in coding for trehalose 6-phosphate hydrolase (*tre*C).

The *Micromonospora* strains have the capacity to produce plant-related hormones (Supplementary Table 10). The genomes of all of the strains contained genes predicted to code for indole-3-glycerol phosphate synthase (*trp*D), an intermediate in the tryptophan synthetic pathway associated with the production of indol-acetic acid (IAA) which stimulates plant growth^119^. In addition, the genomes of most of the strains contained genes that coded for acetoin synthesis, which induces systemic resistance in *Arabidopsis* ^120^, exemplified by the acetolactate synthase large and small subunit genes, as well as for a gene enconding for acetoin dehydrogenase; *M. aurantiaca* L5, *M. citrea* DSM 43903^T^, *M. coriariae* DSM 44875^T^, *M. cremea* DSM 45599^T^, *M. nigra* DSM 43818^T^, *M. olivasterospora* DSM 43868^T^, *M. rhizosphaerae* DSM 45431^T^, and *M. yangpuensis* DSM 45577^T^ lack this gene. The genomes of the group V strains (*M. echinospora* DSM 1040 and DSM 43816^T^, *M. inyonensis* DSM 46123^T^, *M. pallida* DSM 43817^T^ and *M. sagamiensis* DSM 43912^T^) included genes predicted to produce 2,3-butanediol dehydrogenase, an enzyme associated with the plant growth promoting hormone 2,3-butanediol and acetoin production^121^. However, only *M. coriariae* DSM 44875^T^ and *M. krabiensis* DSM 45344^T^ have genes predicted to encode for 1-aminocyclopropane-1-carboxylate (ACC) deaminase, a plant-growth promotor associated with the reduction of ethylene levels which lead to a reduction in plant stress^122^. Other characteristics involved in plant growth promotion include the ability to solubilize phosphates and the production of siderophores that scavenge phosphate and iron from soil making them available for plants^122^; genes coding for the production of phosphatases and siderophores were detected in all of the *Micromonospora* genomes. In contrast, none of the genomes contained genes associated with the ability to fix atmospheric nitrogen.

The *Micromonospora* strains produced a well-developed substrate mycelium that carried single spores either directly or on short sporophores. None of the strains formed aerial hyphae though the genomes of almost half of them showed the presence of a predicted surface active peptide cluster (*sap*B) that encodes for a lantabiotic-like peptide which has been considered to trigger the formation of aerial hyphae when strains are grown on rich media^123^. Other genes related to sporulation were found in all of the strains, namely *whi*B and *whi*D genes, which are required for the differentiation of aerial hyphae into mature spores in *Streptomyces* ^124^. Another characteristic of *Micromonospora* strains is their ability to produce a range of pigments at the onset of spore production. The genomes of all of the tested strains, apart from *M. cremea* DSM 45599^T^, contained *whi*E-ks, *whi*E-clf, *whi*EI, *whi*EII, *whi*EVI, and *whi*EVII genes which are associated with spore pigment production in *Streptomyces* ^125^. All of the strains contained genes coding for the production of pigments, as well as biosynthetic gene clusters associated with the production of carotenoid, isorenieratene and sioxanthin compounds (Supplementary Table 7). Between the genes implicated in carotenoid production pathway detected in most of the *Micromonospora* genomes were the putative genes encoding for β-carotene ketolases, phi-carotenoid synthases, geranylgeranyl pyrophosphate synthetases, lycopene cyclases, phytoene synthases and squalene-hopene cyclases (Supplementary Table 10).

The genomes of the *Micromonospora* strains contained a range of genes associated with DNA repair systems (Supplementary Table 10). All of the genomes included at least one copy of excinuclease subunits A, B and C (*uvr*A, *uvr*B, *uvr*C genes) and three copies of ATP-dependent DNA helicase (*uvr*D), one of which has only been associated with actinobacteria (*uvr*D-actino). The *Micromonospora* genomes were also rich in *rec* genes, implicated in recombination, in the production of helicases, and for general DNA repair, but only the genome of *M. nigra* DSM 43818^T^ presented the *rec*B gene, which codes for an exodeoxyribonuclease. Similarly, all of the genomes contained exodeoxyribonuclease genes (*xse*A, *xse*B, and *exo*III), as well as genes associated with the production of several exo- and endonucleases, namely *sbc*C, *sbc*D, *end*1, *endo*IV, *endo*V.

#### Genes associated with stress responses

The genomes of *Micromonospora* strains annotated by RAST^126,127^ and analyzed through the SEED viewer^128^ showed between 115 and 144 putative genes known to be associated with stress responses, notably those encoding for carbon starvation, heat shock responses, osmoregulation and oxidative stress (Supplementary Table 10). The genomes of all of the strains contained *csp*A and *csp*C genes, which encode for families of proteins that respond to cold shock^129^, and *dna*K, *grp*E and *hrc*A genes involved in heat shock responses^130^. In contrast, *csp*G genes, that encode for a cold shock protein associated with cellular SOS repair systems^131^, were restricted to the genomes of *M. aurantiaca* DSM 45487, *M. echinospora* DSM 43816^T^ and *M. krabiensis* DSM 45344^T^. All of the *Micromonospora* genomes contained *bet*C and *pro*U genes which govern the uptake of betaine and choline, metabolites that contribute to responses to oxidative stress^132,133^. Other universally distributed stress related genes include those that encode for alkyl hydroperoxidase reductases^134^ (*ahp*C genes), redox sensitive transcriptional regulators^135,136^ (*rex* and *sox* genes), iron-stress related *fur* genes^137^, and the nitric oxide dioxygenase gene (*hmp*X), which is induced by the presence of NO and prevents the inhibition of growth caused by nitrosative stress^138^; around half of the genomes showed the presence of superoxide dismutase genes (*sod*). Genes associated with ectoine biosynthesis (*ect*) for osmoregulation were found in the genomes of six *Micromonospora* strains (Supplementary Table 10); genes encoding for diaminobutyrate-pyruvate aminotranferases (*ect*B genes) were present in the genomes of *M. chersina* DSM 44151^T^ *, M. echinospora* DSM 43816^T^, *M. endolithica* DSM 44398^T^ *, M. matsumotoense* DSM 44100^T^ and *M. peucetia* DSM 43363^T^, the genome of the remaining strain, *M. eburnea* DSM 44814^T^ contained *ect*C genes that encode for L-ectoine synthases. The genomes of most of the *Micromonospora* strains contained *rsp*A genes that code for starvation sensing protein A which may help them to survive in low carbon habitats by activating peptide uptake^139–141^; these genes were not detected in the genomes of the type strains of *M. auratinigra, M. coxensis, M. halophytica, M. inyonensis, M. marina, M. nigra, M. olivasterospora, M. sagamiensis, M. sediminicola* or *M. siamensis* (Supplementary Table 10).

#### Biosynthetic gene clusters coding for specialized metabolites

**Figure 4:**
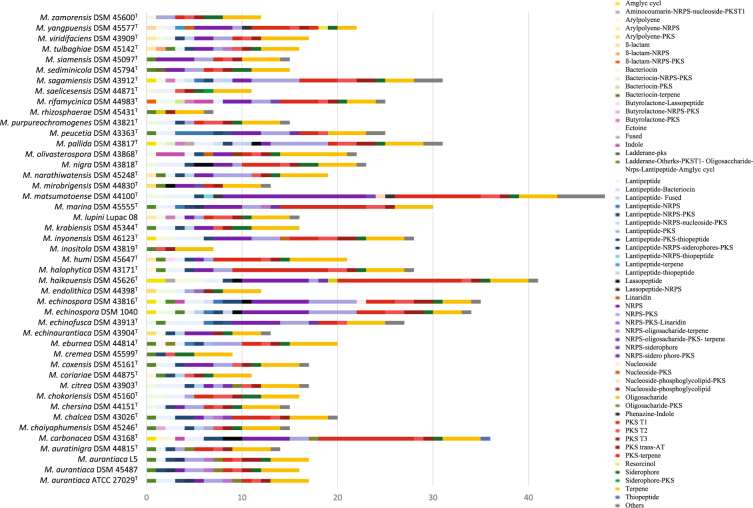
Biosynthetic gene clusters found in the *Micromonospora* genomes using antiSMASH 3.0. Highly variable profiles were found between the strains. The genomes of the *Micromonospora* strains were found to be especially rich in NRPS, PKS and terpene clusters; whereas there was also an abundance of bacteriocin, lantipeptide and siderophores clusters.

All of the *Micromonospora* genomes were screened for candidate biosynthetic gene clusters using the specialized metabolite identification pipeline antiSMASH. The number of such putative bioclusters ranged from 7 in the genomes of *M. cremea* DSM 45599^T^ and *M. rhizosphaerae* DSM 45431^T^ to 48 in that of *M. matsumotoense* DSM 44100^T^; the genomes of the type strains of *M. carbonacea, M. echinospora, M. haikouensis, M. marina, M. pallida* and *M. sagamiensis* were also rich in such biosynthetic gene clusters (Fig. 4). The average numbers of biosynthetic gene clusters detected in the genomes of the *Micromonospora* strains was twenty, most of which seem to be related with antibiotic, siderophore and terpene production (Fig. 4, Supplementary Table 7). Just over 22% of the biosynthetic gene clusters present in the micromonosporal genomes (206 out of 915) lacked any homology with known bioclusters; these biosynthetic gene clusters belong to several cluster types, notably terpenes (82), non ribosomal peptides (27), lantipeptides (26) and polyketide synthases (16) (Fig. 4, Supplementary Table 7). The remaining bioclusters showed similarities to a greater or lesser extent for known compounds though most of them (85%) showed less than 75% similarity of their genes with known bioclusters. A total of 172 different bioclusters were detected in all the genomes, most of them related to antibiotic production though only 33 presented a similarity of over 50% with known compounds (Supplementary Table 7). Thirteen of these compounds were related to known antibiotics (actinorhodin, chloramphenicol, diazepinomicin, leucanicidin, livipeptin, lobosamide, micromonolactam, rishirilide B, salinilactam, sibiromycin, streptazone tiacumicin B, TLN-05220) with similarity values of their genes over 75% (Supplementary Table 7).

Two biosynthethic gene clusters were found in the genomes of all of the *Micromonospora* strains, one related to the production of alkyl-O-dihydrogeranyl-methoxyhydroquinone (with ~70% similarity) and the other with a bacteriocin-terpene related to the production of lymphostin (with ~40% similarity). Similarly, the sioxanthin biosynthetic gene cluster was found in all of the *Micromonospora* strains, apart from *M. inositola* DSM 43819^T^ and *M. pallida* DSM 43817^T^. In contrast, most of the biosynthetic gene clusters had a limited distribution, 83 were found in a single genome, as exemplified by the one related to the carotenoid biosynthetic gene cluster present in the *M. pallida* genome; 34 biosynthetic gene clusters were detected in just two of the *Micromonospora* genomes, as illustrated by the chlorothricin biosynthetic gene cluster present in the genomes of the type strains of *M. eburnea* and *M. endolithica*. The sequences of five biosynthetic gene clusters, apart from the one encoding for sioxanthin, were identical to ones known to code for geosmin^142^ as found in the genome of *M. pallida* DSM 43817^T^; leucanicidin, a potent nematocide^143^, as seen in the genome of *M. carbonacea* DSM 43168^T^; livipeptin, an aldehyde peptide^144^, as detected in the genomes of *M. echinofusca* DSM 43913^T^ and *M. peucetia* DSM 43365^T^; micromonolactam, a polyene macrolactam^145^, as found in the genome of *M. haikouensis* DSM 45626^T^ while SapB, which is associated with aerial hyphae formation^123^, was found in almost half of the *Micromonospora* genomes (Supplementary Table 7).

The genomes of several *Micromonospora* strains assigned to taxa defined in the whole genome tree (Fig. 1) included specific biosynthetic gene clusters associated with the synthesis of known bioactive compounds. This was particularly so with the group V strains, namely *M. echinospora* DSM 1040 and DSM 43816^T^, *M. inyonensis* DSM 46123^T^, *M. pallida* DSM 43817^T^ and *M. sagamiensis* DSM 43912^T^. The genomes of these strains contain bioclusters which show similarities against (i) feglymycin, a peptide antibiotic produced by *Streptomyces* sp. DSM 11171^146^ that inhibits HIV cell to cell transfer^147^ (this biosynthetic gene cluster was also detected in the genome of *M. rifamycinica* DSM 44983^T^); (ii) gentamicin produced by *M. echinospora* NRRL 2953 and NRRL 2985^T 71^ (this biocluster was also found in the genomes of *M. carbonacea* DSM 43168^T^, *M. haikouensis* DSM 45626^T^, *M. matsumotoense* DSM 44100^T^, *M. peucetia* DSM 43363^T^ and *M. yangpuensis* DSM 45577^T^; (iii) herbimycin, isolated from *Streptomyces* sp. RM-7–15^148^, which shows activity against herbs and heat shock protein 90, this biosynthetic gene cluster was present in the genome of *M. narathiwatensis* DSM 45248^T^, and (iv) TLN-05220, a product of *M. echinospora* NRRL 1225, which shows activity against methicillin-resistant strains of *Staphylococcus aureus*, vancomycin-resistant enterococci and several human cell lines^149^. Further, apart from the type strain on *M. inyonensis*, the genomes of the group V strains had a biosynthethic gene cluster related to crocacin, an electron transport inhibitor isolated from *Chondromyces crocatus* CM c3 that inhibits Gram-positive bacteria and fungi^150^ while the genomes of *M. echinospora* DSM 1040 and *M. sagamiensis* DSM 43912^T^ contain a biocluster associated with the production of muraymycin, a nucleoside-lipopeptide antibiotic synthesized by *Streptomyces* sp. LL-AA896 that inhibits peptidoglycan biosynthesis^151^.

The genomes of the initial six strains of the group 1a strains, namely *M. aurantiaca* ATCC 27029^T^, DSM 45487 and L5, *M. chalcea* DSM 43026^T^, *M. marina* DSM 45555^T^, and *M. tulbaghiae* DSM 45142^T^, include biosynthetic gene clusters with the potential to synthesize known specialized metabolites. These strains, apart from *M. chalcea* DSM 43026^T^, contain a biocluster which present some similarity with the biocluster responsible of leinamycin production, a potent antitumor antibiotic produced by *Streptomyces* strains^152^ (this biosynthetic gene cluster is also present in the genomes of *M. haikouensis* DSM 45626^T^, *M. matsumotoense* DSM 44100^T^ and *M. purpureochromogenes* DSM 43821^T^). In turn, the genomes of all but the *M. marina* strain contain a biocluster related to nocathiacin, a thiazole peptide antibiotic produced by *Nocardia* sp. WW-12651^153^, derivatives of which have been used to treat multidrug-resistant bacterial infections^154^ although in this instance the similarity between the two bioclusters is low (this biocluster is also present in the genomes of *M. coxensis* DSM 45161^T^, *M. humi* DSM 45647^T^, *M. peucetia* DSM 43363^T^ and *M. sediminicola* DSM 45794^T^). The genomes of the three *M. aurantiaca* strains have a biocluster associated with the production of dynemicin, a 1,5-diyn-3-ene-containing antibiotic produced by *M. chersina* ^155^ with antibacterial and antitumor activities (this biocluster was also detected in the genomes of *M. chersina* DSM 44151^T^ and *M. yangpuensis* DSM 45577^T^). However, none of the different types of biosynthetic gene clusters appeared to be phylogenetically conserved (α = 0.01) in the tip permutation test (Fig. 2, Supplementary Table 2). The distribution of bioclusters was not related either to the origin of the strains, though there was an average of 21 bioclusters in the genomes of the strains isolated from soil, sediment and liquid environments strains and 17 in the genomes of those isolated from organic material. In contrast, only the genome of *M. marina* DSM 45555^T^ contained the kiamycin biosynthetic cluster which has been detected in marine-related organisms^156^.

With a single exception all of the genomes showed the capacity to synthesize the seven enzymes (DAHP synthase, 3-dehydroquinate synthase, 3-dehydroquinate dehydratase, shikimate dehydrogenase, shikimate kinase, EPSP synthase, and chorismate synthase) implicated in the shikimate pathway, which has been previously related to the production of aromatic antibiotics^157^; the exception was the type strain of *M. auratinigra*, which do not have the ability to produce shikimate kinase (Supplementary Table 10). Similarly, almost half of the genomes have the capacity to encode 3-amino-5-hydroxybenxoic acid (AHBA) synthase, involved in the synthesis of the precursor of mD_7_N units in several antibiotics^158^ (Supplementary Table 10).

## Discussion

The results of this study provide further evidence that data generated in whole genome sequencing studies provide an essential framework for the reclassification of taxonomically complex prokaryotic taxa previously defined from analyses of relatively few taxonomic features^25,26,159^. It is evident from the *Micromonospora* phylogenomic tree that the tested strains not only form a monophyletic group but fall into several well supported phyletic lines, only two of which were recognised in their entirety in the corresponding trees based on single gene sequences. The six initial members of group 1a, namely *M. aurantiaca* ATCC 27029^T^, DSM 45487 and L5, *M. chalcea* DSM 43026^T^, *M. marina* DSM 45555^T^ and *M. tulbaghiae* DSM 45142^T^, were defined in the *atp*D, *gyr*B, *rec*A, *rpo*B and 16S rRNA gene trees, as well as in the MLSA tree based on all of the individual gene sequences. Similarly, the group V strains, *M. echinospora* DSM 1040 and DSM 43816^T^, *M. inyonensis* DSM 46123^T^, *M. pallida* DSM 43817^TT^, and *M. sagamiensis* DSM 43912^T^, was recovered in all the phylogenetic trees.

Genomic DNA G+C content, that is, the proportion of cytosine and guanine moieties over the overall number of nucleotides in the genome, feature prominently in the description of prokaryotic genera and species^8,160,161^. DNA base composition values based on the application of conventional methods are considered to be indirect values as they do not count nucleotides, but estimate genomic G+C content from physical properties drawn from analyses of extracted and/or digested DNA^162^. However, it is becoming increasingly apparent that estimates of G+C content taken directly from whole genome sequences are of higher quality than those derived from well known experimental methods^163^. Indeed, these workers have shown that strains within a species have G+C values within a 1% range. It is, therefore, encouraging that in the present study a statistically sound relationship was found between *in silico* G+C values and the distribution of the *Micromonospora* strains within the phylogenomic tree. Moreover, *in silico* G+C values of the tested type strains fell within the range 71.1–73.8 mol % with narrower ranges found for strains assigned to well supported phyletic lines, as exemplified by the group 1a and IVa strains which showed values of 72.8–73.6 and 71.1–72.0 mol %, respectively. The genomes of eight of the *Micromonospora* type strains showed more than a 1% difference when *in silico* G+C values where compared with corresponding results found using conventional laboratory based methods. It is important to resolve such discrepancies between G+C values so that differences between closely related species are not obscured^159,164^. Emended descriptions are given for these *Micromonospora* species and for an additional eight species that previously lacked estimates of DNA G+C values.

DNA-DNA hybridization (DDH) is still widely used to estimate genetic relatedness between closely related bacteria as it is seen to be the “gold standard” for species delineation between prokaryotes^165^. Indeed, the recommendation of Wayne and his colleagues that a DDH of 70% for the prokaryotic species boundary has been widely followed by the systematic community^115^. It is now evident that dDDH methods based on comparisons of whole genome sequences provide better quality data for discriminating between closely related strains than corresponding values derived from the application of experimental methods that are well known to be expensive, labour-intensive and prone to experimental error^166–168^. dDDH values estimated from the genomes of the six pairs of closely related type strains showed that they fell below the 70% threshold^115^ indicating that *M. coriariae* ^62^ and *M. cremea* ^97^, *M. carbonacea* ^96^ and *M. haikouensis* ^112^, *M. coxensis* ^111^ and *M. halophytica* ^76^, *M. mirobrigensis* ^109^ and *M. siamensis* ^110^, *M. inyonensis* ^68^ and *M. sagamiensis* ^68^ are validly named species.

Kroppenstedt and his colleagues^68^ recognised that the type and only representatives of *M. inyonensis* and *M. sagamiensis* were closely related but could be distinguished based on cultural and phenotypic properties, by their fatty acid and MALDI-TOF mass spectrometric profiles and by a DDH value of 61.3%^115^. In the present study, these strains were found to share a dDDH value marginally below the recommended cut-off point, but were distinguished readily by the number and type of their biosynthetic gene clusters, by the presence of different stress genes in their genomes and by differences in the composition of nine of the COG groups, notably those belonging to the categories G, R and X. In light of all of these data it can be concluded that *M. inyonensis* and *M. sagamiensis* strains belong to different, but closely related species.

The family *Micromonosporaceae* encompasses several genera, such as *Salinispora* ^169,170^, that are difficult to distinguish from *Micromonospora* strains using conventional genotype and phenotype procedures^45,171^. The phylogenomic classification of the representative *Micromonospora* type strains not only provides a framework for clarifying relationships with those from related genera but also allows the taxonomic provenance of *Micromonospora* strains to be established. It is interesting that the genera *Micromonospora* and *Salinispora* are quite sharply separated, albeit closely related, in the phylogenomic tree though the genomes of additional representatives of these taxa need to be examined to underscore precise relationships between them. It is encouraging that the authenticity of *M. aurantiaca* DSM 45487 and L5, and *M. echinospora* DSM 1040 were confirmed in the present study.

There were few signs of concordance between the distribution of chemotaxonomic and other phenotypic markers drawn from the original descriptions of the *Micromonospora* type strains and their assignment to taxa in the phylogenomic tree. This lack of congruence can be attributed to factors such as the use of such a small sample of strains and tests, reliance on inappropriate and/or unreliable phenotypic tests and failure to use appropriate reference material. Sutcliffe and his colleagues have stressed the need to address such issues. A better understanding of the relationship between genotype and phenotype can be expected to provide a way forward on such matters. In sharp contrast to the issues raised above, all of the type strains produced whole organism hydrolysates rich in *meso*-diaminopimelic acid and xylose, major amounts of saturated and unsaturated fatty acids, notably iso-C_15:0_ and iso-C_16:0_, a polar lipid pattern containing phosphatidylethanolamine (diagnostic lipid) and usually diphosphatidylglycerol and phosphatidylinositol (phospholipid pattern 2 *sensu* Lechevalier *et al*.^172^) and tetra- and hexa- hydrogenated menaquinones with either nine or ten isoprene units as predominant isoprenologues. Such genus specific properties are of particular value in distinguishing *Micromonospora* from most of the other genera classified in the family *Micromonosporaceae* ^45^. Similarly, many of the *Micromonospora* strains share phenotypic features, as exemplified by their ability to hydrolyse aesculin and arbutin and degrade casein, starch, Tween 20 and xylan. In contrast, very few of the *Micromonospora* strains grew at 4 °C, pH 4.4 or in the presence of 5% w/v sodium chloride.

In general, *Micromonospora* species have been associated with aquatic and terrestrial habitats across diverse geographical regions thereby underscoring their adaptability^44^. More recently, they have been recovered from the tissues of a broad range of plants^173–175^. In the present study, little correlation was found between the source of the *Micromonospora* strains and their distribution to taxa delineated in the phylogenomic tree. However, it is interesting that all of the strains isolated from ecto- or endo-rhizospheres, namely *M. coriariae* DSM 44875^T^ from a root nodule of *Coriaria myrtifolia*, *M. lupini* Lupac 08 and *M. saelicesensis* DSM 44871^T^ from root nodules of *Lupinus angustifolius*, and *M. cremea* DSM45599^T^ and *M. zamorensis* DSM 45600^T^ from the rhizosphere of *Pisum sativum* were recovered in the well delineated subgroup IVa. Associations such as these would be much easier to establish if more details were given on the sources of strains in species description of prokaryotes. Indeed, such information is a prerequisite for data-driven prokaryotic systematics^176^.

It is now well known that micromonosporae are associated with roots of diverse plant species^173–175^, notably nodules of healthy leguminous plants^53,57,69,94,177^. The discovery that *Micromonospora* strains occupy nitrogen-fixing nodules poses several intriguing questions such as whether they are in transition from a saprophytic to a facultatively endophytic lifestyle and whether they have a beneficial effect on the plant. In general, deductions drawn from the genomes of the tested strains underpin key genome features captured by Trujillo and her colleagues for *M. lupini* strain Lupac 08^94^. Some, if not all of the genomes of the *Micromonospora* strains, like the *M. lupini* strain, have putative genes that encode for acetoin, 2,3-butanediol dehydrogenase and indol-3-acetic acid, auxinic phytohormones implicated in phytostimulation^119,122,178,179^. Along similar lines, the genomes of all of these strains are rich in putative genes that code for antibiotics, chitin degradation and siderophores, compounds that may contribute to the defence of the host plant against root infecting fungi. It is also interesting that the genomes of a few *Micromonospora* strains, including *M. lupini* strain Lupac 08, contained genes encoding trehalase, an enzyme that degrades trehalose and is implicated in nodule growth regulation^180,181^. All of these observations indicate that micromonosporae confer protection to the plant. It has also been shown that inoculation of strain Lupac 08 into legumes contributes to the welfare of the host plant^94^. An important conclusion drawn from this study is that micromonosporal genomes lack *nif*H-like fragments, despite early claims to the contrary^174,177^.

It is still too early to draw far reaching conclusions about the ecological roles of facultatively endophytic micromonosporae, as their genomes have an array of putative genes that code for degradative enzymes involved in the turnover of plant polymers, notably amylases, cellulases, chitinases, pectinases and xylanases. Indeed, *Micromonospora* strains may have the capacity, proven in the case of strain Lupac 08, to produce a range of degradative enzymes that are characteristic of saprophytic bacteria. This picture is clouded even further as the genomes of the *Micromonospora* strains isolated from diverse habitats encoded for much the same traits as the endophytic strains. It could be that micromonosporae have the capacity to colonize multiple ecological niches though additional studies are required to address this point.

There are several reports that *Micromonospora* strains can form sterile aerial hypahe^45,182–185^ and one which presented evidence that on certain nutrient media micromonosporae from marine sediments form aerial mycelia that can be used to propagate fresh colonies^186^. Baldacci and Locci^187^ found that strains designated as “Micromonospora melanosporea” formed aerial mycelia with short branching sporophores bearing single spores. In light of these observations it is interesting that the genomes of many of the *Micromonospora* type strains showed the presence of putative genes associated with aerial hyphae formation and spore maturation in streptomycetes^123,125,188^ though *whi*B and *whi*D like genes have been shown to have a role as transcription factors in mycobacteria^189^. The *whi*E genes detected in the genomes of all of the *Micromonospora* strains, apart from *M. cremea* DSM 45599^T^, are associated with the final stage of sporulation, when polyketide pigments are formed in the spore coat^125^. It is possible that the *whi*E genes may be involved in the formation of black pigments that are produced towards the end of the micromonosporal growth cycle. It is also plausable that over evolutionary time micromonosporae have lost the capacity to form spores on aerial hyphae.

The genomes of most of the *Micromonospora* strains contained a broad range of genes associated with the synthesis of pigments, notably, carotenoids, isorenieratene and sioxanthin. Although the biosynthetic cluster associated with the production of carotenoids was only found in the genome of *M. pallida* DSM 43817^T^, the biocluster for the synthesis of sioxanthin has been associated with the production of a novel glycosylated carotenoid in *Salinispora* strains^190^. This sioxanthin biosynthetic gene cluster was found in all of the *Micromonospora* genomes, apart from those of the type strains of *M. pallida* and *M. inositola*; only the *M. inositola* strain was shown to have the capacity to produce isorenieratene, an aromatic carotenoid produced by green photosynthetic bacteria and a few actinobacteria^191^. All of the *Micromonospora* genomes contained genes implicated in carotenoid biosynthesis, as exemplified by those coding for the production of ß-carotene ketolases, phi-carotenoid synthases and lycopene ß-cyclases^192,193^. The presence of such compounds in non-photosynthetic organisms has been associated with UV protection^194^ and in the case of photosynthetic bacteria with light harvesting complexes^195^. Additional work is needed to account for the presence of genes associated with photosynthesis that were detected in the genomes of strains assigned to groups Id (*M. mirobrigensis* DSM 44830^T^ and *M. siamensis* DSM 45097^T^), II (*M. coxensis* DSM 45161^T^ and *M. halophytica* DSM 43171^T^), IVb (*M. citrea* DSM 43903^T^ and *M. echinofusca* DSM 43913^T^) and IVc (*M. endolithi*ca DSM 44398^T^ and *M. nigra* DSM 43818^T^).

It was particularly interesting that the genome of most, if not all, of the *Micromonospora* strains were replete with genes relevant to their ability to adapt to low levels of carbon^139–141^, temperature fluxes^129,130^, and changes in the osmotic environment^132,133^, a combination of key environmental variables that lend further weight to the suggestion that micromonosporae may be able to colonise multiple microhabitats^45^. In addition, the micromonosporal genomes included genes associated with protection against UV-radiation and for repairing DNA damage. Indeed, all of the strains were found to have the potential to protect and repair damage caused by UV radiation as they have genes associated with the synthesis of Uvr ABCD proteins, excision proteins that have been reported in several bacteria^196^. Further, mutations in uvr ABC genes have been associated with UV sensitivity in *Rhodobacter sphaeroides* ^197^. Genes associated with desiccation were not detected in the genomes of the *Micromonospora* strains though several genes involved in the biosynthesis and uptake of trehalose were seen, trehalose has been linked with tolerance to heat and desiccation in bacteria^198^.

Since the discovery of gentamicin from “M. purpurea” INMI 632 in 1963^71^ hundreds of bioactive molecules with diverse properties and structures have been isolated from *Micromonospora* species^83,199–202^. Major classes of clinically significant specialized metabolites synthesized by micromonosporae include aminoglycosides (gentamicins), anthracyclines (daunorubicin), ansamycins (rifampicins), macrolides (erythromicins), as well as enediyne (calichenomicins) and oligosaccharide (everninomicins) antibiotics. It is not surprising in light of these observations, those drawn from earlier whole genome studies on micromonosporae^85–87^ and from corresponding work on other filamentous actinobacteria^39,203^ that the genomes of the tested strains were rich in biosynthetic gene clusters encoding for known and predicted specialized metabolites, notably antibiotics, siderophores and terpenes. The analysis of the micromonosporal genomes also confirmed the relationship between presence of the aminoshikimate pathway and the capacity of *Micromonospora* strains to synthesize ansamycins; the genomes of all the rifamycin-like producers contained the AHBA synthase-like gene, a key enzyme of this variant of the shikimate pathway implicated in the production of aromatic antibiotics. It is particularly interesting that many of the bioclusters were found only in a few of the micromonosporal genomes, an observation that underlines the merit of selecting representatives of novel actinobacterial taxa in the search for new specialized metabolites^204,205^, thereby providing further evidence that comparative analysis of actinobacterial genomes can be used to select gifted strains for gene mining and natural product discovery^30,206,207^. In contrast, it was not possible to detect any relationship between the phylogeny of *Micromonospora* strains and their source through such an association has been found for *Salinispora* species^208–211^. Indeed, strains assigned to most of the clades and subclades were isolated from diverse geographical regions. It can also be concluded from the analyses of the genomes generated in this study that micromonosporae have a very much greater potential to synthesize specialized metabolites, notably antibiotics, than previously realised. Consequently, *Micromonospora* and other genera classified in the family *Micromonosporaceae* ^45^ should feature much more prominently in the search for new classes of bioactive compounds that are urgently needed to control drug resistant pathogens.

### Revision to descriptions of *Micromonospora* species

#### Emended description of *Micromonospora aurantiaca* Sveshnikova *et al*. 1969

The species description is as given by Sveshnikova *et al*.^212^ with the following changes: The approximate genome size of the type strain is 7.03 Mbp and its genome G+C content is 72.9%.

#### Emended description of *Micromonospora auratinigra* Thawai *et al*. 2004

The species description is as given by Thawai *et al*.^102^ with the following changes: The approximate genome size of the type strain is 6.76 Mbp.

#### Emended description of *Micromonospora carbonacea* Luedemann and Brodsky 1965

The description is as given by Luedemann and Brodsky^96^ with the following changes: The approximate genome size of the type strain is 7.94 Mbp and its genome G+C content is 73.8%.

#### Emended description of *Micromonospora chaiyaphumensis* Jongrungruangchok *et al*. 2008

The species description is as given by Jongrungruangchok *et al*.^104^ with the following changes: The approximate genome size of the type strain is 6.74 Mbp.

#### Emended description of *Micromonospora chalcea* (Foulerton 1905) Ørskov 1923

The species description is as given by Genilloud^44^ with the following changes: The approximate genome size of the type strain is 6.99 Mbp.

#### Emended description of *Micromonospora chersina* Tomita *et al*. 1992

The species description is as given by Tomita *et al*.^103^ with the following changes: The approximate genome size of the type strain is 6.68 Mbp and its genome G+C content is 73.6%.

#### Emended description of *Micromonospora chokoriensis* Ara & Kudo 2007

The species description is as given by Ara and Kudo^111^ with the following changes: The approximate genome size of the type strain is 6.89 Mbp.

#### Emended description of *Micromonospora citrea* Kroppenstedt *et al*. 2005

The species description is as given by Kroppenstedt *et al*.^68^ with the following changes: The approximate genome size of the type strain is 7.21 Mbp and its genome G+C content is 73.8%.

#### Emended description of *Micromonospora coriariae* Trujillo *et al*. 2006

The species description is as given by Trujillo *et al*.^62^ with the following changes: The approximate genome size of the type strain is 6.93 Mbp and its genome G+C content is 71.8%.

#### Emended description of *Micromonospora coxensis* Ara & Kudo 2007

The species description is as given by Ara and Kudo^111^ with the following changes: The approximate genome size of the type strain is 6.77 Mbp.

#### Emended description of *Micromonospora cremea* Carro *et al*. 2012

The species description is as given by Carro *et al*.^97^ with the following changes: The approximate genome size of the type strain is 7.76 Mbp.

#### Emended description of *Micromonospora eburnea* Thawai *et al*. 2005

The species description is as given by Thawai *et al*.^105^ with the following changes: The approximate genome size of the type strain is 7.18 Mbp.

#### Emended description of *Micromonospora echinaurantiaca* Kroppenstedt *et al*. 2005

The species description is as given by Kroppenstedt *et al*.^68^ with the following changes: The approximate genome size of the type strain is 7.20 Mbp and its genome G+C content is 73.2%.

#### Emended description of *Micromonospora echinofusca* Kroppenstedt *et al*. 2005

The species description is as given by Kroppenstedt *et al*.^68^ with the following changes: The approximate genome size of the type strain is 7.00 Mbp and its genome G+C content is 73.3%.

#### Emended description of *Micromonospora echinospora* Luedemann and Brodsky 1964 emend. Kasai *et al*. 2000

The species description is as given by Kasai *et al*.^70^ with the following changes: The approximate genome size of the type strain is 7.78 Mbp and its genome G+C content is 72.3%.

#### Emended description of *Micromonospora endolithica* Hirsch *et al*. 2004

The species description is as given by Hirsch *et al*.^66^ with the following changes: The approximate genome size of the type strain is 7.03 Mbp and its genome G+C content is 72.5%.

#### Emended description of *Micromonospora haikouensis* Xie *et al*. 2012

The species description is as given by Xie *et al*.^112^ with the following changes: The approximate genome size of the type strain is 7.58 Mbp and its genome G+C content is 73.7%.

#### Emended description of *Micromonospora halophytica* Weinstein *et al*. 1968

The species description is as given by Weinstein *et al*.^76^ with the following changes: The approximate genome size of the type strain is 6.27 Mbp and its genome G+C content is 73.0%.

#### Emended description of *Micromonospora inositola* Kawamoto *et al*. 1974

The species description is as given by Kawamoto *et al*.^107^ with the following changes: The approximate genome size of the type strain is 6.71 Mbp and its genome G+C content is 72.2%.

#### Emended description of *Micromonospora inyonensis* Kroppenstedt *et al*. 2005

The species description is as given by Kroppenstedt *et al*.^68^ with the following changes: The approximate genome size of the type strain is 6.92 Mbp and its genome G+C content is 71.9%.

#### Emended description of *Micromonospora krabiensis* Jongrungruangchok *et al*. 2008

The species description is as given by Jongrungruangchok *et al*.^114^ with the following changes: The approximate genome size of the type strain is 7.07 Mbp.

#### Emended description of *Micromonospora marina* Tanasupawat *et al*. 2010

The species description is as given by Tanasupawat *et al*.^95^ with the following changes: The approximate genome size of the type strain is 6.06 Mbp.

#### Emended description of *Micromonospora matsumotoense* (Asano *et al*. 1989) Lee *et al*. 1999

The species description is as given by Lee *et al*.^213^ with the following changes: The approximate genome size of the type strain is 7.75 Mbp and its genome G+C content is 72.3%.

#### Emended description of *Micromonospora mirobrigensis* Trujillo *et al*. 2005

The species description is as given by Trujillo *et al*.^109^ with the following changes: The approximate genome size of the type strain is 6.17 Mbp and its genome G+C content is 73.3%.

#### Emended description of *Micromonospora narathiwatensis* Thawai *et al*. 2008

The species description is as given by Thawai *et al*.^106^ with the following changes: The approximate genome size of the type strain is 6.61 Mbp and its genome G+C content is 72.6%.

#### Emended description of *Micromonospora nigra* (Weinstein *et al*. 1968) Kasai *et al*. 2000

The species description is as given by Kasai *et al*.^70^ with the following changes: The approximate genome size of the type strain is 6.36 Mbp and its genome G+C content is 72.6%.

#### Emended description of *Micromonospora olivasterospora* Kawamoto *et al*. 1983

The species description is as given by Kawamoto *et al*.^113^ with the following changes: The approximate genome size of the type strain is 7.07 Mbp and its genome G+C content is 72.5%.

#### Emended description of *Micromonospora pallida* (Luedemann and Brodsky 1964) Kasai *et al*. 2000

The species description is as given by Kasai *et al*.^70^ with the following changes: The approximate genome size of the type strain is 7.76 Mbp and its genome G+C content is 71.9%.

#### Emended description of *Micromonospora peucetia* Kroppenstedt *et al*. 2005

The species description is as given by Kroppenstedt *et al*.^68^ with the following changes: The approximate genome size of the type strain is 7.37 Mbp and its genome G+C content is 72.3%.

#### Emended description of *Micromonospora purpureochromogenes* (Waksman and Curtis 1916) Luedemann 1971

The species description is as given by Luedemann^214^ with the following changes: The approximate genome size of the type strain is 6.67 Mbp and its genome G+C content is 73.0%.

#### Emended description of *Micromonospora rhizosphaerae* Wang *et al*. 2011

The species description is as given by Wang *et al*.^108^ with the following changes: The approximate genome size of the type strain is 7.18 Mbp.

#### Emended description of *Micromonospora rifamycinica* Huang *et al*. 2008

The species description is as given by Huang *et al*.^215^ with the following changes: The approximate genome size of the type strain is 7.01 Mbp and its genome G+C content is 73.0%.

#### Emended description of *Micromonospora saelicesensis* Trujillo *et al*. 2007

The species description is as given by Trujillo *et al*.^69^ with the following changes: The approximate genome size of the type strain is 7.10 Mbp.

#### Emended description of *Micromonospora sagamiensis* Kroppenstedt *et al*. 2005

The species description is as given by Kroppenstedt *et al*.^68^ with the following changes: The approximate genome size of the type strain is 6.93 Mbp and its genome G+C content is 72.5%.

#### Emended description of *Micromonospora sediminicola* Supong *et al*. 2013

The species description is as given by Supong *et al*.^101^ with the following changes: The approximate genome size of the type strain is 6.89 Mbp and its genome G+C content is 73.6%.

#### Emended description of *Micromonospora siamensis* Thawai *et al*. 2006

The species description is as given by Thawai *et al*.^110^ with the following changes: The approximate genome size of the type strain is 6.25 Mbp.

#### Emended description of *Micromonospora tulbaghiae* Kirby and Meyers 2010

The species description is as given by Kirby and Meyers^59^ with the following changes: The approximate genome size of the type strain is 6.49 Mbp and its genome G+C content is 73.0%.

#### Emended description of *Micromonospora viridifaciens* Kroppenstedt *et al*. 2005

The species description is as given by Kroppenstedt *et al*.^68^ with the following changes: The approximate genome size of the type strain is 7.07 Mbp and its genome G+C content is 72.1%.

#### Emended description of *Micromonospora yangpuensis* Zhang *et al*. 2012

The species description is as given by Zhang *et al*.^56^ with the following changes: The approximate genome size of the type strain is 6.52 Mbp.

#### Emended description of *Micromonospora zamorensis* Carro *et al*. 2012

The species description is as given by Carro *et al*.^97^ with the following changes: The approximate genome size of the type strain is 7.09 Mbp.

## Conclusions

The results of this and corresponding taxonomic analyses based on a comparison of whole genome sequences of bacterial taxa^159,216,217^ are a timely reminder that classification and identification of prokaryotes are markedly data dependent and hence are in a constant state of development due to the introduction of new technologies^3^. To date, much of the emphasis in the GEBA project has been on the analysis of genomic sequences generated from the type strains of diverse taxa in order to expand coverage of the tree of life^35–39^ while other sequence based studies have been focused on many representatives of individual clinically significant bacterial species in order to enhance understanding of pathogenesis^218–220^. It is evident from the present study that the analysis of genome sequences of taxonomically complex genera offers a halfway house between these contrasting approaches to phylogenomics, one which has led to substantial improvements in the classification of the genus *Micromonospora*. In addition, the associated wealth of biological information provides a unique platform for the search and discovery of novel natural products, using genome mining and genetic engineering procedures, while providing leads to unravelling the ecological roles of micromonosporae.

## Methods

### Strains and DNA isolation

To cover the ecologic diversity of micromonosporae, strains of forty *Micromonospora* species (40 type and 2 non-type strains) were obtained from the DSMZ collection (Supplementary Table 8). All of the strains were grown in DSM medium 65 at 28 °C for 7 days when the biomass was harvested. Genomic DNA was extracted from the biomass preparations using a MasterPure™ Gram Positive DNA Purification Kit (Epicentre MGP04100) following the instructions of the manufacturer, albeit with modifications, namely incubation overnight on a shaker with 10 mg proteinase K, 7.5 units achromopeptidase, 7.5 µg/µl lysostaphin, 1050 units lysozyme, and 7.5 units mutanolysin to improve cell lysis.

### Genome sequencing and assembly

The genome sequences of all of the *Micromonospora* strains, apart from the type strain of *M. chalcea*, were generated under the auspices of two “GEBA” projects, namely KMG-II, “From individual species to whole genera” and ACTINO 1000 “Exploiting the genomes of the *Actinobacteria*: plant growth promoters and producers of natural products and energy relevant enzymes united in a taxonomically unresolved phylum”; details on these projects are given in Supplementary Table 9. The genome of *M. chalcea* DSM 43026^T^ was sequenced, trimmed and assembled at Northumbria University using Illumina technology and protocols described by Sangal *et al*.^25^. General aspects related to library construction and sequencing can be found at the JGI website (https://img.jgi.doe.gov/); the number of scaffolds and assembly methods are shown in Supplementary Tables 1 and 9.

### Genome annotation

All of the genomes were annotated through the pipeline developed by the Joint Genome Institute (JGI) at the Department of Energy (DOE) using the Integrated Microbial Genomes Expert Review (IMG-ER) then compared with publicly available genomes of *M. aurantiaca* strains ATCC 27029^T^ and L5 and M*. lupini* Lupac 08. The JGI genome annotation pipeline, which includes Prodigal^221^, was used followed by manual curation using GenePRIMP^222^ for finished genomes and draft genomes. The predicted translation of proteins were analyzed using the National Center for Biotechnology Information (NCBI) non-redundant database, UniProt, TIGRFam, Pfam, PRIAM, KEGG, COG and InterPro databases. RNA gene identification was realised using the tRNAscanSE 21.23^223^ and HMMER 3.0rcl^224^ programs. Prediction of non-coding genes was determine under INFERNAL 1.0.2^225^. The Integrated Microbial Genomes – Expert Review (IMG-ER) platform^226^ permitted additional gene prediction analysis and functional annotation. CRT^227^ and PILER-CR^228^ allowed CRISPR element detection.

### Genome analyses

The core genome was determined through the JGI tool Phylogenetic Profiler for Single Genes using the default options. CRISPRFinder^229^ was used to compare the analysis of CRISPR elements. antiSMASH 3.0^230^ was used to determine and compare biosynthetic gene clusters. Presence of other genes was analyzed through the SEED viewer^128^ after RAST annotation^126,127^ of the genomes.

### Phylogenetic analyses

Genome-scale phylogenies were inferred from whole proteomes using the Genome BLAST Distance Phylogeny (GBDP) method, as previously described^159^. Individual gene trees and multilocus sequence analysis (MLSA) trees were inferred using the phylogenies and gene similarities platform at the GGDC web server^166^ available at https://ggdc.dsmz.de/ phylogeny-service.php through the DSMZ phylogenomics pipeline^163^ adapted to single genes. Multiple sequence alignments were generated using MUSCLE software^231^, maximum-likelihood (ML) and maximum-parsimony (MP) trees were inferred from the alignments with RAxML^232^ and TNT^233^, respectively. For ML, rapid bootstrapping in conjunction with the autoMRE bootstrapping criterion^234^ was followed by a search for the best tree; for MP, 1000 bootstrapping replicates were used in conjunction with tree-bisection-and-reconnection branch swapping and ten random sequence replicates. The sequences were checked for compositional bias using the Χ² test, as implemented in PAUP*^235^. For the MLSA data set, the partition bootstrap (PB) was applied in addition to ordinary bootstrap^236^.

### Phenotypic tests

All of the type strains were examined for a broad range of phenotypic properties known to be of value in *Micromonospora* systematics^44^, namely catalase and oxidase production^62^; degradation of organic compounds^109^; carbon substrate utilization^237^; growth at different temperatures (4, 10, 20, 28, 37 and 45 °C), NaCl concentrations (1, 3, 5, 7 and 9%, w/v) and pH values (4.5, 5.5, 6.5, 8.0 and 9.0) using SA1 agar^109^ as the basal medium; pH values were determined using appropriate buffers, as previously described^97^. Chemotaxonomic properties of the strains were drawn from species descriptions, as indicated in Supplementary Tables 4 and 5.

### Habitat classification

The strains were classified in groups according to the substrates from which they were isolated following the ENVO B classification (https://bioportal.bioontology.org/ontologies/ENVO).

### Statistical analysis

A tip-permutation test in conjunction with the calculation of maximum-parsimony scores was carried out as previously described^236^ to evaluate the phylogenetic conservation of phenotypic and genotypic features of the *Micromonospora* strains with respect to the GBDP tree. To this end, the tips of the tree reduced to the *Micromonospora* clade were permuted 10,000 times (including the original arrangement) and maximum-parsimony scores of the selected characters and each of the trees determined with Tree analysis using New Technology (TNT). The number of times the score of a permuted tree was as low or lower than the score of the original tree yielded the p-value. More sophisticated tests are available for phylogenetic conservation, particularly for continuous characters, but our approach allowed an easy comparison of binary and continuous characters as TNT deals with both. Proportion and count data were brought to the same scale using opm^238^, which generates TNT input files.

A principal coordinate analysis of bootstrap-weighted relative Robinson-Foulds distances between trees was calculated with RAxML. The distances were visualized as principal coordinates, as implemented in R^239^. The Chi-2 test, as implemented in R, was used to determine correlations between binary phenotypic features of the *Micromonospora* strains.

### Data availability

All data generated or analyzed during this study are included in this published article (or in the Supplementary Information files), the genomes are available at the JGI website and with the following accession codes at the NCBI database: *M. aurantiaca* DSM 45487: FMHX01000001-FMHX01000004; *M. auratinigra* DSM 44815^T^: LT594323-LT594323; *M. carbonacea* DSM 43168^T^: FMCT1000001-FMCT01000051; *M. chaiyaphumensis* DSM 45246^T^: FMCS01000001-FMCS01000023; *M. chalcea* DSM 43026^T^: MAGP00000000; *M. chersina* DSM 44151^T^: FMIB01000001-FMIB01000002; *M. chokoriensis* DSM 45160^T^: LT607409-LT607409; *M. citrea* DSM 43903^T^: FMHZ01000001-FMHZ01000002; *M. coriariae* DSM 44875^T^: LT607412-LT607412; *M. coxensis* DSM 45161^T^: LT607753-LT607753; *M. cremea* DSM 45599^T^: FSQT00000000; *M. eburnea* DSM 44814^T^: FMHY01000001-FMHY01000002; *M. echinaurantiaca* DSM 43904^T^: LT607750-LT607750; *M. echinofusca* DSM 43913^T^: LT607733-LT607733; *M. echinospora* DSM 43816^T^: LT607413-LT607413; *M. haikouensis* DSM 45626^T^: FMCW01000001-FMCW01000097; *M. halophytica* DSM 43171^T^: FMDN01000001-FMDN01000064; *M. humi* DSM 45647^T^: FMDM01000001-FMDM01000037; *M. inositola* DSM 43819^T^: LT607754-LT607754; *M. inyonensis* DSM 46123^T^: FMHU01000001-FMHU01000004; *M. krabiensis* DSM 45344^T^: LT598496-LT598496; *M. marina* DSM 45555^T^: FMCV01000001-FMCV01000074; *M. matsumotoense* DSM 44100^T^: FMCU01000001-FMCU01000057; *M. mirobrigensis* DSM 44830^T^: FMCX01000001-FMCX01000022; *M. narathiwatensis* DSM 45248^T^: LT594324-LT594324; *M. nigra* DSM 43818^T^: FMHT01000001-FMHT01000003; *M. pallida* DSM 43817^T^: FMHW01000001-FMHW01000004; *M. peucetia* DSM 43363^T^: FMIC01000001-FMIC01000002; *M. purpureochromogenes* DSM 43821^T^: LT607410-LT607410; *M. rhizosphaerae* DSM 45431^T^: FMHV01000001-FMHV01000003; *M. rifamycinica* DSM 44983^T^: LT607752-LT607752; *M. saelicesensis* DSM 44871^T^: FMCR01000001-FMCR01000011; *M. sediminicola* DSM 45794^T^: FLRH01000001-FLRH01000005; *M. siamensis* DSM 45097^T^: LT607751-LT607751; *M. tulbaghiae* DSM 45142^T^: FMCQ01000001-FMCQ01000019; *M. viridifaciens* DSM 43909^T^: LT607411-LT607411; *M. yangpuensis* DSM 45577^T^: FMIA01000001-FMIA01000002; *M. zamorensis* DSM 45600^T^: LT607755-LT607755.

References for Supplementary Tables 4 and 5 ^240–245^.

## Electronic supplementary material

Supplementary material

Table S2

Table S3
