# Supplementary material for: Genome-based classification of micromonosporae with a focus on their biotechnological and ecological potential
Source: Sci Rep. 2018 Jan 11;8:525. doi: 10.1038/s41598-017-17392-0 (PMC5765111; doi:10.1038/s41598-017-17392-0)
Supplement: Supplementary file 1 — Supplementary material [file 41598_2017_17392_MOESM1_ESM.pdf]

Supplementary Material

Genome-based classification of micromonosporae with a focus on their biotechnological and ecological potential

Lorena Carro[1*], Imen Nouioui[1], Vartul Sangal[2], Jan P. Meier-Kolthoff[3], Martha E. Trujillo[4], Maria del Carmen Montero Calasanz[1], Nevzat Sahin[5], Darren Lee Smith[2], Kristi E. Kim[6], Paul Peluso[6], Shweta Deshpande[7], Tanja Woyke[7], Nicole Shapiro[7], Nikos C. Kyrpides[7], Hans-Peter Klenk[1*], Markus Göker[3] & Michael Goodfellow[1]

[1] School of Biology, Newcastle University, Newcastle upon Tyne, UK.

[2] Department of Biomedical Sciences, Northumbria University, Newcastle upon Tyne, UK.

[3] Leibniz Institute DSMZ – German Collection of Microorganisms and Cell Cultures, Inhoffenstraße 7B, Braunschweig, Germany.

[4] Departamento de Microbiologia y Genetica, Lab 214, Universidad de Salamanca, Salamanca, Spain.

[5] Department of Biology, Faculty of Art and Science, Ondokuz Mayis University, Kurupelit-Samsun, Turkey

[6] Pacific Biosciences, 1380 Willow Rd, Menlo Park, California, USA.

[7] DOE Joint Genome Institute, Walnut Creek, California, USA.

[*] School of Biology, Newcastle University, Newcastle upon Tyne, UK. Lorena Carro: lcg@usal.es Hans-Peter Klenk: hans-peter.klenk@newcastle.ac.uk

Supplementary Figure 1. Abundance and distribution of COG categories found in the genomes of the Micromonospora. The profiles are conserved for most of the strains, with the highest differences observed in the genes that were not assigned to COG categories, the carbohydrate transport and metabolism, and the transcription categories. About half of the genomes presented genes related to cytoskeleton category. Values are given in percentages with respect to the number of genes detected for each genome.

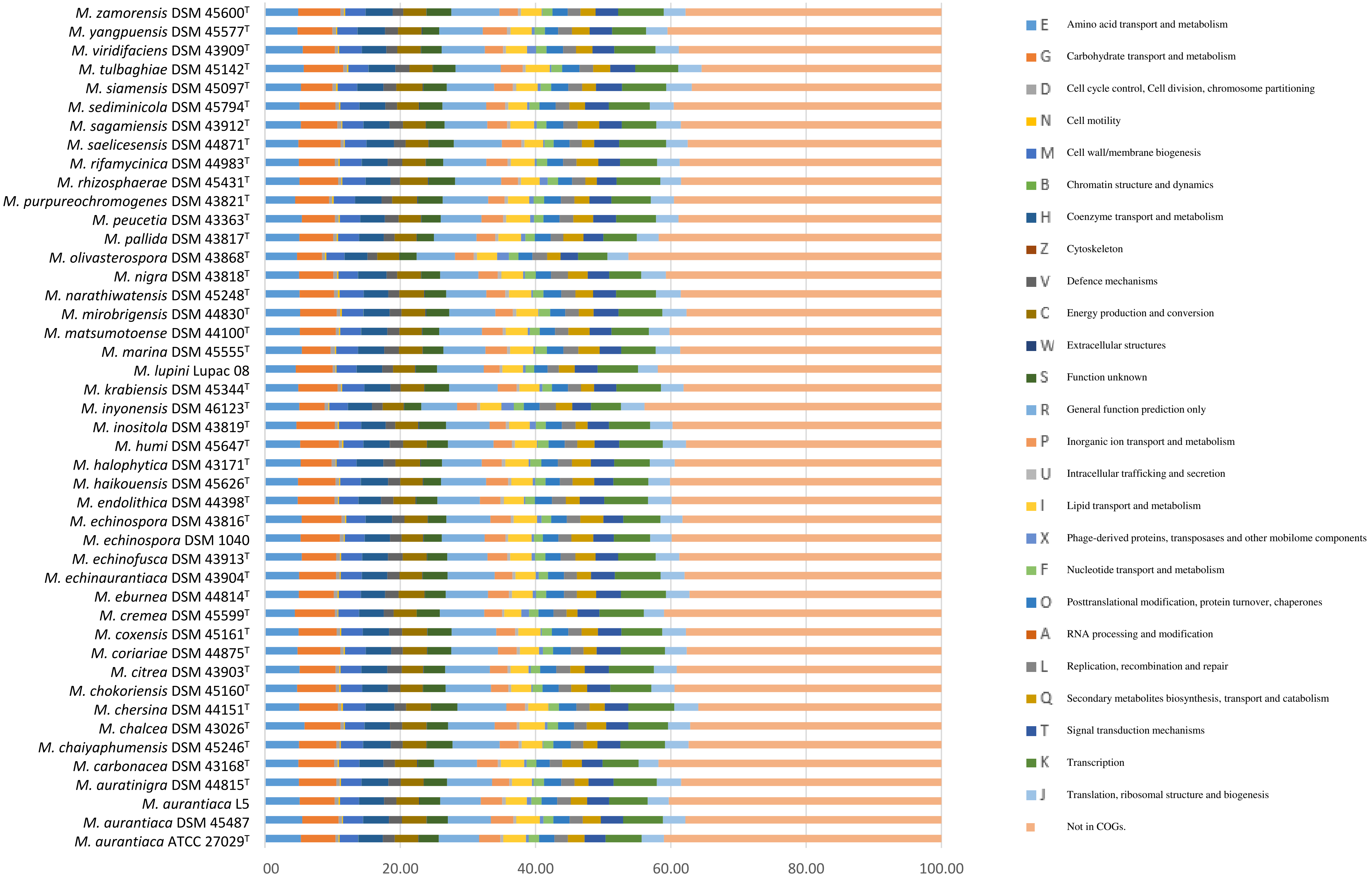

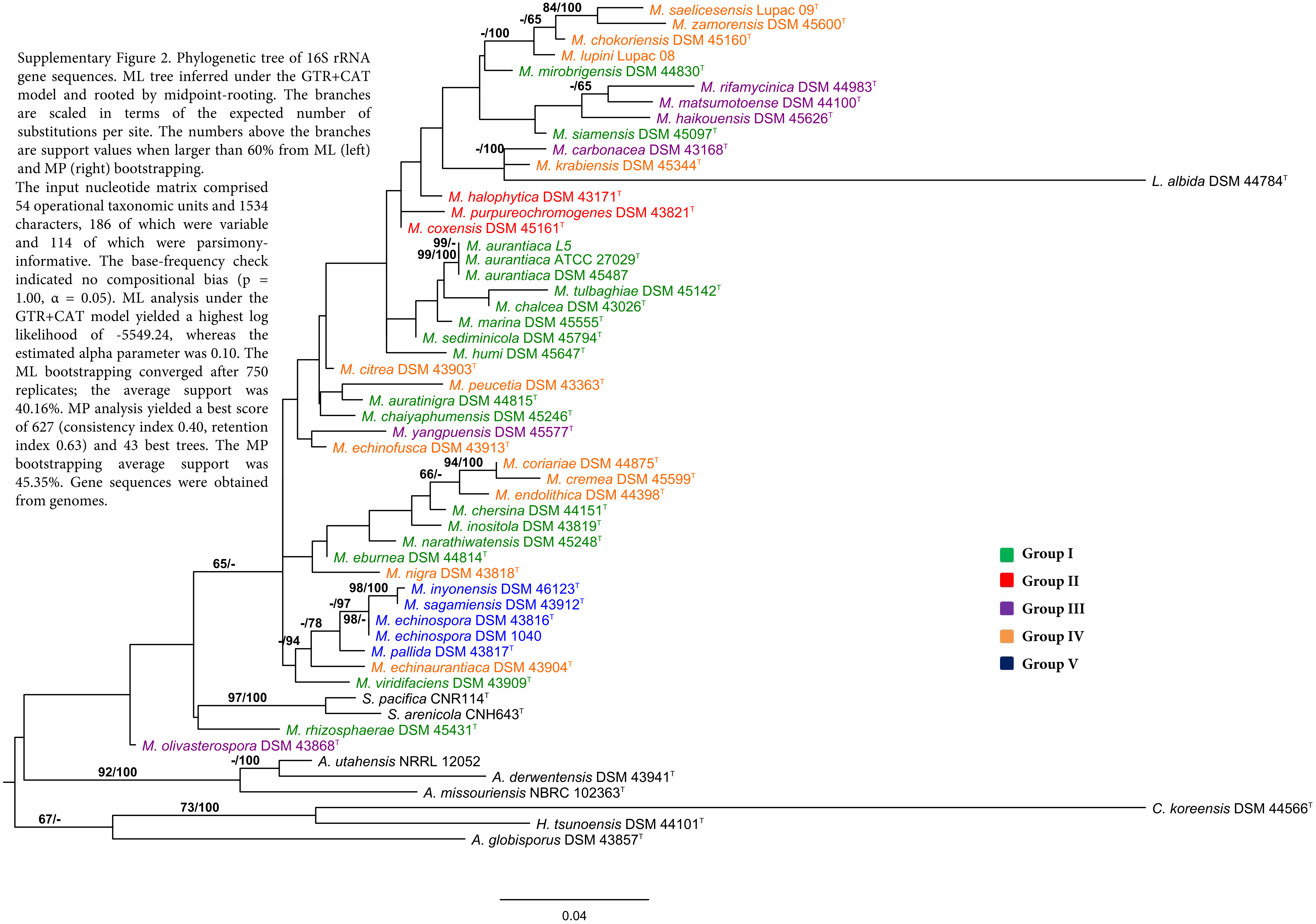

Supplementary Figure 2. Phylogenetic tree of 16S rRNA gene sequences. ML tree inferred under the GTR+CAT model and rooted by midpoint-rooting. The branches are scaled in terms of the expected number of substitutions per site. The numbers above the branches are support values when larger than 60% from ML (left) and MP (right) bootstrapping. The input nucleotide matrix comprised 54 operational taxonomic units and 1534 characters, 186 of which were variable and 114 of which were parsimony-informative. The base-frequency check indicated no compositional bias (p = 1.00, α = 0.05). ML analysis under the GTR+CAT model yielded a highest log likelihood of -5549.24, whereas the estimated alpha parameter was 0.10. The ML bootstrapping converged after 750 replicates; the average support was 40.16%. MP analysis yielded a best score of 627 (consistency index 0.40, retention index 0.63) and 43 best trees. The MP bootstrapping average support was 45.35%. Gene sequences were obtained from genomes.

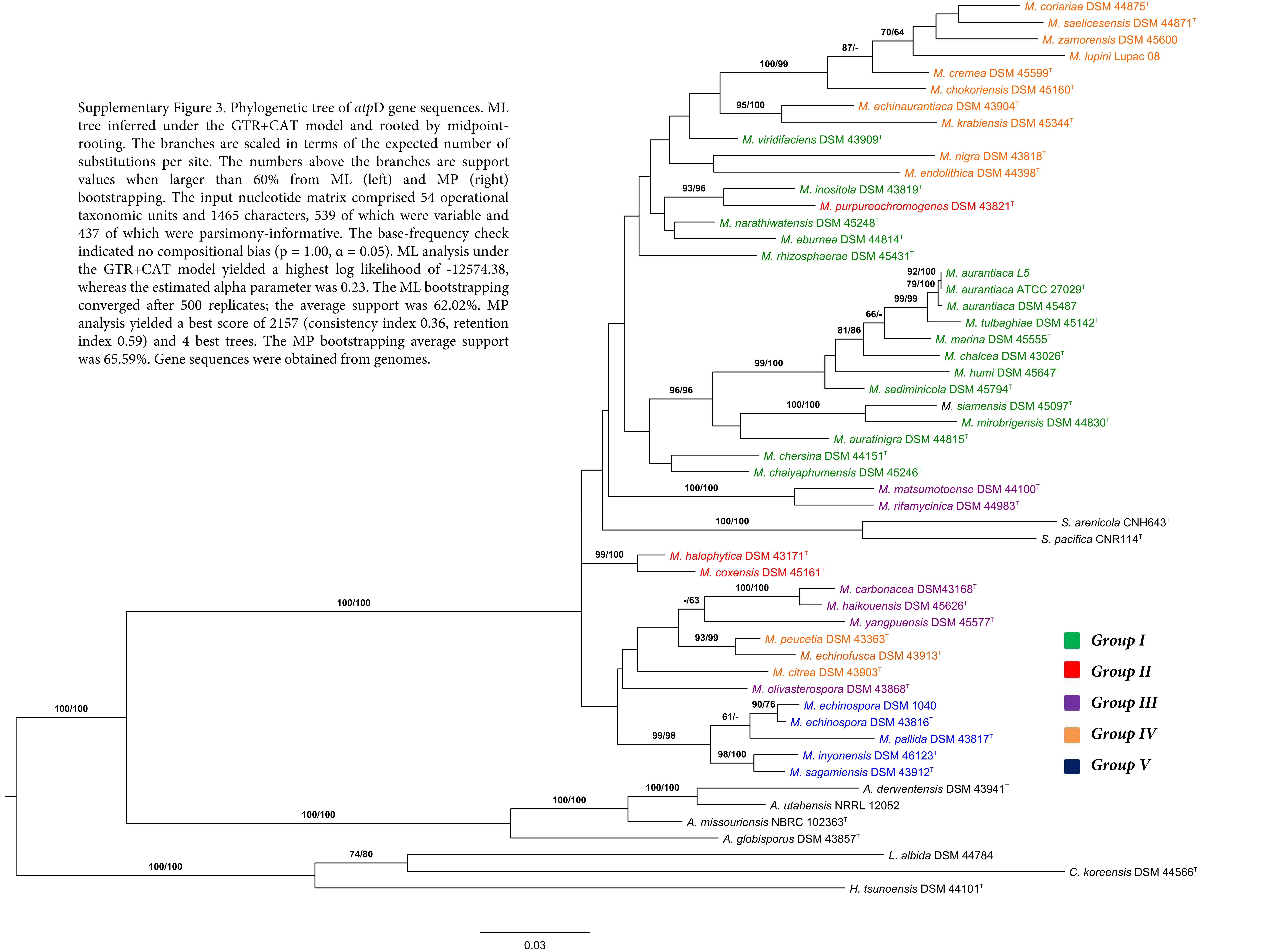

Supplementary Figure 3. Phylogenetic tree of *atp*D gene sequences. ML tree inferred under the GTR+CAT model and rooted by midpoint-rooting. The branches are scaled in terms of the expected number of substitutions per site. The numbers above the branches are support values when larger than 60% from ML (left) and MP (right) bootstrapping. The input nucleotide matrix comprised 54 operational taxonomic units and 1465 characters, 539 of which were variable and 437 of which were parsimony-informative. The base-frequency check indicated no compositional bias ($p = 1.00$, $\alpha = 0.05$). ML analysis under the GTR+CAT model yielded a highest log likelihood of -12574.38, whereas the estimated alpha parameter was 0.23. The ML bootstrapping converged after 500 replicates; the average support was 62.02%. MP analysis yielded a best score of 2157 (consistency index 0.36, retention index 0.59) and 4 best trees. The MP bootstrapping average support was 65.59%. Gene sequences were obtained from genomes.

Supplementary Figure 4. Phylogenetic tree of *gyr*B gene sequences. ML tree inferred under the GTR+CAT model and rooted by midpoint-rooting. The branches are scaled in terms of the expected number of substitutions per site. The numbers above the branches are support values when larger than 60% from ML (left) and MP (right) bootstrapping. The input nucleotide matrix comprised 54 operational taxonomic units and 2058 characters, 926 of which were variable and 662 of which were parsimony-informative. The base-frequency check indicated no compositional bias (p = 1.00, α = 0.05). ML analysis under the GTR+CAT model yielded a highest log likelihood of -20550.26, whereas the estimated alpha parameter was 0.28. The ML bootstrapping converged after 600 replicates; the average support was 67.84%. MP analysis yielded a best score of 3721 (consistency index 0.35, retention index 0.51) and 7 best trees. The MP bootstrapping average support was 68.84%. Gene sequences were obtained from genomes.

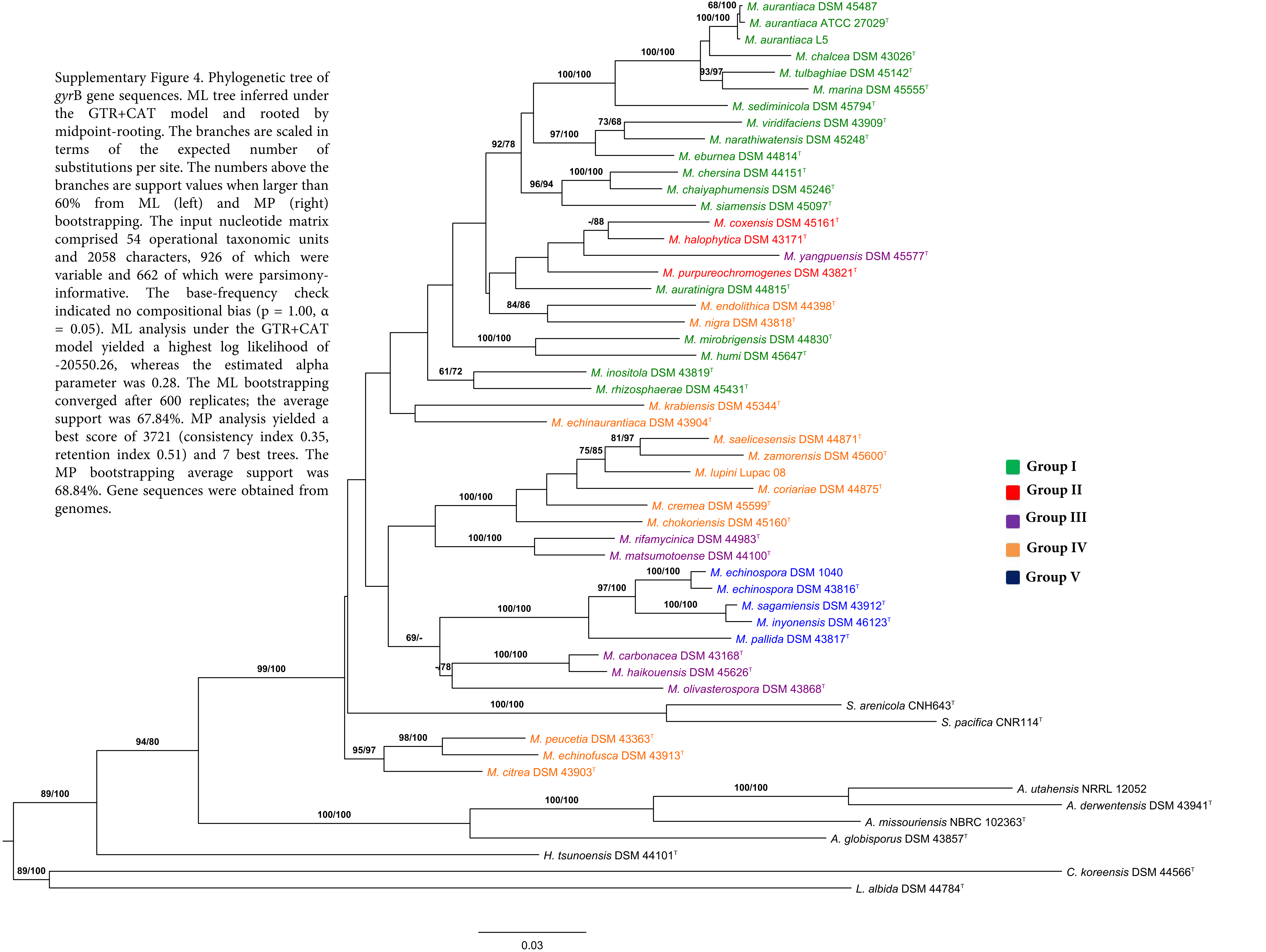

Figure S5. Phylogenetic tree of *recA* gene sequences. ML tree inferred under the GTR +CAT model and rooted by midpoint-rooting. The branches are scaled in terms of the expected number of substitutions per site. The numbers above the branches are support values when larger than 60% from ML (left) and MP (right) bootstrapping. The input nucleotide matrix comprised 54 operational taxonomic units and 2270 characters, 903 of which were variable and 458 of which were parsimony-informative. The base-frequency check indicated no compositional bias (p = 1.00, α = 0.05). ML analysis under the GTR+CAT model yielded a highest log likelihood of -13318.55, whereas the estimated alpha parameter was 0.27. The ML bootstrapping converged after 350 replicates; the average support was 61.61%. MP analysis yielded a best score of 2230 (consistency index 0.54, retention index 0.55) and 80 best trees. The MP bootstrapping average support was 59.33%. Gene sequences were obtained from genomes.

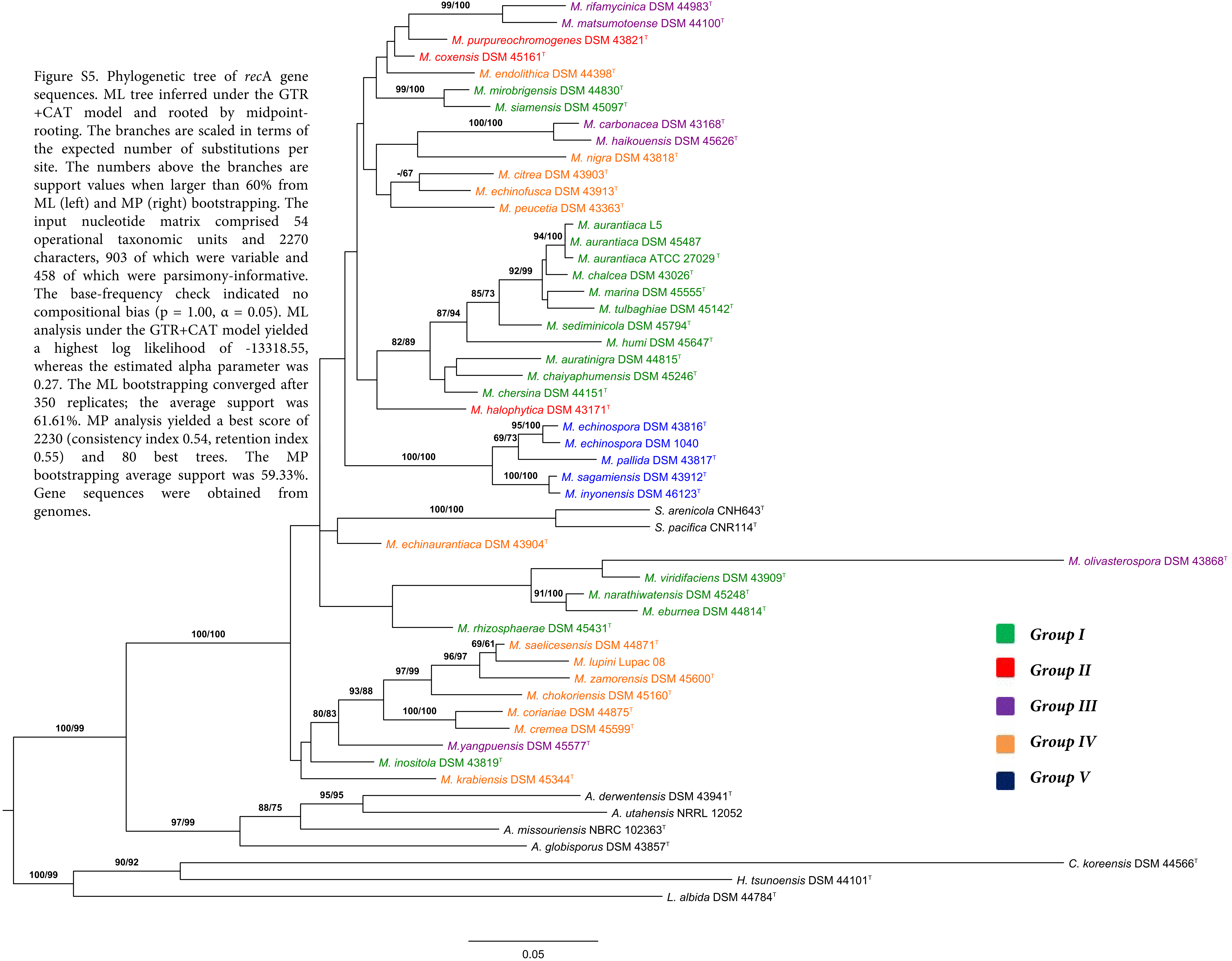

Figure S6. Phylogenetic tree of *rpo*B gene sequences. ML tree inferred under the GTR+CAT model and rooted by midpoint-rooting. The branches are scaled in terms of the expected number of substitutions per site. The numbers above the branches are support values when larger than 60% from ML (left) and MP (right) bootstrapping. The input nucleotide matrix comprised 54 operational taxonomic units and 3558 characters, 1064 of which were variable and 744 of which were parsimony-informative. The base-frequency check indicated no compositional bias (p = 1.00, α = 0.05). ML analysis under the GTR+CAT model yielded a highest log likelihood of -25084.51, whereas the estimated alpha parameter was 0.18. The ML bootstrapping converged after 400 replicates; the average support was 73.61%. MP analysis yielded a best score of 4159 (consistency index 0.35, retention index 0.55) and 8 best trees. The MP bootstrapping average support was 83.90%. Gene sequences were obtained from genomes.

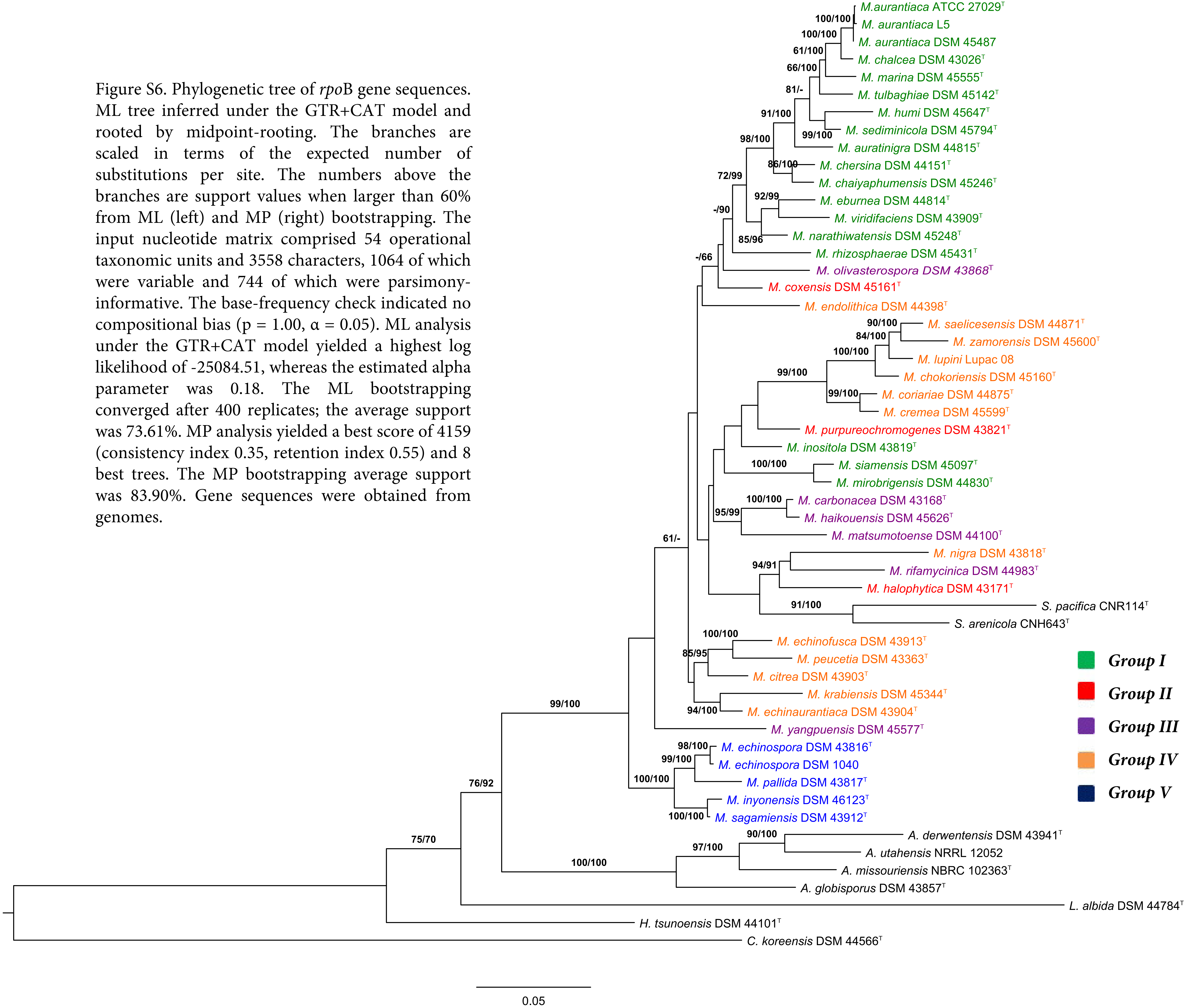

Supplementary Figure 7. MLSA phylogenetic tree of 16S rRNA, *atp*D, *gyr*B, *rpo*B and *rec*A gene sequences. ML tree inferred under the GTR+CAT model and rooted by midpoint-rooting. The branches are scaled in terms of the expected number of substitutions per site. The numbers above the branches are support values when larger than 60% from ML (left) and MP (right) bootstrapping. The input nucleotide matrix comprised 54 operational taxonomic units and 9349 characters, 2992 of which were variable and 2178 of which were parsimony-informative. The base-frequency check indicated no compositional bias ($p = 0.08$, $\alpha = 0.05$). ML analysis under the GTR+CAT model yielded a highest log likelihood of -73463.79, whereas the estimated alpha parameter was 0.19. The ML bootstrapping converged after 300 replicates; the average support was 79.22%. MP analysis yielded a best score of 12420 (consistency index 0.34, retention index 0.52) and 2 best trees. The MP bootstrapping average support was 80.39%. Gene sequences were obtained from genomes.

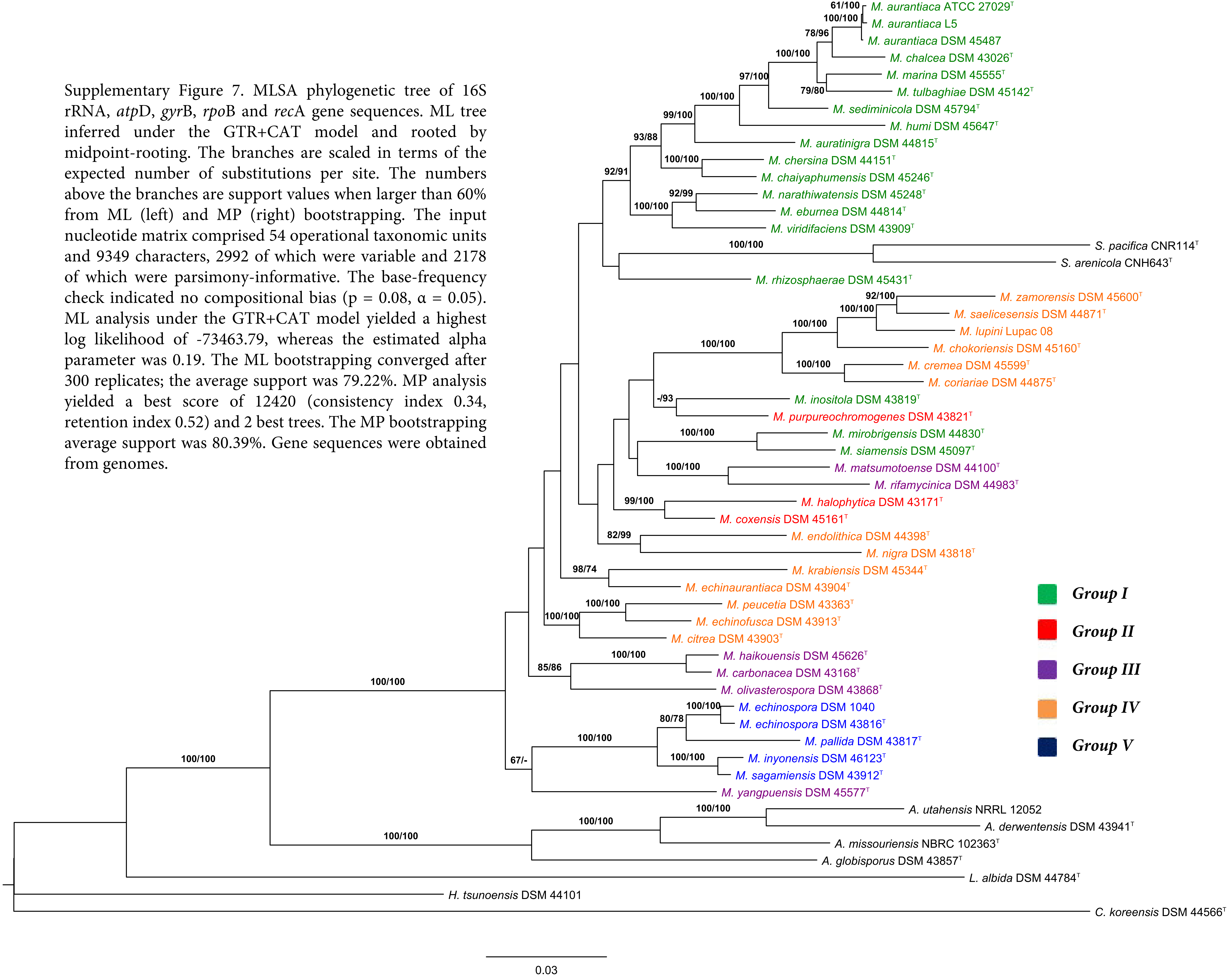

Supplementary Figure 8. Phenotypic profiles of *Micromonospora* strains isolated from different biomes.

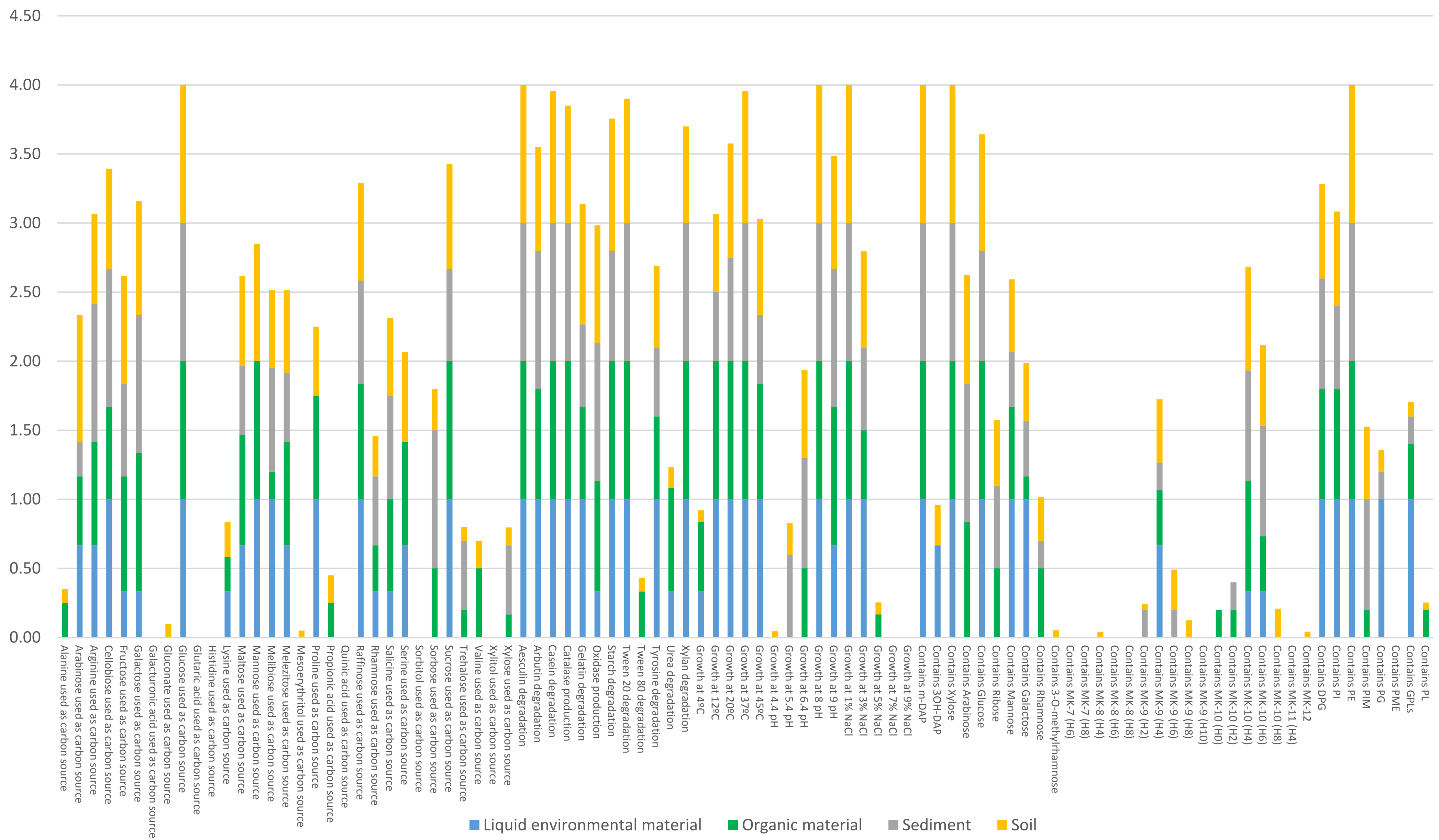

Supplementary Figure 9. Analysis of phenotypic characteristics correlation with each other in *Micromonospora* strains using chi2-test.

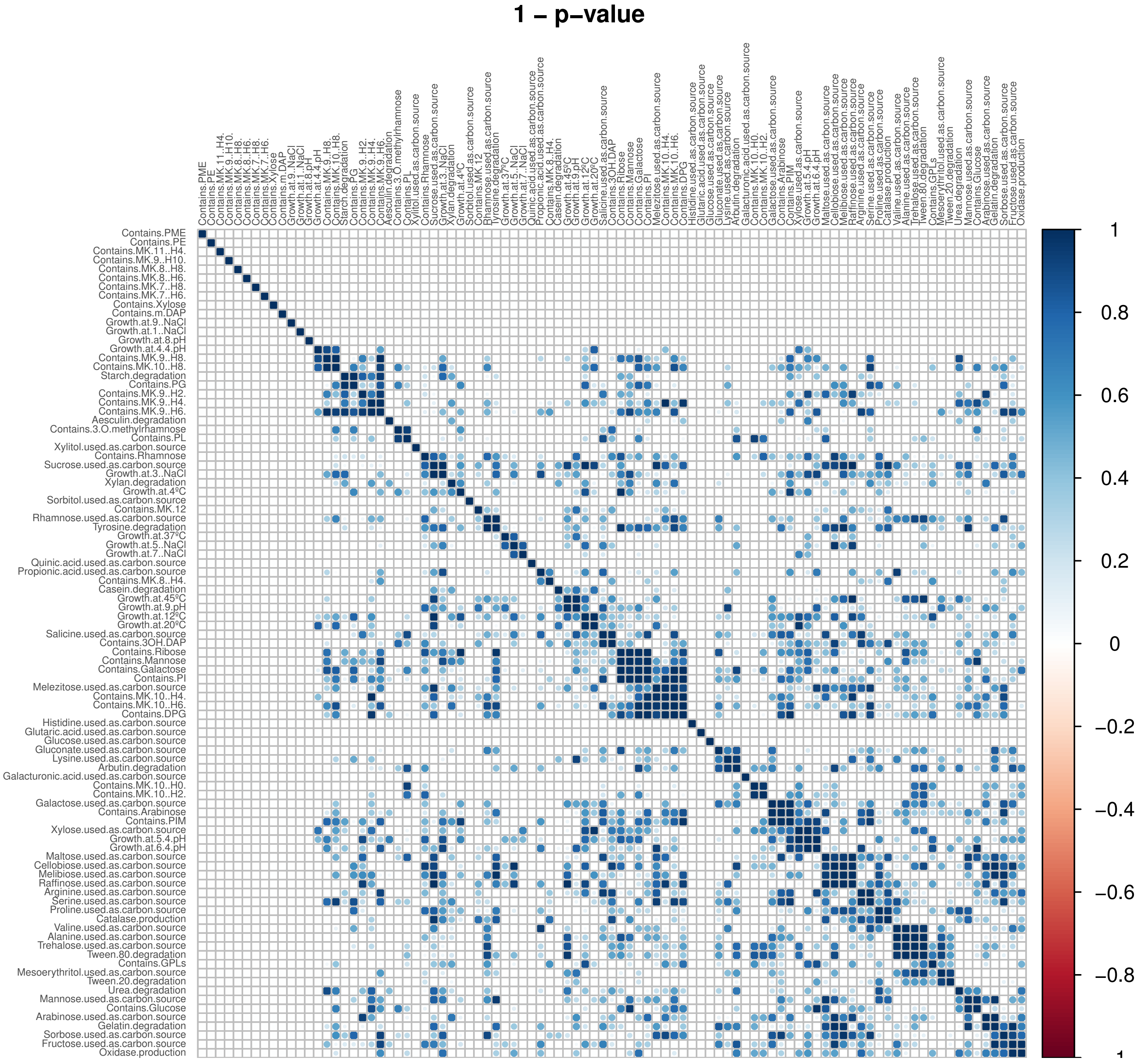

Supplementary Table 1. General features of the genomes of the *Micromonospora* strains.

	Nº bp	Nº Scaffold	% GC	% GC description	Difference	Coding Base	Nº Genes	Nº Pseudogenes	Nº RNA	Nº genes in COGs	Nº genes with Signal Peptide	Biocluster antiSMASH	Nº CRISPR CRISPRFinder
M. aurantiaca ATCC 27029^{T}	7025559	1	72.82	71.6	1.3	6363154	6360	75	63	3543	557	17	6
M. aurantiaca L5	6962533	1	72.84	-	-	6321077	6326	113	64	3728	565	16	5
M. aurantiaca DSM 45487	7372718	4	72.75	-	-	6654902	6770	80	94	3827	415	17	6
M. auratinigra DSM 44815^{T}	6758500	1	73.62	72.8	0.82	6116205	6242	63	97	3653	559	14	9
M. carbonacea DSM 43168^{T}	7941928	51	73.71	73.3	0.41	7078216	7041	0	98	3858	473	36	3
M. chaiyaphumensis DSM 45246^{T}	6743815	23	73.52	72.8	0.72	6060680	6260	0	78	3735	393	15	1
M. chalcea DSM 43026^{T}	6994506	176	72.79	71.9	0.9	6323663	6421	0	91	3833	380	20	2
M. chersina DSM 44151^{T}	6683691	2	73.57	72.9	0.67	6050975	6237	83	76	3809	571	15	2
M. chokoriensis DSM 45160^{T}	6897719	1	71.46	71	0.46	6188562	6306	115	95	3624	421	16	8
M. citrea DSM 43903^{T}	7208999	2	73.82	-	-	6406840	6455	136	104	3736	514	17	3
M. coriariae DSM 44875^{T}	6929687	1	71.76	70.2	1.56	6222426	6519	184	69	3861	563	11	5
M. coxensis DSM 45161^{T}	6769693	1	73.58	73	0.58	6062955	6087	80	102	3608	427	17	4
M. cremea DSM 45599^{T}	7759667	2	71.47	72.4	-0.93	6846654	7388	445	73	4132	438	9	2
M. eburnea DSM 44814^{T}	7185375	2	72.12	71.5	0.62	6451542	6361	154	99	3802	518	20	6
M. echinaurantiaca DSM 43904^{T}	7203133	1	73.2	-	-	6397984	6701	146	74	3955	578	13	2
M. echinofusca DSM 43913^{T}	7002527	1	73.31	-	-	6285801	6159	92	104	3584	511	27	4
M. echinospora DSM 1040	7775486	1	72.31	-	-	6967622	6696	123	108	3915	470	35	7
M. echinospora DSM 43816^{T}	7505732	4	72.53	71.7	0.61	6655274	6487	0	94	3686	442	34	3
M. endolithica DSM 44398^{T}	7026184	2	72.45	70	2.45	6210175	6525	0	87	3720	423	12	5
M. haikouensis DSM 45626^{T}	7581395	97	73.73	71.5	2.23	6775239	6598	0	86	3736	448	41	6
M. halophytica DSM 43171^{T}	6275885	64	72.94	72.5	0.44	5575982	5656	0	114	3249	455	28	3
M. humi DSM 45647^{T}	6662588	37	73.56	73	0.56	6061361	6189	0	133	3653	419	21	9
M. inositola DSM 43819^{T}	6707994	1	72.21	71.4	0.81	5914149	6488	277	76	3715	471	7	0
M. inyonensis DSM 46123^{T}	6923970	4	71.88	-	-	6155926	6259	280	80	3314	437	28	1
M. krabiensis DSM 45344^{T}	7074738	1	72.81	72.1	0.71	6363733	6547	82	98	3845	404	16	3
M. lupini Lupac 08	7321224	4	71.96	-	-	6609068	7104	0	85	3882	677	16	4
M. marina DSM 45555^{T}	6060597	74	72.83	72	0.83	5447200	5550	0	94	3233	261	30	4
M. matsumotoense DSM 44100^{T}	7755577	57	72.27	71	1.27	6807452	6718	0	85	3787	434	48	9
M. mirobrigensis DSM 44830^{T}	6168719	22	73.34	68.6	4.74	5564122	5772	0	77	3418	459	13	1
M. narathiwatensis DSM 45248^{T}	6611957	1	72.59	72	0.59	5960272	5928	63	86	3469	399	19	1
M. nigra DSM 43818^{T}	6363764	3	72.6	71.7	0.9	5718559	5571	117	98	3137	307	23	1
M. olivasterospora DSM 43868^{T}	7071760	2	72.52	71.9	0.62	6139174	6383	0	76	3234	269	22	16
M. pallida DSM 43817^{T}	7762826	4	71.88	71.1	0.78	6933860	6876	181	130	3763	617	31	7
M. peucetia DSM 43363^{T}	7365767	2	72.28	-	-	6549718	6463	164	100	3752	522	25	17
M. purpureochromogenes DSM 43821^{T}	6674134	1	72.98	73	-0.02	5937595	6177	151	86	3540	516	15	4
M. rhizosphaerae DSM 45431^{T}	7181580	3	71.61	70.8	0.81	6344386	6806	290	87	3990	331	7	1
M. rifamycinica DSM 44983^{T}	7011269	1	72.99	71	1.99	6240698	6010	61	95	3489	575	25	8
M. saelicesensis DSM 44871^{T}	7103993	11	71.15	71.6	-0.45	6368434	6653	78	85	3950	674	11	5
M. sagamiensis DSM 43912^{T}	6928726	2	72.51	-	-	6200942	5936	0	107	3460	362	31	11
M. sediminicola DSM 45794^{T}	6885700	5	73.59	74.8	-1.21	6230784	6449	70	106	3702	403	15	5
M. siamensis DSM 45097^{T}	6250119	1	73.2	73	0.2	5636082	5747	66	79	3446	333	15	3
M. tulbaghiae DSM 45142^{T}	6488171	19	72.95	-	-	5914459	6016	46	81	3690	519	16	6
M. viridifaciens DSM 43909^{T}	7073885	1	72.1	-	-	6291433	6527	164	70	3786	551	17	18
M. yangpuensis DSM 45577^{T}	6524299	2	72.02	71.3	0.72	5817148	5960	110	66	3344	328	22	12
M. zamorensis DSM 45600^{T}	7095932	1	71.21	71.8	-0.59	6407193	6521	80	116	3860	454	12	6

Supplementary Table 2. P-values from tip permutation test of *Micromonospora* features respect to phylogenomics.

Table in Supplementary tables file.

Supplementary Table 3. Distribution of genes associated with COG categories in *Micromonospora* strains.

	E	G	D	N	M	B	H	Z	V	C	W	S	R	P	U	I	X	F	O	A	L	Q	T	K	J	-
M. aurantiaca ATCC 27029^T	364	351	31	11	190	2	248	1	119	240	5	201	410	215	30	231	27	105	157	1	135	169	214	366	226	2817
M. aurantiaca L5	378	369	34	10	204	1	262	2	129	250	4	212	437	228	29	239	36	101	165	1	133	183	226	404	228	2598
M. aurantiaca DSM 45487	374	378	41	17	204	1	270	1	132	244	7	222	438	231	38	228	48	110	171	1	144	176	238	416	228	2943
M. auratinigra DSM 44815^T	337	373	34	11	204	1	267	1	122	228	3	230	447	171	27	199	16	104	171	1	131	139	250	430	242	2589
M. carbonacea DSM 43168^T	375	403	39	15	230	2	269	1	128	249	9	182	483	234	36	261	27	110	150	1	140	221	233	404	225	3183
M. chaiyaphumensis DSM 45246^T	339	373	34	11	223	1	275	1	118	237	4	256	472	189	30	208	8	100	176	1	123	140	229	446	236	2525
M. chalcea DSM 43026^T	407	371	35	11	201	2	260	2	124	251	4	217	480	228	28	262	26	109	166	1	128	200	229	408	229	2588
M. chersina DSM 44151^T	342	386	34	16	225	1	288	0	117	248	5	259	489	187	32	202	4	99	177	1	129	152	235	459	240	2428
M. chokoriensis DSM 45160^T	322	390	34	16	213	1	259	0	125	226	3	226	456	175	28	202	13	100	162	1	125	160	232	413	234	2682
M. citrea DSM 43903^T	355	369	39	12	214	1	266	0	148	220	6	220	459	182	31	184	24	95	167	1	142	149	248	462	234	2719
M. coriariae DSM 44875^T	339	447	35	12	217	1	265	0	121	254	5	247	487	197	33	199	41	105	185	1	133	136	251	462	227	2658
M. coxensis DSM 45161^T	326	369	33	11	208	1	256	0	126	243	4	234	432	184	31	215	12	98	160	1	127	159	228	396	230	2479
M. cremea DSM 45599^T	350	469	32	10	243	1	288	0	117	269	0	273	521	209	26	200	91	110	178	1	152	127	257	522	237	3256
M. eburnea DSM 44814^T	343	355	39	12	224	1	246	0	137	263	4	211	430	212	31	211	34	103	181	1	130	195	252	458	238	2559
M. echinaurantiaca DSM 43904^T	364	395	39	11	227	1	270	0	130	244	5	265	503	190	33	216	33	98	172	1	137	150	255	489	256	2746
M. echinofusca DSM 43913^T	361	339	36	12	207	1	252	1	143	237	5	196	396	193	30	210	34	101	151	1	143	198	222	369	232	2575
M. echinospora DSM 1040	369	408	41	10	206	1	263	1	119	223	7	187	445	216	28	244	24	92	161	1	134	227	214	377	223	2801
M. echinospora DSM 43816^T	394	427	39	12	208	1	283	1	132	250	6	195	472	218	32	251	48	104	169	1	143	247	214	400	236	2781
M. endolithica DSM 44398^T	340	382	36	11	202	1	234	0	129	230	8	218	442	214	36	208	14	100	178	1	142	145	255	456	244	2805
M. haikouensis DSM 45626^T	348	396	37	12	216	2	286	1	128	237	6	186	476	231	34	226	27	107	152	1	133	228	202	369	227	2862
M. halophytica DSM 43171^T	322	279	37	8	179	1	244	1	106	224	6	190	359	181	32	210	15	100	150	1	125	171	210	321	226	2407
M. humi DSM 45647^T	349	384	32	13	198	1	262	1	131	236	4	205	453	182	29	220	7	108	161	2	122	171	245	432	231	2536
M. inositola DSM 43819^T	324	395	31	12	229	1	253	0	99	236	10	277	449	168	33	213	63	103	165	1	141	124	221	423	232	2773
M. inyonensis DSM 46123^T	340	251	38	9	184	1	237	0	107	209	6	168	355	197	32	210	123	99	158	1	160	162	186	297	234	2945
M. krabiensis DSM 45344^T	349	412	36	11	231	1	272	0	110	247	5	254	510	199	29	210	31	99	170	1	134	137	234	467	237	2702
M. lupini Lupac 08	349	419	33	10	229	1	292	1	120	250	8	242	530	180	32	234	29	98	153	1	126	180	260	459	224	3222
M. marina DSM 45555^T	327	255	38	11	193	1	233	2	127	210	4	181	373	191	28	205	17	104	147	1	131	188	192	305	218	2317
M. matsumotoense DSM 44100^T	375	388	34	13	225	2	281	0	130	243	6	179	462	223	33	237	23	107	164	1	143	229	236	403	222	2931
M. mirobrigensis DSM 44830^T	324	338	33	14	200	1	241	1	108	221	9	211	426	161	33	203	6	103	139	1	124	137	230	405	223	2354
M. narathiwatensis DSM 45248^T	326	325	36	17	226	1	232	0	104	234	7	201	379	178	36	210	15	99	169	1	132	167	216	377	234	2459
M. nigra DSM 43818^T	301	302	34	12	172	2	251	0	94	209	4	166	338	171	32	192	17	95	136	1	148	174	190	283	218	2434
M. olivasterospora DSM 43868^T	322	251	31	13	183	1	230	0	99	221	5	170	385	189	33	202	119	96	140	1	148	135	176	295	212	3149
M. pallida DSM 43817^T	378	372	39	15	230	1	272	0	120	242	6	184	469	206	35	249	48	103	177	1	137	221	219	368	241	3113
M. peucetia DSM 43363^T	380	341	38	12	210	1	253	0	143	230	6	198	420	223	32	246	40	97	167	1	141	204	237	411	232	2711
M. purpureochromogenes DSM 43821^T	297	335	30	15	209	1	262	0	104	247	9	243	448	161	34	210	43	103	165	1	133	148	216	389	228	2637
M. rhizosphaerae DSM 45431^T	373	418	34	11	252	1	268	0	103	302	8	286	498	182	32	202	87	112	152	1	142	124	213	473	225	2816
M. rifamycinica DSM 44983^T	325	347	35	13	211	2	250	1	138	225	4	165	415	205	32	229	19	102	152	1	129	210	209	357	218	2521
M. saelicesensis DSM 44871^T	356	449	35	10	230	1	292	0	125	244	6	263	512	208	33	200	14	96	170	1	130	131	266	501	230	2703
M. sagamiensis DSM 43912^T	341	346	39	8	201	1	247	0	128	216	4	175	406	188	31	226	25	91	161	1	136	204	215	328	233	2476
M. sediminicola DSM 45794^T	355	367	37	14	196	1	265	1	119	224	7	234	449	195	32	193	24	103	167	1	139	160	244	420	246	2747
M. siamensis DSM 45097^T	331	291	33	14	181	1	240	1	118	243	7	215	436	172	32	197	28	96	158	1	127	127	240	408	235	2301
M. tulbaghiae DSM 45142^T	376	383	37	12	196	2	259	2	133	225	5	217	439	213	29	233	16	104	169	1	131	166	242	417	227	2326
M. viridifaciens DSM 43909^T	394	335	33	11	236	1	256	0	115	245	5	212	451	190	30	220	95	108	173	1	134	172	228	430	243	2741
M. yangpuensis DSM 45577^T	310	333	35	12	194	1	262	1	122	225	8	160	416	231	35	202	27	98	127	1	131	168	215	325	204	2616
M. zamorensis DSM 45600^T	345	440	36	12	212	1	281	0	110	240	4	256	500	192	33	215	7	104	159	1	132	156	235	475	226	2661

E: Amino acid transport and metabolism; G: Carbohydrate transport and metabolism; D: Cell cycle control, cell division, chromosome partitioning; N: Cell motility; M: Cell wall/membrane/envelope biogenesis; B: Chromatin structure and dynamics; H: Coenzyme transport and metabolism; Z: Cytoskeleton; V: Defense mechanisms; C: Energy production and conversion; W: Extracellular structures; S: Function unknown; R: General function prediction only; P: Inorganic ion transport and metabolism; U: Intracellular trafficking, secretion, and vesicular transport; I: Lipid transport and metabolism; X: Mobilome: prophages, transposons; F: Nucleotide transport and metabolism, O: Posttranslational modification, protein turnover, chaperones; A: RNA processing and modification; L: Replication, recombination and repair; Q: Secondary metabolites biosynthesis, transport and catabolism; T: Signal transduction mechanisms; K: Transcription; J: Translation, ribosomal structure and biogenesis; -: Not in COG.

Supplementary Table 4. Fatty acids profiles of the *Micromonospora* type strains.

	M. aurantiaca DSM 43813T [2]	*M. aurantiaca* JCM 10878 [3]	*M. auratinigra* TT111T [4]	*M. auratinigra* TT111T [5]	*M. carbonacea* DSM 43168T [2]	*M. carbonacea* DSM 43168T [6]	*M. carbonacea* DSM 43168T [7]	*M. carbonacea* JCM 3139T [8]	*M. chayaphumensis* NBRC 106529T [4]	*M. chayaphumensis* MC51T [9]	*M. chayaphumensis* MC51T [10]	*M. chalcea* JCM 3031 [3]	*M. chersina* DSM 44151T [11]	*M. chersina* M956-1 [12]	*M. chokoriensis* 219(6)T [13]	*M. citrea* 43903T [14]	*M. coriariae* NAR01T [15]	*M. coxensis* DSM 45161T [2]	*M. cremea* CR30T [16]	*M. eburnea* JCM 12345T [17]	*M. eburnea* LK2-10T [4]	*M. eburnea* LK2-10T [9]	*M. eburnea* 44814T [14]	*M. echinaurantiaca* JCM 3257T [9]	*M. echinaurantiaca* 43904T [14]	*M. echinofusca* 43913T [14]	*M. echinospora* 43816T [14]	*M. endolithica* DSM 44398T [11]	*M. endolithica* DSM 44398T [18]	*M. haikouensis* 232617T [19]	*M. haikouensis* 232617T [7]	*M. haikouensis* DSM 45626T [8]	*M. halophytica* JCM 3125T [8]
C10:0	0	0	0	0	0	0	0	0	0	0.4	0	0	0	0	0	0	0	0	0	0	0	0.3	0	0.4	0	0	0	0	0	0	0	0	0
C11:0	0	0	0	0	0	0	0	0	0	0.4	0	0	0	0	0	0	0	0	0	0	0	0.5	0	0.5	0	0	0	0	0	0	0	0	0
C13:0	0	0	0	0	0	0	0	4.1	0	0.1	0	0	0	0	0	0	0	0	0	0	0	0.3	0	0.6	0	0	0	0	0	0	0	0	0
C14:0	0.61	0	0	0	0.62	0	0	0	0	1	0	0.2	0	0	0	0	0	0	0	0	0.07	0.4	0	0.5	0	0	0	0	0	0	0	0	0
C15:0	3.03	0	0	0.5	0	0.94	0	6.5	0	0	0.7	0	0.34	0	1.39	2.15	0	0	1.48	1.6	0	0	0.31	0	0.61	2.96	3.02	2.79	0.99	6.16	0	6.5	7.8
C16:0	4.3	0.7	5.31	0.7	5.57	1.34	2.3	10.8	1.73	4.8	0.5	0.7	0.45	2	2.98	0.37	0.33	0	1.18	1.1	0.87	0.7	0.41	1.6	0.43	0.7	1.09	1.03	1.27	3.96	2.8	12.3	12.6
C17:0	8.21	7.7	5.5	1.4	1.36	4.93	3.7	15.3	2.56	9.8	3.4	1.8	6.14	5	6.61	4.06	10.3	1.75	5.73	7.9	3.15	4.6	2.29	18.1	4.52	6.14	6.55	0	1.53	14.3	13.2	16.6	10.3
C18:0	4.81	0.5	0	1.5	2.68	0.67	1.1	13.1	0.72	3.6	0.8	0.4	0.78	3	3.71	1.06	0.31	3.55	0.6	2.5	0.61	1.1	0.88	1.4	0.86	0.75	1.55	0.62	0.63	2.99	1.1	11.4	6.1
C19:0	0.58	0.6	1.48	0.3	0	0.12	0.1	0	0	0.4	0.6	0.2	0	0	0	0.86	0.45	0	0	1.4	0.09	0.8	0.29	0	0.85	0.31	0	0	0.19	0	0	0	0
C15:1	0.76	0	0	0	0	0	0	0	0	0	0	0	0	0	0	0	0	0	0	0	0	0	0	0	0	0.41	0	0	0	0	0	0	0
C16:1 2-OH	0	0	0	0.6	0	0	0	0	0	0.5	3.7	0	0	0	0	0	0	4.13	0.47	0	0	1.1	0	0	0	0	0	0	1.23	0	0	0	0
C16:1ω7c	0	0	0	0	0	0	0	0	0	0	0	0	0	0	0	0	0	0	0	0	0	0	0	0	0	0	0	0	1.2	0	0	0	0
C16:1ω9c	1.66	0	0	0	2.95	0.39	0.4	0	0	0	0	0	0	0	0	0	0	0	0	0	0	0	0.12	0	0	0.51	0.52	0	0	0	0	0	0
C17:1	0	0	0	0	0	0	0	0	0	0	0	0	0	0	0	0	0	0	0	0	0	0	0	0	0	0	0	0	0	0	0	0	0
C17:1ω5c	0	0	0	0	0	0	0	0	0	0	0	0	0	0	0	0	0	0	0	0	0	0	0	0	0	0	0	0	0	0	0	0	0
C17:1ω6c	0	0	0	0	0	0	0	0	0	0	0	0	0	0	0	0	0	1.67	0	0	0	0	0	0	0	0	0	0	0	0	0	0	0
C17:1ω8c	0	18.2	10.5	1.5	0	0	0	0	3.21	14.8	4.8	7.5	3.78	0	2.99	0	9	0	5.54	5.2	4.42	2.1	0	8.1	0	0	0	18.4	11.3	0	0	0	0
C17:1ω7c	0	0	0	0	0	0	0	0	0	0	0	0	0	0	0	0	0	4.02	0	0	0	0	0	0	0	0	0	0	0	0	0	0	0
C17:1ω9c	6.08	0	0	0	6.01	10.4	11.2	0	0	0	0	0	0	0	0	7.98	2.33	0	0	0	0	0	2.73	0	9.56	17.6	26	0	0	12.2	10.9	0	0
C17:1ω10c	0	0	0	0	0	0	0	0	0	0	0	0	0	0	0	0	0	0	0	0	0	0	0	0	0	0	0	0	0	0	0	0	0
C18:1ω7c	0	0	0	0	0	0	0	0	0	0	0	0	0	0	0	0	0	0	0.11	0	0.21	0	0	0	0	0	0	0.41	1.05	0	0	0	0
C18:1ω9c	3.16	3	3.5	1.1	14.5	1.16	1.1	0	1.45	6.3	1.4	3.9	0.74	2	1.83	2.7	0.11	3.08	0.53	1.9	1.24	0	0.98	0.6	1.61	2.59	2.41	3.81	9.4	2.68	3.2	0	0
C18:1ω11c	0	0	0	0	0	0.15	0.1	0	0	0	0	0	0	0	0	0	0	0	0	0	0	0	0.19	0	0	0	0.51	0	0	0	0	0	0
C18:1ω12c	0	0	0	0	0	0	0	0	0	0	0	0	0	0	0	0	0	0	0	0	0	0	0	0	0	0	0	0	0	0	0	0	0
C18:3ω6c	0	0	0	0	0	0	0	0	0	0	0.2	0	0	0	0	0	0	0	0	0	0	0	0	0	0	0	0	0	0	0	0	0	0
C19:1	0	0	0	0	0	0.14	0	0	0	0	0	0	0	0	0	4.12	0	0	0	0	0	0	0.75	0	1.29	0.53	0	0	1.11	0	0	0	0
anteiso -C11:0	0	0	0	0	0	0	0	0	0	1.5	0	0	0	0	0	0	0	0	0	0	0	1.2	0	2	0	0	0	0	0	0	0	0	0
iso -C12:0	0	0	0	0.8	0	0	0	0	0	0	0	0	0	0	0	0	0	0	0	0	0	0	0	0	0	0	0	0	0	0	0	0	0
anteiso -C12:0	0	0	0	0	0	0	0	0	0	0	0	0	0.3	0	0	0	0	0	0	0	0	0	0	0	0	0	0	0	0	0	0	0	0
iso -C13:0	0	0	0	0.3	0	0.17	0.2	0	0	0	0.1	0.3	0.65	0	0	0	0.08	0	0.28	0	0	0	0.11	0	0	0.3	0	0	0	0	0	0	0
anteiso -C13:0	0	0	0	0	0	0	0	0	0	0	0	0	0	0	0	0	0	0	0	0	0	0	0	0	0	0	0	0	0	0	0	0	0
iso -C14:0	4.71	0.8	1.43	3.5	0.98	0.44	0.6	0	0.44	0.7	0.3	1.3	2.61	0	0	0.49	0.69	1.36	0.87	0.8	0.28	1	0.29	1.5	0	0.5	0.39	0	0.26	1.56	1.2	0	0
anteiso -C14:0	0	0	0	0	0	0	0	0	0	0	0	0	0	0	0	0	0	0	0	0	0	0	0	0	0	0	0	0	0	0	0	0	0
iso -C15:0	18.4	20	17.5	11.5	11.6	29.9	31.5	22.5	33.9	9.2	31.4	17.5	28.2	21	33	11	24.3	12.3	34	24.1	38.3	31.1	32.7	32.7	27.6	20.8	17.6	27.1	9.44	11.8	14.3	4.4	5.5
anteiso -C15:0	16.4	6.5	1.3	4.6	11.1	9.6	8.7	0	2.11	0.9	2.6	7.3	4.52	5	6.46	1.97	7.15	1.57	9.24	8.5	8.67	5	4.93	4.4	1.85	1.5	3.02	1.58	3	11.7	12.9	0	0
iso -C15:1	0	0	0	1	0	0.13	0	0	0	0	0.2	0.8	0.36	0	0	0	0.33	0	0.4	0	0	0.1	0.47	0.8	0.88	0	0	0.34	0.3	0	0	0	0
iso -C16:0	15.8	20.6	12.5	39.1	19.2	11.8	14.4	0	7.05	31.7	14.6	20.9	26.3	18	12.4	24.3	17.4	0	16.3	17.9	9.33	20.1	11.5	11.2	11.8	19.3	21	6.44	14.7	13.5	15.8	21.7	26.2
anteiso -C16:0	0	0	0	0	0	0	0.7	0	0	0	0	0	0	0	0	0	0	0	0	0	0	0	0	0	0	0	0	0	0	0	0	0	0
iso -C16:1	0.58	0.5	0	9.5	0	0.94	4.7	0	0	0.3	0.4	1.1	3.31	0	0	0.71	0.89	7.94	0.89	0	0	0.4	0.44	0.9	1.44	0.78	0.81	0.63	1.54	0	4.8	0	0
iso -C17:0	3.67	3.6	10.3	4.7	3.24	5.88	0	14.8	22.5	4	11.3	3.9	4.98	10	7.63	5.69	3.34	3.71	4.89	7.7	13.1	8.6	16.5	4.8	11.3	7.73	5.98	7.61	3.56	2.77	0	13.1	16.4
iso -C17:1	0	0	0	0	0	6.34	5.8	0	0	0	0	0	0	4	0	2.5	0	0	0	0	0	0	4.52	0	7.2	6.34	3.41	0	0	0	0	0	0
anteiso -C17:0	5.88	7.1	7.8	5.8	8.9	8.28	7.9	12.4	8.84	3.6	8.3	10.7	5.78	16	5.8	4.57	5.19	8.67	6.03	10.1	10.5	6.2	10.4	3.4	7.22	2.51	3.72	2.64	8.3	8.48	10.1	10.8	13.4
anteiso -C17:1	0	0	0	0	0.76	0.93	0.4	0	0	0	0	0	0	0	0	0	0	1.83	0	0	0	0	0.49	0	0.63	0	0.6	0	0	0	0	0	0
iso -C17:1ω6c	0	0	0	0	0	0	0	0	0	0	0	0	0	0	0	0	0	0	0	0	0	0	0	0	0	0	0	0	0	0	0	0	0
iso -C17:1ω8c	0	0	0	0	0	0	0	0	0	0	0	0	0	0	0	0	0	0	0	0	0	0	0	0	0	0	0	0	0	0	0	0	0
iso -C17:1ω9c	0	4.4	16.1	2.7	0	0	0	0	11	0	5.6	9.2	7.94	0	4.55	0	0	12.6	4.82	1.6	4.26	0	0	0	0	0	0	11.3	9.99	0	0	0	0
anteiso -C17:1ω9c	0	0.5	0	1.4	0	0	0	0	0.62	0.1	0.4	1.8	1.73	0	1.83	0	0	0	0.86	0	0.75	0.5	0	0	0	0	0	0.32	1.5	0	0	0	0
iso -C17:0 3-OH	0	0	0	0	0	0	0	0	0	0	0	0	0	0	0	0	0	0	0	0	0	0	0	0	0	0	0	0	0	0	0	0	0
iso -C18:0	0	0	0	1.9	0	0	0	0	0	0.5	0.4	0.4	0.73	1	0	0.78	0	2.53	0.21	0.7	0	0.8	0.45	0.2	0	0	0.8	0	0	0	0	0	0
anteiso -C18:0	0	0	0	0	0	0	0	0	0	0	0	0	0	0	0	0	0	0	0	0	0	0	0	0	0	0	0	0	0	0	0	0	0
iso -C18:1ω7c	0	0	0	0	0	0	0	0	0	0	0	0	0	0	0	0	0	0	0	0	0	0	0	0	0	0	0	0	0	0	0	0	0
iso -C18:1	0	0	0	0	0	0	0	0	0	0	0.2	0	0	0	0	0	0	0	0	0	0	0	0	0	0	0	0	0	0.18	0	0	0	0
iso -C19:0	0	0	0	0	0	0	0	0	0	0	0	0	0	0	0	0	0	0	0	0	0	0	0	0	0	0	0	0	0	0	0	0	0
anteiso -C19:0	0	0	0	0	0	0	0	0	0	0	0	0	0	0	0	0	0	0	0	0	0	0	0	0	0	0	0	0	0	0	0	0	0
9-Methyl C16:1	1.37	0	0	0	4.1	0	0	0	0	0	0	0	0	0	0	0	0	0	0	0	0	0	0	0	0	0	0	0	0	0	0	0	0
10-Methyl C16:0	0	0	0	0	0	0	0	0	0	0	0	0	0	2	0	0	0	0	0	0	0	0	0	0	0	0	0	0	0	0	0	0	0
10-Methyl C17:0	0	2.7	2.42	2.4	2.71	4.57	4.2	0	1.58	0.6	5.8	4.6	2.32	4	4.36	20.2	15.9	21.6	4.33	4.2	2.18	8.2	5.21	2.7	9.05	6.16	2.82	5.54	9.15	6.25	7.6	0	0
10-Methyl C18:0 (TBA)	0	0	0	0.9	2.73	0.83	0.9	0	0.6	0.1	1.6	0	0.54	4	1.85	3.08	0.26	2.13	0.39	0.9	0.45	1.2	2	0.1	1.37	1.63	0	1.12	7.76	1.62	2.1	0	0
10-Methyl C19:0	0	0	0	0	0	0	0	0	0	0	0.2	0	0	0	0	0	0	0	0	0	0	0	0	0	0	0	0	0	0.47	0	0	0	0
Summed features 3	0	0	1.45	0.3	0	0	0	0	1.1	2.1	0.1	0	0.3	0	0	0	0	0	0.28	0	0.47	0.1	0	0.3	0	0	0	0.78	0	0	0	0	0
Summed features 6	0	0	2.95	0.1	0	0	0	0	0.6	0.2	0.8	0	0.4	0	0	0	0	0	0.22	0	0.28	0.3	0	0.1	0	0	0	0.93	0	0	0	0	0

	M. humi P0402T [20]	*M. inositola* 43819T [14]	*M. inyonensis* 46123T [14]	*M. krabiensis* MA-2T [7]	*M. lupini* Lupac 14NT [21]	*M. marina* DSM 45555T [2]	*M. marina* JSM11 [3]	*M. matsumotoense* IMSNU 22003T [19]	*M. mirobrigensis* WA201T [6]	*M. narathiwatensis* BTG4-1T [17]	*M. nigra* NRRL 3097T [4]	*M. nigra* 43818T [14]	*M. olivasterospora* JCM 7348T [23]	*M. olivasterospora* JCM 7348T [24]	*M. olivasterospora* NRRL 8178T [4]	*M. pallida* JCM 3133T [9]	*M. peucetia* 43363T [14]	*M. purpureochromogenes* DSM 43821T [2]	*M. rhizosphaerae* 211018T [23]	*M. rifamycinica* AM105T [19]	*M. saelicesensis* Lupac 09T [21]	*M. sagamiensis* JCM 3310T [9]	*M. sagamiensis* 43912T [14]	*M. sediminicola* SH213 [3]	*M. siamensis* TT2-4T [19]	*M. tulbaghiae* TVU1T [2]	*M. viridifaciens* NBRC 101887T [4]	*M. viridifaciens* 43909T [14]	*M. yangpuensis* JCM 18319T [9]	*M. zamorensis* CR38T [16]
C10:0	0	0	0	0	0	0	0	0	0	0	0	0	0	0	0	0.5	0	0	0	0	0	0.4	0	0	0	0	0	0	0.4	0
C11:0	0	0	0	0	0	0	0	0	0	0	0	0	0	0	0	0.7	0	0	0	0	0	0.5	0	0	0	0	0	0	0.4	0
C13:0	0	0	0	0	0	0	0	0	0	0	0	0	0.19	0	0	0	0	0	0.03	0	0	0	0	0	0	0	0	0	0.1	0
C14:0	0	0	0	0	0	0	1.1	1.46	0	0	0	0.43	0.27	0.3	0.24	0.4	0	0	0.21	0.58	0.03	0.9	0	0.2	0	0	0.12	0	1	0
C15:0	0	1.9	0.19	0	0	0.8	0	1.55	1.1	2	0	1.47	0.68	0.4	0	0	2.11	1.58	2.55	1.33	0	0	0	0	3.24	1.65	0	1.21	0	1.76
C16:0	0	0.72	0.69	0	0.23	0.9	5.1	6.06	0	1.4	16.5	4.54	0.9	1.9	0.53	4.3	1.21	0.98	0	3.98	0.53	2.7	0.72	1.1	2.49	3.03	0.58	0.48	4.8	0.51
C17:0	13.6	10.7	0.85	3.9	0.5	4.9	6.4	5.54	3.33	8.3	7.44	4.84	1.93	0.8	0	1.2	4.49	7.8	1.03	4.84	4.55	2.8	0.84	4.7	8.27	5.99	2.19	6.87	9.8	5.45
C18:0	0	2.04	0.36	0	0.2	1.4	2.5	7.23	0.54	1.7	4.06	4.29	1.25	1.9	0.57	2.6	2.03	2.12	0	5.9	0.5	4.9	0.98	1.1	1.72	1.76	0.74	0.78	3.6	0.37
C19:0	0	2.58	0	0	0	0.4	0.4	0.72	0.58	0.5	0	0.76	0	0	0	0	0.5	0.67	0.26	0.7	0.28	0.4	0	0.5	0.26	0	0.28	0.57	0.4	0.14
C15:1	0	0	0	0	0	0	0	0	0.11	0	0	0	0	0	0	0	0.38	0	0	0	0	0	0	0	0	0	0	0	0	0
C16:1 2-OH	0	0	0	0	0	0	0	0	0	0.9	0	0	0	1	2.58	0	0	0	0	0	0	0	0	0	0	0	0	0	0.5	0
C16:1ω7c	0	0	0	0	0	0	0	0	0	0	0	0	0	0	0	0	0	0	0	0	0	0	0	0	0	0	0	0	0	0
C16:1ω9c	0	0	2.18	0	0	0.9	0	1.89	0.15	0	0	0.88	1.34	0	0	0	0.75	0	0.7	1.4	0	0	1.24	0	0	0	0	0	0	0
C17:1	0	0	0	0	0	0	0	0	0	0	0	0	0	0	0	0	0	0	0	0	0	0	0	0	0	0	0	0	0	0
C17:1ω5c	0	0	0	0	0	0	0	0	0	0	0	0	0	0	0.38	0	0	0	0	0	0	0.2	0	0	0	0	0	0	0	0
C17:1ω6c	0	0	0	0	0	0	0	0	0	0	0	0	0.6	0	0	0	0	0	0.17	0	0	0	0	0	0	0	0	0	0	0
C17:1ω8c	12.5	0	0	5.4	5.23	0	12.6	0	0	3.3	9.27	0	0	1	2.72	1.6	0	0	0	0	16.2	4.8	0	8.9	0	0	8.26	0	14.8	18.9
C17:1ω7c	0	0	0	0	0	12.7	0	0	0	0	0	0	3.13	0	0	0	0	0	12.8	0	0	0	0	0	0	0	0	0	0	0
C17:1ω9c	0	7.24	8.96	0	0	6.4	0	7.94	5.2	0	0	6.38	0	0	0	0	14.1	6.47	0	5.58	0	0	8.13	0	2.37	1.74	0	3.54	0	0
C17:1ω10c	0	0	0	0	0	0	0	0	0	0	0	0	0	0	0	0	0	0	0	0	0	0	0	0	8.09	0	0	0	0	0
C18:1ω7c	0	0	0	0	0.15	0	0	0	0	0	2.73	0	0.35	0	0	0	0	0	0.12	0	0.25	0	0	0	0	0	0.13	0	0	0
C18:1ω9c	0	1.47	6.71	0	1.29	4.5	10.4	14	0.78	0.6	38.6	20.2	1.74	0.7	0.92	1.8	2.85	1.58	0.52	14.9	2.26	4.4	8.39	2.7	1.36	0	2	0.55	6.3	0.89
C18:1ω11c	0	0	0	0	0	0	0	0	0.17	0	0	0.85	0	0	0	0	1.53	0	0	0	0	0	0	0	0	0	0	0	0	0
C18:1ω12c	0	0	0	0	0	0	0	0	0	0	0	0	0	0	0	0	0	0	0	0	0	0	0	0	0	0	0	0	0	0
C18:3ω6c	0	0	0	0	0	0	0	0	0	0	0	0	0	0	0	0	0	0	0	0	0	0	0	0	0	0	0	0	0	0
C19:1	0	1.17	0	0	0	0	0	0	1.42	0	0	1.41	0	0	0	0	0.71	0	0	0	0	0	0	0	0	0	0	0	0	0
anteiso -C11:0	0	0	0	0	0	0	0	0	0	0	0	0	0	0	0	1.3	0	0	0	0	0	1.7	0	0	0	0	0	0	1.5	0
iso -C12:0	0	0	0	0	0	0	0	0	0	0	0	0	0	0	0	0	0	0	0	0	0	0	0	0	0	0	0	0	0	0
anteiso -C12:0	0	0	0	0	0	0	0	0	0	0	0	0	0	0	0	0	0	0	0	0	0	0	0	0	0	0	0	0	0	0
iso -C13:0	0	0	0.13	0	0.08	0	0	0	0	0	0	0	0	0.1	0.13	0	0	0	0	0	0.13	0	0	0.3	0	0	0.12	0.59	0	0.28
anteiso -C13:0	0	0	0	0	0	0	0	0	0	0	0	0	0	0	0	0	0	0	0	0	0	0	0	0	0	0	0	0	0	0
iso -C14:0	0	0.5	0.25	1.7	0.95	0.7	0.7	2.29	0.33	0	2.78	0.61	2.54	4	0.67	0.4	1	1.14	1.96	0.93	0.2	0.5	0	0.7	2.47	6.17	0.14	0	0.7	0.24
anteiso -C14:0	0	0	0	0	0	0	0	0	0	0	0	0	0	0	0	0	0	0	0	0	0	0	0	0	0.2	0	0	0	0	0
iso -C15:0	12.7	13.9	17.8	35.7	17.9	25.1	19.4	9.1	17.5	39.6	3.09	5.52	21.7	20.2	32.6	50.8	11	19.3	27.5	10.7	32	29.3	17	27.7	20.7	21.8	37.8	30.7	9.2	31.2
anteiso -C15:0	10.6	2.58	0.56	13.7	4.41	4.9	5.9	11.8	4.22	6.4	1.14	2	4.34	6.5	1.17	2.7	0	3.12	6.1	8.57	4.76	1.4	0	6.2	5.73	14	5.89	4.19	0.9	10.5
iso -C15:1	0	0	1.12	0	0	0.3	0.1	0.71	0	0	0	0.33	0	0	0	0.1	0.56	1.14	0	0	0.15	0	0	0.5	0	0	0	0	0	0
iso -C16:0	22.6	19	18	12.1	34.1	16.4	16.5	12.1	15.2	9.6	2.7	19.7	30.9	36	25.5	5.7	35.4	26.5	12.6	12.9	5.25	12.9	16.1	14.3	30.7	34	4.85	4.13	31.7	4.87
anteiso -C16:0	0	0	0	0.8	0	0	0	0	0	0	0	0	0	0	0	0	0	0	0	0	0	0	0	0	0	0	0	0	0	0
iso -C16:1	0	0.97	5.97	5.2	6.45	0.6	0.5	5.13	0.7	0	0	1.34	0	1.3	0	0.2	2.72	3.41	0	2.48	0.28	0.5	0	1	0	1.54	0	0.54	0.3	0.19
iso -C17:0	0	8.47	6.49	0	2.49	7.9	4.8	1.62	8.2	10.8	1.24	3.65	6.64	6.4	9.47	12.4	3.91	7.54	4.06	8.13	7.45	14	15.9	7.6	3.44	2.36	10.9	8.94	4	5.15
iso -C17:1	0	7.39	21.1	0	0	0	0	0	16.2	0	0	6.08	0	0	0	0	4.01	0	0	0	0	0	24.1	0	0	0	0	3.6	0	0
anteiso -C17:0	7.47	6.39	3.25	6.8	6.27	6.9	5.2	3.62	13.4	7.2	1.49	6.85	5.9	7.6	3.85	4.4	2.17	4.69	4.03	7.93	5.8	5.4	2.64	6.9	5.31	5.93	11.9	9.47	3.6	8.02
anteiso -C17:1	0	0.75	1.93	0	0	0.5	0	1.56	2.39	0	0	0.74	0	0.6	0	0	0	1.02	0	1.56	0	0	0	0	0	0	0	0.86	0	0
iso -C17:1ω6c	0	0	0	0	0	0	0	0	0	0	0	0	0	0	0	0	0	0	0	0	0	0	0	0	0	0	0	0	0	0
iso -C17:1ω8c	0	0	0	4.7	0	0	0	0	0	0	0	0	0	0	0	0	0	0	0	0	0	0	0	0	0	0	0	0	0	0
iso -C17:1ω9c	0	0	0	0	9.82	0	4.7	0	0	2.5	1.04	0	0	0	8.95	0	0	0	0	0	10	0	0	12.8	0	0	8.61	0	0	3.94
anteiso -C17:1ω9c	0	0	0	0	2.91	0	0.4	0	0	0	0	0	0	0.6	0.27	0.2	0	0	0	0	0.74	0.5	0	1.1	0	0	2.26	0	0.1	0.73
iso -C17:0 3-OH	0	0	0	0	0	0	0	0	0	0	0	0	0	0.4	0	0	0	0	0	0	0	0	0	0	0	0	0	0	0	0
iso -C18:0	0	0.66	0.2	0	0.1	0.3	0.3	0	0	0	0	0.43	1.34	1	0.89	0.1	0.62	0.65	0.41	0	0	0.5	0	0.3	0	0	0	0	0.5	0
anteiso -C18:0	0	0	0	0	0	0	0	0	0	0	0	0	0	0	0	0	0	0	0	0	0	0	0	0	0	0	0	0	0	0
iso -C18:1ω7c	0	0	0	0	0	0	0	0	0	0	0	0	0	0	0	0	0	0	0	0	0	0	0	0	0	0	0	0	0	0
iso -C18:1	0	0	0	0	0	1.97	0	0	0	0	0	0.37	0	0	0	0	0	0	0	0.6	0	0	0	0	0	0	0	0	0	0
iso -C19:0	0	0	0	0	0	0	0	0	0	0	0	0	0	0	0	0	0	0	0	0	0	0	0	0	0	0	0	0	0	0
anteiso -C19:0	0	0	0	0	0	0	0	0	0	0	0	0	0	0	0	0	0	0	0	0	0	0	0	0	0	0	0	0	0	0
9-Methyl C16:1	0	0	0	0	0	0	0	1.24	0	0	0	0	0	0	0	0	0	9.14	0	2.67	0	0	0	0	0	0	0	0	0	0
10-Methyl C16:0	0	0	0	0	0	0	0	0	0	0	0	0	1.11	3	0	0	0	0	0.62	0	0	0	0	0	0	0	0	0	0	0
10-Methyl C17:0	0	7.55	2.2	8.9	5.28	1.2	0	1.15	5.24	2.9	0	4.01	2.05	0.9	4.21	0.3	6.79	1.17	14.2	1.8	6.42	0.3	2.04	0	3.03	0	0.7	18	0.6	5.39
10-Methyl C18:0 (TBA)	0	1.53	0.99	1.1	0.43	0.4	0	2.07	0.65	0.5	0	2.36	0.87	1.3	1.36	0.2	1.18	0	0.09	1.55	0.92	0	0	0	0.62	0	0.17	1.71	0.1	1.16
10-Methyl C19:0	0	0	0	0	0	0	0	0	0	0	0	0	0	0	0	0	0	0	0	0	0	0	0	0	0	0	0	0	0	0
Summed features 3	0	0	0	0	0.4	0	0	0	0	0	3.36	0	2.71	0	1.13	2.7	0	0	0.71	0	0.4	1.7	0	0	0	0	1.33	0	2.1	0.52
Summed features 6	0	0	0	0	0	0.4	0	0	0	0	2.09	0	0	0	0.74	0	0	0	0	0	0.65	0	0	0	0	0	0.35	0	0.2	0.17

Summed features 3 (C16:1ω7c / iso-C15:0 2OH); Summed features 6 (C19:1ω11c/C19:1ω9c)

[] Reference equivalence in supplementary reference list.

Supplementary Table 5. Phenotypic characteristics of *Micromonospora* type strains.

	Alanine used as carbon source	Arabinose used as carbon source	Arginine used as carbon source	Cellobiose used as carbon source	Fructose used as carbon source	Galactose used as carbon source	Galacturonic acid used as carbon s	Gluconate used as carbon source	Glucose used as carbon source	Glutaric acid used as carbon source	Histidine used as carbon source	Lysine used as carbon source	Maltose used as carbon source	Mannose used as carbon source	Melibiose used as carbon source	Melezitose used as carbon source	Mesoerythritol used as carbon source	Proline used as carbon source	Propionic acid used as carbon source	Quinic acid used as carbon source	Raffinose used as carbon source	Rhamnose used as carbon source	Salicine used as carbon source	Serine used as carbon source	Sorbitol used as carbon source	Sorbose used as carbon source	Sucrose used as carbon source	Trehalose used as carbon source	Valine used as carbon source	Xylitol used as carbon source	Xylose used as carbon source	Aesculin degradation	Arbutin degradation	Casein degradation	Catalase production	Gelatin degradation	Oxidase production	Starch degradation	Tween 20 degradation	Tween 80 degradation	Tyrosine degradation	Urea degradation	Xylan degradation	Growth at 4ºC	Growth at 12ºC	Growth at 20ºC	Growth at 37ºC	Growth at 45ºC
M. aurantiaca [1]	0	1	1	1	1	1	0	0	1	0	0	0	1	1	1	1	0	0	0	0	1	0	0	1	0	0	1	0	0	0	0	1	1	1	1	1	0	1	1	0	1	0	1	0	1	1	1	1
M. auratinigra [1]	0	1	1	1	0	1	0	1	1	0	0	1	1	1	0	1	0	0	0	0	1	1	1	1	0	0	1	0	0	0	0	1	0	1	1	0	0	1	1	0	0	0	1	0	1	1	1	1
M. carbonacea [1]	0	1	0	1	1	1	0	0	1	0	0	0	1	1	1	1	0	0	1	0	1	0	1	0	0	1	1	0	0	0	0	1	1	1	0	1	1	1	1	0	1	0	1	0	1	1	1	1
M. chaiyaphumensis [10]	?	1	?	?	1	1	?	?	1	?	?	?	?	?	1	?	?	?	?	?	1	0	1	?	?	?	?	?	?	?	1	?	?	1	?	1	?	1	?	?	1	?	?	0	0	0	1	1
M. chalcea [1]	0	0	1	0	0	1	0	0	1	0	0	0	1	1	0	1	0	1	0	0	0	0	1	0	0	0	0	0	0	0	0	1	1	1	1	0	0	1	1	0	1	0	1	0	0	1	1	0
M. chersina [1]	0	1	0	1	1	1	0	0	1	0	0	1	1	1	1	1	0	1	0	0	1	0	0	1	0	1	1	0	0	0	0	1	0	1	1	1	1	1	1	0	1	0	1	0	0	1	1	1
M. chokoriensis [1]	0	1	0	1	1	1	0	0	1	0	0	0	1	1	1	1	0	1	0	0	1	0	1	0	0	1	1	0	0	0	0	1	1	1	1	1	1	0	1	0	0	0	0	0	1	1	1	1
M. citrea [1]	0	1	1	1	0	1	0	0	1	0	0	0	1	1	0	1	0	1	0	0	1	0	1	1	0	0	1	0	0	0	0	0	1	1	1	1	1	1	1	0	1	1	0	0	1	1	1	1
M. coriariae [1]	0	1	1	1	1	1	0	0	1	0	0	0	1	1	0	0	0	1	0	0	1	1	1	1	0	0	1	0	0	0	0	1	1	1	1	1	1	1	1	0	0	1	1	0	1	1	1	1
M. coxensis [1]	0	1	1	1	1	1	0	0	1	0	0	0	1	1	1	1	0	1	1	0	1	0	1	0	0	1	1	0	0	0	0	1	1	1	1	1	1	1	1	0	0	1	1	0	1	1	1	1
M. cremea [1]	1	1	1	0	1	0	0	0	1	0	0	1	1	1	0	1	1	0	0	0	1	1	1	1	0	0	1	1	1	0	0	1	0	1	1	1	1	1	0	1	0	0	1	0	1	1	1	0
M. eburnea [1]	0	0	0	0	1	1	0	0	1	0	0	0	0	1	0	1	0	0	0	0	0	0	0	0	0	0	1	0	0	0	0	1	1	1	1	1	1	1	1	0	1	1	1	0	0	1	1	1
M. echinaurantiaca [1]	0	1	1	1	1	1	0	0	1	0	0	0	1	1	1	0	0	1	0	0	1	0	1	1	0	0	1	0	0	0	0	1	1	1	1	1	1	1	1	0	1	1	1	0	1	1	1	1
M. echinofusca [1]	0	0	1	1	1	1	0	0	1	0	0	1	1	1	0	1	0	1	1	0	1	0	1	1	0	0	1	0	1	0	0	1	1	1	1	0	1	1	1	0	1	1	1	1	1	1	1	1
M. echinospora [1]	0	1	0	1	1	1	0	0	1	0	0	0	0	0	0	1	0	0	0	0	0	1	0	0	0	0	0	0	0	0	0	1	1	1	0	1	1	1	1	0	0	0	1	0	1	1	1	1
M. endolithica [1]	0	1	1	0	0	0	0	1	1	0	0	1	0	0	0	0	0	0	0	0	1	0	1	1	0	0	0	0	0	0	0	1	0	1	0	0	1	1	1	0	0	0	1	0	1	1	1	1
M. haikouensis [25]	?	0	?	?	0	1	?	?	1	?	?	?	?	0	1	?	?	?	?	?	1	1	1	?	0	?	1	1	?	?	0	1	?	?	?	0	?	1	?	?	?	?	?	?	?	?	?	?
M. halophytica [1]	0	0	1	1	1	1	0	0	1	0	0	0	1	1	1	0	0	1	0	0	1	0	0	1	0	0	1	0	0	0	0	1	1	1	1	1	0	1	1	0	1	0	1	0	1	1	1	1
M. humi [20]	?	1	?	0	0	1	?	?	1	?	?	?	?	?	0	?	?	?	?	?	0	0	0	?	?	?	?	?	?	?	?	?	?	1	?	1	?	1	?	?	0	?	?	0	0	1	0	0
M. inositola [1]	0	1	1	1	1	1	0	0	1	0	0	0	1	1	1	0	0	0	0	0	1	0	0	1	0	0	1	0	0	0	0	1	1	1	1	1	1	1	0	0	1	0	0	0	1	1	1	1
M. inyonensis [14]	?	1	?	?	?	?	?	?	1	?	?	?	?	?	?	?	?	?	?	?	1	1	?	?	?	?	1	?	?	?	1	?	?	?	?	?	?	?	?	?	?	?	?	?	?	?	?	?
M. krabiensis [26]	?	1	?	?	?	1	?	?	1	?	?	?	?	?	1	?	?	?	?	?	1	1	1	?	?	?	?	?	?	?	1	?	?	1	?	1	?	1	?	?	0	?	?	0	0	0	1	1
M. lupini [1]	0	1	0	1	1	1	0	0	1	0	0	0	1	1	0	1	0	0	0	0	1	0	1	0	0	1	1	0	0	0	0	1	1	1	1	1	1	1	1	0	1	0	1	1	1	1	1	1
M. marina [27]	?	1	?	?	1	1	?	?	1	?	?	?	?	?	1	?	?	?	?	?	1	0	0	?	?	?	?	?	?	?	1	?	?	1	?	1	?	1	?	?	1	?	?	0	0	1	1	1
M. matsumotoense [1]	0	1	1	1	1	1	0	0	1	0	0	0	0	1	1	1	0	1	0	0	1	0	0	0	0	1	1	0	0	0	0	1	0	1	1	1	1	1	1	0	1	0	1	1	1	1	1	1
M. mirobrigensis [1]	0	1	1	1	0	0	0	0	1	0	0	0	1	1	1	1	0	1	0	0	1	1	1	1	0	0	1	0	0	0	0	1	1	1	1	1	0	1	1	0	1	0	1	1	1	1	1	1
M. narathiwatensis [17]	?	1	?	1	0	1	?	?	1	?	?	?	?	?	1	?	?	?	?	?	1	0	1	?	?	?	?	?	?	?	1	?	?	1	?	1	?	1	?	?	?	?	?	0	0	0	1	0
M. nigra [1]	0	1	0	1	0	0	0	0	1	0	0	1	0	1	1	1	0	1	0	0	1	0	0	0	0	0	1	0	0	0	0	1	1	1	1	1	1	1	1	0	1	1	1	0	1	1	1	1
M. olivasterospora [1]	0	1	0	1	1	1	0	0	1	0	0	1	1	1	1	1	0	1	0	0	1	0	0	1	0	0	1	0	1	0	0	1	1	1	1	1	1	1	1	0	1	0	0	0	0	1	1	1
M. pallida [1]	0	1	0	1	1	1	0	0	1	0	0	0	0	1	0	0	0	0	0	0	0	1	0	0	0	0	0	0	0	0	0	1	1	1	0	1	1	1	1	0	0	0	1	0	0	1	1	0
M. peucetia [1]	0	1	1	1	1	0	0	0	1	0	0	0	0	1	0	0	0	1	0	0	0	0	1	1	0	0	1	0	0	0	0	1	1	1	1	1	1	1	1	0	1	0	1	1	1	1	1	0
M. purpureochromogenes [1]	0	1	1	1	1	1	0	0	1	0	0	1	1	1	1	0	0	1	0	0	1	0	0	1	0	1	0	0	0	0	0	1	1	0	1	1	1	1	1	0	1	0	0	0	0	0	1	0
M. rhizosphaerae [23]	?	0	?	?	1	1	?	?	1	?	?	?	0	?	0	0	?	?	?	?	0	0	0	?	?	?	0	?	?	?	1	?	?	?	?	0	?	0	?	?	?	?	?	0	1	1	1	0
M. rifamycinica [1]	0	0	1	1	1	1	0	0	1	0	0	0	1	0	1	1	0	0	0	0	1	0	1	0	0	1	1	0	0	0	0	1	1	1	1	1	1	1	1	0	1	0	1	0	1	1	1	1
M. saelicesensis [1]	1	1	1	1	1	1	0	0	1	0	0	0	1	1	1	1	0	1	0	0	1	0	1	1	0	1	1	0	1	0	0	1	1	1	1	1	1	1	1	0	1	1	1	0	1	1	1	1
M. sagamiensis [1]	0	0	1	0	0	1	0	0	1	0	0	0	0	0	0	0	0	0	0	0	0	0	1	1	0	0	0	0	0	0	0	1	1	1	1	0	1	1	1	0	0	0	1	0	0	0	1	1
M. sediminicola [3]	?	?	?	?	?	?	?	?	?	?	?	?	?	?	?	?	?	?	?	?	?	?	?	?	?	?	?	?	?	?	?	?	?	?	?	1	?	1	?	?	?	?	?	0	0	1	1	0
M. siamensis [1]	0	1	1	0	1	1	0	0	1	0	0	0	0	0	0	0	0	1	1	0	1	1	1	1	0	0	1	0	1	0	0	1	1	1	1	0	1	1	1	0	0	0	0	0	1	1	1	1
M. tulbaghiae [28]	?	0	?	0	1	?	?	?	1	?	?	?	?	?	0	?	?	?	?	?	0	1	0	?	?	?	?	?	?	?	0	?	?	1	?	1	?	1	?	1	0	?	1	1	1	1	1	0
M. viridifaciens [1]	0	1	1	0	0	0	0	0	1	0	0	0	1	1	0	0	0	0	0	0	0	0	1	1	0	0	0	0	0	0	0	1	0	1	1	1	0	1	1	0	1	0	0	0	0	1	1	1
M. yangpuensis [29]	?	0	?	0	0	1	?	?	1	?	?	?	0	1	?	?	?	?	?	?	1	0	0	?	?	?	1	1	?	?	1	1	0	1	1	0	0	1	1	1	?	?	?	0	1	1	1	1
M. zamorensis [1]	1	1	1	1	1	0	0	1	1	0	0	0	1	1	1	1	0	1	1	0	1	1	1	1	0	0	1	1	1	0	0	1	1	1	1	1	1	1	1	1	1	0	1	0	1	1	1	0

	Growth at 4.4 pH	Growth at 5.4 pH	Growth at 6.4 pH	Growth at 8 pH	Growth at 9 pH	Growth at 1% NaCl	Growth at 3% NaCl	Growth at 5% NaCl	Growth at 7% NaCl	Growth at 9% NaCl	Contains m-DAP	Contains 3OH-DAP	Contains Xylose	Contains Arabinose	Contains Glucose	Contains Ribose	Contains Mannose	Contains Galactose	Contains Rhamnose	Contains 3-O-methylrhamnose	Contains MK-7 (H6)	Contains MK-7 (H8)	Contains MK-8 (H4)	Contains MK-8 (H6)	Contains MK-8 (H8)	Contains MK-9 (H2)	Contains MK-9 (H4)	Contains MK-9 (H6)	Contains MK-9 (H8)	Contains MK-9 (H10)	Contains MK-10 (H0)	Contains MK-10 (H2)	Contains MK-10 (H4)	Contains MK-10 (H6)	Contains MK-10 (H8)	Contains MK-11 (H4)	Contains MK-12	Contains DPG	Contains PI	Contains PE	Contains PIM	Contains PG	Contains PME	Contains GPLs	Contains PL
M. aurantiaca [1,30]	0	1	1	1	1	1	1	0	0	0	1	0	?	?	?	?	?	?	?	?	0	0	0	0	0	0	0	0	0	0	0	0	1	0	0	0	0	?	?	?	?	?	?	?	?
M. auratinigra [1,5]	0	1	1	1	1	1	1	0	0	0	1	1	1	1	1	1	1	1	0	0	0	0	0	0	0	0	1	0	0	0	0	0	1	1	0	0	0	1	1	1	1	0	0	0	0
M. carbonacea [1,12,25,30]	0	0	1	1	1	1	1	0	0	0	1	1	1	1	1	0	0	0	0	0	0	0	1	0	0	0	1	1	0	0	0	0	1	1	0	0	0	1	0	1	1	0	0	1	0
M. chaiyaphumensis [10]	1	1	1	1	1	1	1	0	0	0	1	0	1	1	1	1	1	1	0	0	0	0	0	0	0	0	0	1	1	0	0	0	0	1	1	0	0	1	1	1	1	0	0	0	0
M. chalcea [1,30]	0	0	0	1	1	1	0	0	0	0	1	0	?	?	?	?	?	?	?	?	0	0	0	0	0	0	0	0	0	0	0	0	1	1	0	0	0	?	?	?	?	?	?	?	?
M. chersina [1,12]	0	0	1	1	1	1	0	0	0	0	1	0	1	1	1	0	1	0	0	0	0	0	0	0	0	0	1	1	0	0	0	0	1	1	0	0	0	0	1	1	0	0	0	0	0
M. chokoriensis [1,13]	0	0	1	1	1	1	0	0	0	0	1	0	1	1	1	1	1	1	0	0	0	0	0	0	0	0	1	1	0	0	0	0	1	1	0	0	0	1	1	1	1	1	0	0	0
M. citrea [1,14]	0	1	1	1	1	1	1	0	0	0	1	0	1	0	1	0	0	0	0	0	0	0	0	0	0	0	0	0	0	0	0	0	1	0	0	0	0	0	0	1	0	0	0	0	0
M. coriariae [1,15]	0	0	0	1	1	1	0	0	0	0	1	0	1	1	1	1	1	0	0	0	0	0	0	0	0	0	1	0	0	0	0	0	1	1	0	0	0	1	1	1	0	0	0	1	0
M. coxensis [1,13]	0	0	1	1	1	1	1	0	0	0	1	0	1	1	1	1	1	1	1	0	0	0	0	0	0	0	1	1	1	0	0	0	1	1	1	0	0	1	1	1	1	1	0	0	0
M. cremea [1,16]	0	0	0	1	0	1	0	0	0	0	1	0	1	0	1	1	1	1	1	0	0	0	0	0	0	0	1	1	0	0	0	0	1	1	0	0	0	1	1	1	0	0	0	1	0
M. eburnea [1,31]	0	1	1	1	1	1	1	0	0	0	1	0	1	1	1	1	1	1	0	0	0	0	0	0	0	1	1	1	1	0	0	0	1	1	1	0	0	1	1	1	1	0	0	0	0
M. echinaurantiaca [1,14]	0	0	0	1	1	1	1	0	0	0	1	0	1	0	1	0	0	0	1	0	0	0	0	0	0	0	1	0	0	0	0	0	0	0	0	0	0	0	0	1	0	0	0	0	0
M. echinofusca [1,14]	0	0	1	1	1	1	1	0	0	0	1	0	1	0	1	0	0	0	0	0	0	0	0	0	0	0	1	0	0	0	0	0	0	0	0	0	0	0	0	1	0	0	0	0	0
M. echinospora [1,30,32]	0	0	1	1	1	1	0	0	0	0	1	1	?	?	?	?	?	?	?	?	0	0	0	0	0	0	0	0	0	0	0	0	1	1	0	0	0	?	?	?	?	?	?	?	?
M. endolithica [1,18]	0	0	0	1	1	1	1	0	0	0	1	0	1	1	0	1	0	1	1	0	0	0	0	0	0	0	0	0	0	0	0	0	1	1	0	0	0	1	1	1	0	0	0	0	0
M. haikouensis [25]	0	1	1	1	1	1	1	0	0	0	1	0	1	1	1	0	0	0	0	0	0	0	0	0	0	0	0	0	0	0	0	0	1	1	0	0	0	1	0	1	1	0	0	0	0
M. halophytica [1,30]	0	0	0	1	1	1	1	0	0	0	1	1	?	?	?	?	?	?	?	?	0	0	0	0	0	0	1	0	0	0	0	0	0	0	0	0	0	?	?	?	?	?	?	?	?
M. humi [20]	0	1	1	1	0	1	1	1	0	0	1	0	1	1	1	1	1	1	1	0	0	0	0	0	0	0	0	0	0	0	0	0	1	1	0	0	0	1	1	1	1	0	0	0	0
M. inositola [1,30]	0	0	0	1	1	1	1	0	0	0	1	1	?	?	?	?	?	?	?	?	0	0	0	0	0	0	0	0	0	0	0	0	1	0	0	0	0	?	?	?	?	?	?	?	?
M. inyonensis [14]	?	?	?	?	?	?	?	?	?	?	1	0	1	1	1	0	0	0	0	0	0	0	0	0	0	0	0	0	0	0	0	0	1	0	0	0	0	0	0	1	0	0	0	0	0
M. krabiensis [26]	0	1	1	1	1	1	1	0	0	0	1	0	1	1	1	1	1	1	0	0	0	0	0	0	0	0	0	0	0	0	0	0	1	1	0	0	0	1	1	1	0	0	0	0	0
M. lupini [1,21]	0	0	1	1	1	1	0	0	0	0	1	0	1	1	1	0	1	0	1	0	0	0	0	0	0	0	0	0	0	0	0	0	1	1	0	0	0	1	1	1	0	0	0	0	0
M. marina [27]	0	1	1	1	1	1	1	1	1	0	1	0	1	1	1	1	1	1	0	0	0	0	0	0	0	0	0	0	0	0	0	0	1	1	0	0	0	1	1	1	1	0	0	0	0
M. matsumotoense [1,22,30]	0	0	0	1	1	1	1	0	0	0	1	1	1	1	0	0	0	1	0	1	0	0	0	0	0	0	0	0	0	0	0	0	1	1	1	0	0	1	1	1	0	1	0	0	1
M. mirobrigensis [1,6]	0	0	0	1	1	1	1	0	0	0	1	0	1	0	1	0	1	1	0	0	0	0	0	0	0	0	0	0	0	0	0	0	1	1	0	0	0	1	1	1	0	1	0	1	0
M. narathiwatensis [17]	?	?	?	?	?	1	1	0	0	0	1	0	1	1	0	0	0	0	0	0	0	0	0	0	0	0	0	0	0	0	0	0	1	1	1	0	0	1	1	1	1	0	0	0	0
M. nigra [1,30]	0	0	0	1	0	1	1	0	0	0	1	1	?	?	?	?	?	?	?	?	0	0	0	0	0	0	1	0	0	0	0	0	0	0	0	0	0	?	?	?	?	?	?	?	?
M. olivasterospora [1,30,33]	0	0	1	1	1	1	1	0	0	0	1	1	1	1	1	0	0	0	0	0	0	0	0	0	0	0	0	0	0	0	0	0	1	0	0	0	0	1	1	1	1	0	0	0	0
M. pallida [1,30]	0	0	0	1	0	1	0	0	0	0	1	1	?	?	?	?	?	?	?	?	0	0	0	0	0	0	0	0	0	0	0	0	0	0	0	0	1	?	?	?	?	?	?	?	?
M. peucetia [1,14]	0	0	1	1	1	1	1	0	0	0	1	0	1	1	1	0	0	0	0	0	0	0	0	0	0	0	1	0	0	0	0	0	0	0	0	0	0	0	0	1	0	0	0	0	0
M. purpureochromogenes [1,30]	0	0	0	1	0	1	0	0	0	0	1	0	?	?	?	?	?	?	?	?	0	0	0	0	0	0	0	0	0	0	0	0	1	0	0	0	0	?	?	?	?	?	?	?	?
M. rhizosphaerae [23]	0	1	1	1	1	1	0	0	0	0	1	0	1	1	1	1	1	0	0	0	0	0	0	0	0	1	1	1	0	0	0	0	0	0	0	0	0	0	1	1	1	1	0	0	0
M. rifamycinica [1,25,34]	0	0	0	1	1	1	0	0	0	0	1	0	1	1	0	0	0	0	0	0	0	0	0	0	0	0	0	0	0	0	0	0	1	1	0	0	0	1	0	1	1	0	0	1	0
M. saelicesensis [1,21]	0	0	0	1	1	1	0	0	0	0	1	0	1	1	1	1	1	0	1	0	0	0	0	0	0	0	0	0	0	0	0	0	1	0	0	0	0	1	1	1	0	0	0	0	0
M. sagamiensis [1,14,30]	0	0	0	1	1	1	0	0	0	0	1	0	1	1	1	1	1	0	1	0	0	0	0	0	0	0	1	0	0	0	0	0	0	0	0	0	0	0	0	1	0	0	0	0	0
M. sediminicola [3]	0	0	1	1	1	1	1	0	0	0	1	0	1	1	1	1	0	1	1	0	0	0	0	0	0	0	0	0	0	0	0	1	1	1	0	0	0	1	1	1	1	0	0	0	0
M. siamensis [1,35]	0	0	0	1	1	1	1	0	0	0	1	0	1	1	0	0	0	0	0	0	0	0	0	0	0	0	0	0	0	0	0	0	1	1	0	0	0	1	1	1	1	0	0	0	0
M. tulbaghiae [28]	0	0	0	1	1	1	1	1	0	0	1	0	1	1	1	0	0	0	0	0	?	?	?	?	?	?	?	?	?	?	?	?	?	?	?	?	?	?	?	?	?	?	?	?	?
M. viridifaciens [1,14]	0	0	1	1	1	1	1	1	0	0	1	0	1	0	1	0	0	0	1	0	0	0	0	0	0	0	1	0	0	0	0	0	0	0	0	0	0	0	0	1	0	0	0	0	0
M. yangpuensis [29]	0	0	1	1	1	1	1	0	0	0	1	0	1	1	1	1	1	1	1	0	0	0	0	0	0	0	0	0	0	0	1	1	1	0	0	0	0	1	1	1	1	0	0	1	1
M. zamorensis [1,16]	0	0	1	1	1	1	1	0	0	0	1	0	1	0	1	1	1	0	0	0	0	0	0	0	0	0	0	0	0	0	0	0	1	1	0	0	0	1	1	1	0	0	0	0	0

Positive: 1; Negative: 0; Not determined: ?

[] Reference equivalence in supplementary reference list.

Supplementary Table 6. ENVO classification of habitats from which the *Micromonospora* strains were isolated.

	Habitat of isolation	Country	ENVO A	ENVO B	ENVOB ID	ENVOC	ENVOC ID	ENVOD	ENVOD ID	ENVOE	ENVOE ID	ENVOF	ENVOF ID	Saline
M. aurantiaca ATCC 27029^{T}	Unclassified soil	Unknown	Environmental material	Soil	ENVO:00001998	?	?	?	?	?	?	?	?	0
M. aurantiaca L5	Plant endophytic	Mexico	Environmental material	Organic material	ENVO:01000155	Root matter	ENVO:01000349	?	?	?	?	?	?	0
M. aurantiaca DSM 45487	Humic soil	Iran	Environmental material	Soil	ENVO:00001998	Humus	ENVO:01000000	?	?	?	?	?	?	0
M. auratinigra DSM 44815^{T}	Peat swamp forest soil	Thailand	Environmental material	Soil	ENVO:00001998	Forest soil	ENVO:00002261	?	?	?	?	?	?	0
M. carbonacea DSM 43168^{T}	Unclassified soil	USA	Environmental material	Soil	ENVO:00001998	?	?	?	?	?	?	?	?	0
M. chaiyaphumensis DSM 45246^{T}	Mountain soil	Thailand	Environmental material	Soil	ENVO:00001998	Alpine soil	ENVO:00005741	?	?	?	?	?	?	0
M. chalcea DSM 43026^{T}	Air	UK	Environmental material	Gaseous environmental material	ENVO:01000797	Air	ENVO:00002005	?	?	?	?	?	?	0
M. chersina DSM 44151^{T}	Unclassified soil	India	Environmental material	Soil	ENVO:00001998	?	?	?	?	?	?	?	?	0
M. chokoriensis DSM 45160^{T}	Sandy soil	Bangladesh	Environmental material	Soil	ENVO:00001998	?	?	?	?	?	?	?	?	0
M. citrea DSM 43903^{T}	Lake mud	China	Environmental material	Mud	ENVO:01000001	Lake bottom mud	ENVO:00005797	?	?	?	?	?	?	0
M. coriariae DSM 44875^{T}	Plant endophytic	Spain	Environmental material	Organic material	ENVO:01000155	Root matter	ENVO:01000349	?	?	?	?	?	?	0
M. coxensis DSM 45161^{T}	Sandy soil	Bangladesh	Environmental material	Soil	ENVO:00001998	?	?	?	?	?	?	?	?	0
M. cremea DSM 45599^{T}	Rhizospheric soil	Spain	Environmental material	Soil	ENVO:00001998	Agricultural soil	ENVO:00002259	?	?	?	?	?	?	0
M. eburnea DSM 44814^{T}	Peat swamp forest soil	Thailand	Environmental material	Soil	ENVO:00001998	Forest soil	ENVO:00002261	?	?	?	?	?	?	0
M. echinaurantiaca DSM 43904^{T}	Unclassified soil	China	Environmental material	Soil	ENVO:00001998	?	?	?	?	?	?	?	?	0
M. echinofusca DSM 43913^{T}	Chukar excrement	China	Environmental material	Organic material	ENVO:01000155	Bodily fluid	ENVO:02000019	Excreta	ENVO:02000022	Feces	ENVO:00002003	?	?	0
M. echinospora DSM 1040	Unclassified soil	USA	Environmental material	Soil	ENVO:00001998	?	?	?	?	?	?	?	?	0
M. echinospora DSM 43816^{T}	Unclassified soil	USA	Environmental material	Soil	ENVO:00001998	?	?	?	?	?	?	?	?	0
M. endolithica DSM 44398^{T}	Sandstone rock	Antarctica	Environmental material	Solid environmental material	ENVO:01000814	Rock	ENVO:00001995	Sedimentary rock	ENVO:00002016	Clastic sedimentary rock	ENVO:01000271	Sandstone	ENVO:00002055	0
M. haikouensis DSM 45626^{T}	Mangrove soil	China	Environmental material	Sediment	ENVO:00002007	Marine sediment	ENVO:00002113	?	?	?	?	?	?	1
M. halophytica DSM 43171^{T}	Salt pond	USA	Environmental material	Liquid environmental material	ENVO:01000815	Water	ENVO:00002006	Saline water	ENVO:00002010	?	?	?	?	1
M. humi DSM 45647^{T}	Peat swamp forest soil	Thailand	Environmental material	Soil	ENVO:00001998	Forest soil	ENVO:00002261	?	?	?	?	?	?	0
M. inositola DSM 43819^{T}	Forest soil	Japan	Environmental material	Soil	ENVO:00001998	Forest soil	ENVO:00002261	?	?	?	?	?	?	0
M. inyonensis DSM 46123^{T}	Unclassified soil	USA	Environmental material	Soil	ENVO:00001998	?	?	?	?	?	?	?	?	0
M. krabiensis DSM 45344^{T}	Marine soil	Thailand	Environmental material	Sediment	ENVO:00002007	Marine sediment	ENVO:00002113	?	?	?	?	?	?	1
M. lupini Lupac 08	Plant endophytic	Spain	Environmental material	Organic material	ENVO:01000155	Root matter	ENVO:01000349	?	?	?	?	?	?	0
M. marina DSM 45555^{T}	Sea sand	Thailand	Environmental material	Sand	ENVO:01000017	Sea sand	ENVO:00002118	?	?	?	?	?	?	1
M. matsumotoense DSM 44100^{T}	Forest soil	Japan	Environmental material	Soil	ENVO:00001998	Forest soil	ENVO:00002261	?	?	?	?	?	?	0
M. mirobrigensis DSM 44830^{T}	Pond	Spain	Environmental material	Liquid environmental material	ENVO:01000815	Water	ENVO:00002006	Fresh water	ENVO:00002011	Pond water	ENVO:00002228	?	?	0
M. narathiwatensis DSM 45248^{T}	Peat swamp forest soil	Thailand	Environmental material	Soil	ENVO:00001998	Forest soil	ENVO:00002261	?	?	?	?	?	?	0
M. nigra DSM 43818^{T}	Salt pond	USA	Environmental material	Liquid environmental material	ENVO:01000815	Water	ENVO:00002006	Saline water	ENVO:00002010	?	?	?	?	1
M. olivasterospora DSM 43868^{T}	Unclassified soil	Japan	Environmental material	Soil	ENVO:00001998	?	?	?	?	?	?	?	?	0
M. pallida DSM 43817^{T}	Unclassified soil	USA	Environmental material	Soil	ENVO:00001998	?	?	?	?	?	?	?	?	0
M. peucetia DSM 43363^{T}	Unclassified soil	Italy	Environmental material	Soil	ENVO:00001998	?	?	?	?	?	?	?	?	0
M. purpureochromogenes DSM 43821^{T}	Adobe soil	USA	Environmental material	Soil	ENVO:00001998	Clay soil	ENVO:00002262	?	?	?	?	?	?	0
M. rhizosphaerae DSM 45431^{T}	Mangrove rhizospheric soil	China	Environmental material	Sediment	ENVO:00002007	Marine sediment	ENVO:00002113	?	?	?	?	?	?	1
M. rifamycinica DSM 44983^{T}	Mangrove soil	China	Environmental material	Sediment	ENVO:00002007	Marine sediment	ENVO:00002113	?	?	?	?	?	?	1
M. saelicesensis DSM 44871^{T}	Plant endophytic	Spain	Environmental material	Organic material	ENVO:01000155	Root matter	ENVO:01000349	?	?	?	?	?	?	0
M. sagamiensis DSM 43912^{T}	Forest soil	Japan	Environmental material	Soil	ENVO:00001998	Forest soil	ENVO:00002261	?	?	?	?	?	?	0
M. sediminicola DSM 45794^{T}	Marine soil	Thailand	Environmental material	Sediment	ENVO:00002007	Marine sediment	ENVO:00002113	?	?	?	?	?	?	1
M. siamensis DSM 45097^{T}	Peat swamp forest soil	Thailand	Environmental material	Soil	ENVO:00001998	Forest soil	ENVO:00002261	?	?	?	?	?	?	0
M. tulbaghiae DSM 45142^{T}	Plant endophytic	South Africa	Environmental material	Organic material	ENVO:01000155	?	?	?	?	?	?	?	?	0
M. viridifaciens DSM 43909^{T}	Unclassified soil	Japan	Environmental material	Soil	ENVO:00001998	?	?	?	?	?	?	?	?	0
M. yangpuensis DSM 45577^{T}	Sea sponge endophytic	China	Environmental material	Organic material	ENVO:01000155	?	?	?	?	?	?	?	?	1
M. zamorensis DSM 45600^{T}	Rhizospheric soil	Spain	Environmental material	Soil	ENVO:00001998	Agricultural soil	ENVO:00002259	?	?	?	?	?	?	0

Supplementary Table 7. Bioclusters found in antiSMASH for the genus*Micromonospora.*

Cluster	Type	From	To	Most similar known cluster	MIBiG BGC-ID
M. aurantiaca ATCC 27029[T]					
Cluster 1	Terpene	235554	256480	Sioxanthin_biosynthetic_gene_cluster (100% of genes show similarity)	BGC0001087_c4
Cluster 2	Terpene	339680	360645	Phosphonoglycans_biosynthetic_gene_cluster (3% of genes show similarity)	BGC0000806_c1
Cluster 3	Nrps	1912509	1963699	Landepoxcin_biosynthetic_gene_cluster (11% of genes show similarity)	BGC0001202_c1
Cluster 4	Nrps-T1pks	2595494	2826070	Rifamycin_biosynthetic_gene_cluster (35% of genes show similarity)	BGC0000137_c1
Cluster 5	T1pks	2830112	2875961	Dynemicin_biosynthetic_gene_cluster (10% of genes show similarity)	BGC0001060_c1
Cluster 6	Siderophore-Nrps-Lantipeptic	2949892	3053218	Azicemicin_biosynthetic_gene_cluster (13% of genes show similarity)	BGC0000202_c1
Cluster 7	Nrps-Lantipeptide-T1pks	3146498	3214124	Bleomycin_biosynthetic_gene_cluster (12% of genes show similarity)	BGC0000963_c1
Cluster 8	Terpene	3679147	3700349	Nocathiacin_biosynthetic_gene_cluster (4% of genes show similarity)	BGC0000609_c1
Cluster 9	T2pks	3897736	3940248	Xantholipin_biosynthetic_gene_cluster (16% of genes show similarity)	BGC0000279_c1
Cluster 10	Nrps-Oligosaccharide-Terpen	4131522	4194143	Lobosamide_biosynthetic_gene_cluster (13% of genes show similarity)	BGC0001303_c1
Cluster 11	Otherks-Nrps-Transatpks	4352067	4436987	Leinamycin_biosynthetic_gene_cluster (15% of genes show similarity)	BGC0001101_c1
Cluster 12	Oligosaccharide-T2pks	4502288	4584222	Cosmomycin_D_biosynthetic_gene_cluster (55% of genes show similarity)	BGC0001074_c1
Cluster 13	Lantipeptide	4649877	4674387	-	-
Cluster 14	Terpene-Bacteriocin	4851740	4881322	Lymphostin_biosynthetic_gene_cluster (33% of genes show similarity)	BGC0001006_c1
Cluster 15	Terpene	5021463	5042413	-	-
Cluster 16	T3pks	6489908	6530969	Alkyl-O-Dihydrogeranyl-Methoxyhydroquinones_biosynthetic_gene (71% of genes show similarity)	BGC0001077_c1
Cluster 17	Lantipeptide	6829224	6866976	-	-
M. aurantiaca L5					
Cluster 1	Terpene	221501	242427	Sioxanthin_biosynthetic_gene_cluster (100% of genes show similarity)	BGC0001087_c4
Cluster 2	Terpene	313323	334288	Phosphonoglycans_biosynthetic_gene_cluster (3% of genes show similarity)	BGC0000806_c1
Cluster 3	Nrps	821484	876445	Herboxidiene_biosynthetic_gene_cluster (2% of genes show similarity)	BGC0001065_c1
Cluster 4	T3pks	2761821	2802873	Alkyl-O-Dihydrogeranyl-Methoxyhydroquinones_biosynthetic_gene (71% of genes show similarity)	BGC0001077_c1
Cluster 5	Terpene	4057896	4078846	-	-
Cluster 6	Bacteriocin-Terpene	4219887	4244469	Lymphostin_biosynthetic_gene_cluster (33% of genes show similarity)	BGC0001006_c1
Cluster 7	Siderophore	4603072	4616283	-	-
Cluster 8	Oligosaccharide-T2pks	4667647	4739578	Cinerubin_B_biosynthetic_gene_cluster (62% of genes show similarity)	BGC0000212_c1
Cluster 9	Transatpks-Nrps-Otherks	4801291	4886348	Leinamycin_biosynthetic_gene_cluster (15% of genes show similarity)	BGC0001101_c1
Cluster 10	Oligosaccharide-Nrps-Terpen	5040049	5089051	Lobosamide_biosynthetic_gene_cluster (10% of genes show similarity)	BGC0001303_c1
Cluster 11	T2pks	5284338	5326912	Xantholipin_biosynthetic_gene_cluster (14% of genes show similarity)	BGC0000279_c1
Cluster 12	Terpene	5524214	5545416	Nocathiacin_biosynthetic_gene_cluster (4% of genes show similarity)	BGC0000609_c1
Cluster 13	Nrps-T1pks-Lantipeptide	6013883	6081509	Bleomycin_biosynthetic_gene_cluster (12% of genes show similarity)	BGC0000963_c1
Cluster 14	Otherks-Lantipeptide-Nrps-T	6169329	6288581	Azicemicin_biosynthetic_gene_cluster (13% of genes show similarity)	BGC0000202_c1
Cluster 15	T1pks	6308247	6354096	Dynemicin_biosynthetic_gene_cluster (8% of genes show similarity)	BGC0001060_c1
Cluster 16	T1pks-Nrps	6358138	6579562	Rifamycin_biosynthetic_gene_cluster (35% of genes show similarity)	BGC0000137_c1
M. aurantiaca DSM 45487					
Cluster 1	Nrps-Otherks	1	43519	Leinamycin_biosynthetic_gene_cluster (11% of genes show similarity)	BGC0001101_c1
Cluster 2	Oligosaccharide-Nrps-Terpen	197129	246131	Brasilicardin_A_biosynthetic_gene_cluster (45% of genes show similarity)	BGC0000632_c1
Cluster 3	T2pks	441120	483632	Xantholipin_biosynthetic_gene_cluster (16% of genes show similarity)	BGC0000279_c1
Cluster 4	Terpene	680484	701686	Nocathiacin_biosynthetic_gene_cluster (4% of genes show similarity)	BGC0000609_c1
Cluster 5	Nrps-T1pks-Lantipeptide	1173657	1241283	Bleomycin_biosynthetic_gene_cluster (12% of genes show similarity)	BGC0000963_c1
Cluster 6	Otherks-Lantipeptide-Nrps-T	1329012	1448335	Azicemicin_biosynthetic_gene_cluster (13% of genes show similarity)	BGC0000202_c1
Cluster 7	T1pks	1468076	1513928	Dynemicin_biosynthetic_gene_cluster (8% of genes show similarity)	BGC0001060_c1
Cluster 8	T1pks-Nrps	1517972	1748550	Rifamycin_biosynthetic_gene_cluster (35% of genes show similarity)	BGC0000137_c1
Cluster 9	Terpene	4115421	4136386	-	-
Cluster 10	Terpene	4205931	4226857	Sioxanthin_biosynthetic_gene_cluster (80% of genes show similarity)	BGC0001087_c4
Cluster 11	T3pks	4829537	4870589	Alkyl-O-Dihydrogeranyl-Methoxyhydroquinones_biosynthetic_gene (71% of genes show similarity)	BGC0001077_c1
Cluster 12	Terpene	6324290	6345240	-	-
Cluster 13	Bacteriocin-Terpene	6484776	6509355	Lymphostin_biosynthetic_gene_cluster (33% of genes show similarity)	BGC0001006_c1
Cluster 14	Lantipeptide	6691540	6716110	-	-
Cluster 15	Siderophore	7038303	7051514	-	-
Cluster 16	Oligosaccharide-T2pks	7071476	7143408	Cinerubin_B_biosynthetic_gene_cluster (62% of genes show similarity)	BGC0000212_c1
Cluster 17	Transatpks	7203699	7245320	Leinamycin_biosynthetic_gene_cluster (4% of genes show similarity)	BGC0001101_c1
M. auratinigra DSM 44815[T]					
Cluster 1	Lantipeptide	733599	756289	Labyrinthopeptin_A1,A3_/_labyrinthopeptin_A2_biosynthetic_gene (40% of genes show similarity)	BGC0000519_c1
Cluster 2	Nrps-Lantipeptide-T1pks	920585	988330	Bleomycin_biosynthetic_gene_cluster (9% of genes show similarity)	BGC0000963_c1
Cluster 3	Other	1335591	1378332	-	-
Cluster 4	Terpene	1648976	1670217	Thiotetroamide_biosynthetic_gene_cluster (11% of genes show similarity)	BGC0001236_c1
Cluster 5	T2pks	2016221	2058739	Xantholipin_biosynthetic_gene_cluster (14% of genes show similarity)	BGC0000279_c1
Cluster 6	Oligosaccharide-Otherks-T2p	2233997	2307084	Polyketomycin_biosynthetic_gene_cluster (39% of genes show similarity)	BGC0001061_c1
Cluster 7	T1pks	2334134	2379884	Calicheamicin_biosynthetic_gene_cluster (22% of genes show similarity)	BGC0000033_c1
Cluster 8	Nrps-T1pks	2417599	2480322	Bleomycin_biosynthetic_gene_cluster (6% of genes show similarity)	BGC0000963_c1
Cluster 9	T1pks	2680947	2785545	Salinilactam_biosynthetic_gene_cluster (96% of genes show similarity)	BGC0000142_c1
Cluster 10	Terpene-Bacteriocin	2915636	2944873	Lymphostin_biosynthetic_gene_cluster (33% of genes show similarity)	BGC0001006_c1
Cluster 11	Terpene	3077267	3098217	-	-
Cluster 12	T3pks	4615779	4656828	Alkyl-O-Dihydrogeranyl-Methoxyhydroquinones_biosynthetic_gene (71% of genes show similarity)	BGC0001077_c1
Cluster 13	Terpene	5217690	5238616	Sioxanthin_biosynthetic_gene_cluster (100% of genes show similarity)	BGC0001087_c4
Cluster 14	Terpene	5301439	5322404	Phosphonoglycans_biosynthetic_gene_cluster (3% of genes show similarity)	BGC0000806_c1
M. carbonacea DSM 43168[T]					
Cluster 1	T3pks	151273	192322	Alkyl-O-Dihydrogeranyl-Methoxyhydroquinones_biosynthetic_gene (57% of genes show similarity)	BGC0001077_c1
Cluster 2	T2pks-Nrps-T1pks-Lantipepti	230258	314255	Xantholipin_biosynthetic_gene_cluster (12% of genes show similarity)	BGC0000279_c1
Cluster 3	Nrps-Lantipeptide-T1pks	462199	530461	Bleomycin_biosynthetic_gene_cluster (12% of genes show similarity)	BGC0000963_c1
Cluster 4	T1pks-Otherks	21443	67833	Maklamicin_biosynthetic_gene_cluster (6% of genes show similarity)	BGC0001288_c1
Cluster 5	Bacteriocin	306929	317753	Lymphostin_biosynthetic_gene_cluster (30% of genes show similarity)	BGC0001007_c1
Cluster 6	Terpene	471898	492839	-	-
Cluster 7	Nrps-T1pks	127847	177302	Frontalamides_biosynthetic_gene_cluster (57% of genes show similarity)	BGC0000996_c1
Cluster 8	Nrps	466320	525118	Asukamycin_biosynthetic_gene_cluster (6% of genes show similarity)	BGC0000187_c1
Cluster 9	Lantipeptide	32793	59751	-	-

Cluster 10	Thiopeptide	452812	491399	Siomycin_biosynthetic_gene_cluster (70% of genes show similarity)	BGC0000655_c1
Cluster 11	Terpene	101130	122137	-	-
Cluster 12	Lassopeptide	180865	202655	-	-
Cluster 13	Terpene	212664	233587	Sioxanthin_biosynthetic_gene_cluster (80% of genes show similarity)	BGC0001087_c4
Cluster 14	T1pks	1	68518	Leucanicidin_biosynthetic_gene_cluster (100% of genes show similarity)	BGC0001232_c1
Cluster 15	T1pks	26100	75549	-	-
Cluster 16	Amglyccycl	202796	224076	Validamycin_biosynthetic_gene_cluster (22% of genes show similarity)	BGC0000723_c1
Cluster 17	Lantipeptide	251127	273769	SapB_biosynthetic_gene_cluster (100% of genes show similarity)	BGC0000551_c1
Cluster 18	Siderophore	283812	296205	Desferrioxamine_B_biosynthetic_gene_cluster (66% of genes show similarity)	BGC0000940_c1
Cluster 19	T1pks	71003	116825	Sporolide_biosynthetic_gene_cluster (23% of genes show similarity)	BGC0000150_c1
Cluster 20	Terpene	116515	137705	-	-
Cluster 21	Nrps	271454	307471	Gentamicin_biosynthetic_gene_cluster (4% of genes show similarity)	BGC0000696_c1
Cluster 22	Oligosaccharide-Otherks-T1p	112223	183344	Avilamycin_A_biosynthetic_gene_cluster (55% of genes show similarity)	BGC0000026_c1
Cluster 23	Nrps	224515	271978	-	-
Cluster 24	T2pks	80199	122855	Spore_pigment_biosynthetic_gene_cluster (50% of genes show similarity)	BGC0000271_c1
Cluster 25	Arylpolyene	135381	176775	Hygromycin_A_biosynthetic_gene_cluster (24% of genes show similarity)	BGC0000698_c1
Cluster 26	Nrps	2714	57249	Oxazolomycin_biosynthetic_gene_cluster (15% of genes show similarity)	BGC0001106_c1
Cluster 27	T1pks	1	43345	Mycinamicin_biosynthetic_gene_cluster (42% of genes show similarity)	BGC0000102_c1
Cluster 28	T1pks	1	41324	Rifamycin_biosynthetic_gene_cluster (35% of genes show similarity)	BGC0000137_c1
Cluster 29	T1pks	1	41650	KS-505a_biosynthetic_gene_cluster (7% of genes show similarity)	BGC0000651_c1
Cluster 30	Nrps	67611	89782	Kinamycin_biosynthetic_gene_cluster (8% of genes show similarity)	BGC0000236_c1
Cluster 31	T1pks	1	79097	FR-008_biosynthetic_gene_cluster (19% of genes show similarity)	BGC0000061_c1
Cluster 32	Indole	1	18636	-	-
Cluster 33	Nrps-T1pks	1	40196	Actinomycin_biosynthetic_gene_cluster (10% of genes show similarity)	BGC0000296_c1
Cluster 34	T1pks	1	25403	Herboxidiene_biosynthetic_gene_cluster (3% of genes show similarity)	BGC0001065_c1
Cluster 35	Lassopeptide	1	16041	SSV-2083_biosynthetic_gene_cluster (25% of genes show similarity)	BGC0000579_c1
Cluster 36	T1pks	1	10059	Dihydrochalcomycin_biosynthetic_gene_cluster (16% of genes show similarity)	BGC0000047_c1

M. chaiyaphumensis DSM 45246^T					
Cluster 1	Terpene	279852	301111	Nystatin_biosynthetic_gene_cluster (9% of genes show similarity)	BGC0000115_c1
Cluster 2	Lantipeptide	448392	470968	SapB_biosynthetic_gene_cluster (75% of genes show similarity)	BGC0000551_c1
Cluster 3	T2pks	667645	710157	Pradimicin_biosynthetic_gene_cluster (21% of genes show similarity)	BGC0000256_c1
Cluster 4	Other	1002728	1046798	Diazepinomicin_biosynthetic_gene_cluster (7% of genes show similarity)	BGC0000679_c1
Cluster 5	Terpene	721458	750199	Phosphonoglycans_biosynthetic_gene_cluster (3% of genes show similarity)	BGC0000806_c1
Cluster 6	Nrps-T1pks-Otherks-Butyrola	59745	164621	Kedarcidin_biosynthetic_gene_cluster (38% of genes show similarity)	BGC0000081_c1
Cluster 7	Thiopeptide-Lantipeptide	166217	192580	-	-
Cluster 8	Nrps-T1pks	18205	80513	2-amino-4-methoxy-trans-3-butenoic_acid_biosynthetic_gene_cl... (20% of genes show similarity)	BGC0000287_c1
Cluster 9	Siderophore	241148	252926	Desferrioxamine_B_biosynthetic_gene_cluster (80% of genes show similarity)	BGC0000941_c1
Cluster 10	Terpene	49362	70288	Sioxanthin_biosynthetic_gene_cluster (100% of genes show similarity)	BGC0001087_c4
Cluster 11	Nrps-T1pks-Lantipeptide	251600	319262	Bleomycin_biosynthetic_gene_cluster (15% of genes show similarity)	BGC0000963_c1
Cluster 12	T3pks	158062	199111	Alkyl-O-Dihydrogeranyl-Methoxyhydroquinones_biosynthetic_gene (71% of genes show similarity)	BGC0001077_c1
Cluster 13	Terpene	104338	125288	-	-
Cluster 14	Lantipeptide	45544	76871	-	-
Cluster 15	Terpene-Bacteriocin	1826	31520	Lymphostin_biosynthetic_gene_cluster (38% of genes show similarity)	BGC0001007_c1

M. chalcea DSM 43026^T					
Cluster 1	T1pks	1	130056	Maklamicin_biosynthetic_gene_cluster (21% of genes show similarity)	BGC0001288_c1
Cluster 2	Nrps-T1pks	247383	336035	Calcimycin_biosynthetic_gene_cluster (20% of genes show similarity)	BGC0000032_c1
Cluster 3	Other	324076	364948	Diazepinomicin_biosynthetic_gene_cluster (75% of genes show similarity)	BGC0000679_c1
Cluster 4	T2pks	1	35654	Xantholipin_biosynthetic_gene_cluster (16% of genes show similarity)	BGC0000279_c1
Cluster 5	Nrps-Siderophore	24447	95884	Azicemicin_biosynthetic_gene_cluster (13% of genes show similarity)	BGC0000202_c1
Cluster 6	Lantipeptide	110881	135522	-	-
Cluster 7	T1pks	1	32377	Calicheamicin_biosynthetic_gene_cluster (13% of genes show similarity)	BGC0000033_c1
Cluster 8	Nrps	29238	84629	Elaiophylin_biosynthetic_gene_cluster (8% of genes show similarity)	BGC0000053_c1
Cluster 9	T3pks	1	22418	Alkyl-O-Dihydrogeranyl-Methoxyhydroquinones_biosynthetic_gene (28% of genes show similarity)	BGC0001077_c1
Cluster 10	T1pks	1	11815	-	-
Cluster 11	T1pks	1	38044	Naphthomycin_biosynthetic_gene_cluster (25% of genes show similarity)	BGC0000106_c1
Cluster 12	Bacteriocin-Terpene	65409	93857	Lymphostin_biosynthetic_gene_cluster (38% of genes show similarity)	BGC0001007_c1
Cluster 13	Terpene	43315	64241	Sioxanthin_biosynthetic_gene_cluster (80% of genes show similarity)	BGC0001087_c4
Cluster 14	Terpene	64655	85605	-	-
Cluster 15	Terpene	1	19393	Nocathiacin_biosynthetic_gene_cluster (4% of genes show similarity)	BGC0000609_c1
Cluster 16	Terpene	1	19701	-	-
Cluster 17	Otherks-Lantipeptide-Nrps-T	1	39510	Naphthyridinomycin_biosynthetic_gene_cluster (14% of genes show similarity)	BGC0000394_c1
Cluster 18	Nrps-Lantipeptide-T1pks	1	48439	Bleomycin_biosynthetic_gene_cluster (12% of genes show similarity)	BGC0000963_c1
Cluster 19	Lantipeptide	1	10893	-	-
Cluster 20	Oligosaccharide-Nrps-Terpen	36110	85158	Brasilicardin_A_biosynthetic_gene_cluster (54% of genes show similarity)	BGC0000632_c1

M. chersina DSM 44151^T					
Cluster 1	T3pks-Nrps	35607	142931	Enduracidin_biosynthetic_gene_cluster (33% of genes show similarity)	BGC0000341_c1
Cluster 2	T2pks	257721	300352	Xantholipin_biosynthetic_gene_cluster (16% of genes show similarity)	BGC0000279_c1
Cluster 3	Lantipeptide	489117	511699	Catenulipeptin_biosynthetic_gene_cluster (60% of genes show similarity)	BGC0000501_c1
Cluster 4	Terpene	654654	675985	Nystatin_biosynthetic_gene_cluster (9% of genes show similarity)	BGC0000115_c1
Cluster 5	Nrps-T1pks-Lantipeptide	1620544	1688299	Bleomycin_biosynthetic_gene_cluster (15% of genes show similarity)	BGC0000963_c1
Cluster 6	Siderophore	2026564	2038336	Desferrioxamine_B_biosynthetic_gene_cluster (80% of genes show similarity)	BGC0000941_c1
Cluster 7	Nrps	2175981	2237131	Maklamicin_biosynthetic_gene_cluster (4% of genes show similarity)	BGC0001288_c1
Cluster 8	Nrps-T1pks	2237017	2299646	2-amino-4-methoxy-trans-3-butenoic_acid_biosynthetic_gene_cl... (30% of genes show similarity)	BGC0000287_c1
Cluster 9	Terpene	3930588	3951547	-	-
Cluster 10	Terpene	4017785	4039933	Sioxanthin_biosynthetic_gene_cluster (80% of genes show similarity)	BGC0001087_c4
Cluster 11	T1pks	4468081	4513783	Dynemicin_biosynthetic_gene_cluster (59% of genes show similarity)	BGC0001060_c1
Cluster 12	T3pks	4669189	4710247	Alkyl-O-Dihydrogeranyl-Methoxyhydroquinones_biosynthetic_gene (71% of genes show similarity)	BGC0001077_c1
Cluster 13	Terpene	6186452	6207402	-	-
Cluster 14	Bacteriocin	6345774	6356598	Lymphostin_biosynthetic_gene_cluster (33% of genes show similarity)	BGC0001006_c1
Cluster 15	Otherks	6508782	6549804	Friulimicin_biosynthetic_gene_cluster (6% of genes show similarity)	BGC0000354_c1

M. chokoriensis DSM 45160^T

Cluster 1	Bacteriocin	565570	576424	-	-
Cluster 2	Siderophore	709510	722733	-	-
Cluster 3	Terpene	1081382	1102305	Sioxanthin_biosynthetic_gene_cluster (80% of genes show similarity)	BGC0001087_c4
Cluster 4	T3pks	1657634	1698680	Alkyl-O-Dihydrogeranyl-Methoxyhydroquinones_biosynthetic_gene (71% of genes show similarity)	BGC0001077_c1
Cluster 5	Terpene	3140424	3161371	-	-
Cluster 6	Bacteriocin	3322910	3333752	Lymphostin_biosynthetic_gene_cluster (33% of genes show similarity)	BGC0001006_c1
Cluster 7	Nrps-T1pks	3565546	3619621	Oxazolomycin_biosynthetic_gene_cluster (9% of genes show similarity)	BGC0001106_c1
Cluster 8	Terpene	3615753	3636673	-	-
Cluster 9	T1pks	3683990	3773103	Streptazone_E_biosynthetic_gene_cluster (75% of genes show similarity)	BGC0001296_c1
Cluster 10	T2pks	3816834	3859325	Actinorhodin_biosynthetic_gene_cluster (95% of genes show similarity)	BGC0000194_c1
Cluster 11	Siderophore	4317542	4329386	Desferrioxamine_B_biosynthetic_gene_cluster (80% of genes show similarity)	BGC0000941_c1
Cluster 12	T2pks	4499651	4542283	Xantholipin_biosynthetic_gene_cluster (14% of genes show similarity)	BGC0000279_c1
Cluster 13	Lantipeptide	4673661	4700696	SapB_biosynthetic_gene_cluster (75% of genes show similarity)	BGC0000551_c1
Cluster 14	Terpene	5011061	5032305	-	-
Cluster 15	T1pks	5294856	5478251	ECO-02301_biosynthetic_gene_cluster (39% of genes show similarity)	BGC0000052_c1
Cluster 16	Lantipeptide	5709383	5732595	Streptomycin_biosynthetic_gene_cluster (12% of genes show similarity)	BGC0000717_c1

M. citrea DSM 43903^{T}

Cluster 1	Terpene	18983	40236	Calcium-dependent_antibiotic_biosynthetic_gene_cluster (5% of genes show similarity)	BGC0000315_c1
Cluster 2	Lantipeptide	98181	126170	-	-
Cluster 3	Nrps	226401	272705	Azicemicin_biosynthetic_gene_cluster (6% of genes show similarity)	BGC0000202_c1
Cluster 4	Thiopeptide-Lantipeptide	438900	472363	Thioviridamide_biosynthetic_gene_cluster (10% of genes show similarity)	BGC0000625_c1
Cluster 5	T2pks	822792	865310	Xantholipin_biosynthetic_gene_cluster (16% of genes show similarity)	BGC0000279_c1
Cluster 6	Nrps-T1pks	2132380	2211003	Sanglifehrin_A_biosynthetic_gene_cluster (9% of genes show similarity)	BGC0001042_c1
Cluster 7	Terpene	3150907	3171944	Phosphonoglycans_biosynthetic_gene_cluster (3% of genes show similarity)	BGC0000806_c1
Cluster 8	Terpene	3251974	3274132	Sioxanthin_biosynthetic_gene_cluster (80% of genes show similarity)	BGC0001087_c4
Cluster 9	T3pks	3930289	3971338	Alkyl-O-Dihydrogeranyl-Methoxyhydroquinones_biosynthetic_gene (71% of genes show similarity)	BGC0001077_c1
Cluster 10	Other	4877415	4921014	-	-
Cluster 11	Terpene	5455336	5476286	-	-
Cluster 12	Bacteriocin	5623355	5634173	Lymphostin_biosynthetic_gene_cluster (30% of genes show similarity)	BGC0001007_c1
Cluster 13	Lantipeptide	6003065	6029150	-	-
Cluster 14	T1pks-Otherks-Oligosacchari	6134997	6216772	Calicheamicin_biosynthetic_gene_cluster (68% of genes show similarity)	BGC0000033_c1
Cluster 15	Siderophore-Nrps-T1pks	6382112	6608242	Rifamycin_biosynthetic_gene_cluster (35% of genes show similarity)	BGC0000137_c1
Cluster 16	Nrps-Lantipeptide-T1pks	6638654	6706455	Bleomycin_biosynthetic_gene_cluster (12% of genes show similarity)	BGC0000963_c1
Cluster 17	Lantipeptide	6876112	6898706	SapB_biosynthetic_gene_cluster (75% of genes show similarity)	BGC0000551_c1

M. coriariae DSM 44875^{T}

Cluster 1	Terpene	2371474	2392436	-	-
Cluster 2	Terpene	2473828	2494748	Sioxanthin_biosynthetic_gene_cluster (80% of genes show similarity)	BGC0001087_c4
Cluster 3	T3pks	3060107	3101156	Alkyl-O-Dihydrogeranyl-Methoxyhydroquinones_biosynthetic_gene (71% of genes show similarity)	BGC0001077_c1
Cluster 4	Terpene	4538535	4559482	-	-
Cluster 5	Bacteriocin-Terpene	4694831	4718037	Lymphostin_biosynthetic_gene_cluster (41% of genes show similarity)	BGC0001006_c1
Cluster 6	Siderophore	5769632	5781479	Desferrioxamine_B_biosynthetic_gene_cluster (83% of genes show similarity)	BGC0000940_c1
Cluster 7	T2pks	5933213	5975731	Xantholipin_biosynthetic_gene_cluster (14% of genes show similarity)	BGC0000279_c1
Cluster 8	Arylpolyene	6131947	6173062	Kedarcidin_biosynthetic_gene_cluster (12% of genes show similarity)	BGC0000081_c1
Cluster 9	Terpene	6497009	6518316	-	-
Cluster 10	Nrps-T1pks-Lantipeptide	6593035	6660725	Bleomycin_biosynthetic_gene_cluster (12% of genes show similarity)	BGC0000963_c1
Cluster 11	Thiopeptide-Lantipeptide	6752299	6781699	Cyclomarin_biosynthetic_gene_cluster (8% of genes show similarity)	BGC0000333_c1

M. coxensis DSM 45161^{T}

Cluster 1	Terpene	302598	323803	-	-
Cluster 2	Lantipeptide	590630	615281	-	-
Cluster 3	Nrps	725601	780533	Nocathiacin_biosynthetic_gene_cluster (4% of genes show similarity)	BGC0000609_c1
Cluster 4	Lantipeptide	832509	855112	SapB_biosynthetic_gene_cluster (100% of genes show similarity)	BGC0000551_c1
Cluster 5	T2pks	1035484	1077999	Pradimicin_biosynthetic_gene_cluster (25% of genes show similarity)	BGC0000256_c1
Cluster 6	Nrps-T1pks	1241158	1298796	Maduropeptin_biosynthetic_gene_cluster (50% of genes show similarity)	BGC0001008_c1
Cluster 7	Terpene-Bacteriocin	1466790	1496728	Lymphostin_biosynthetic_gene_cluster (38% of genes show similarity)	BGC0001007_c1
Cluster 8	Terpene	1634361	1655305	-	-
Cluster 9	T3pks	3118287	3159336	Alkyl-O-Dihydrogeranyl-Methoxyhydroquinones_biosynthetic_gene (71% of genes show similarity)	BGC0001077_c1
Cluster 10	Terpene	3743377	3764294	Sioxanthin_biosynthetic_gene_cluster (100% of genes show similarity)	BGC0001087_c4
Cluster 11	Terpene	3826509	3847474	Phosphonoglycans_biosynthetic_gene_cluster (3% of genes show similarity)	BGC0000806_c1
Cluster 12	Siderophore	5566946	5578730	Desferrioxamine_B_biosynthetic_gene_cluster (80% of genes show similarity)	BGC0000941_c1
Cluster 13	Other	5831286	5873805	Streptomycin_biosynthetic_gene_cluster (8% of genes show similarity)	BGC0000717_c1
Cluster 14	Nrps-Lantipeptide-T1pks	5944742	6012600	Bleomycin_biosynthetic_gene_cluster (15% of genes show similarity)	BGC0000963_c1
Cluster 15	Nrps-T1pks	6178625	6405463	Rifamycin_biosynthetic_gene_cluster (38% of genes show similarity)	BGC0000137_c1
Cluster 16	Nrps	6433336	6491450	Daptomycin_biosynthetic_gene_cluster (3% of genes show similarity)	BGC0000336_c1
Cluster 17	Nrps	6703203	6758490	Ansatrienin_(mycotrienin)_biosynthetic_gene_cluster (11% of genes show similarity)	BGC0000957_c1

M. cremea DSM 45599^{T}

Cluster 1	Nrps-Lantipeptide-T1pks	1143895	1210012	Bleomycin_biosynthetic_gene_cluster (15% of genes show similarity)	BGC0000963_c1
Cluster 2	Terpene	1269796	1291106	-	-
Cluster 3	Siderophore	2102532	2114379	Desferrioxamine_B_biosynthetic_gene_cluster (83% of genes show similarity)	BGC0000940_c1
Cluster 4	Terpene-Bacteriocin	84228	112421	Lymphostin_biosynthetic_gene_cluster (38% of genes show similarity)	BGC0001007_c1
Cluster 5	Terpene	245008	265955	-	-
Cluster 6	T3pks	1703570	1744619	Alkyl-O-Dihydrogeranyl-Methoxyhydroquinones_biosynthetic_gene (71% of genes show similarity)	BGC0001077_c1
Cluster 7	Terpene	2324648	2345565	Sioxanthin_biosynthetic_gene_cluster (100% of genes show similarity)	BGC0001087_c4
Cluster 8	Terpene	2408351	2429358	Phosphonoglycans_biosynthetic_gene_cluster (3% of genes show similarity)	BGC0000806_c1
Cluster 9	Siderophore	2729263	2742483	-	-

M. eburnea DSM 44814^{T}

Cluster 1	Terpene	139707	160735	Sioxanthin_biosynthetic_gene_cluster (80% of genes show similarity)	BGC0001087_c4
Cluster 2	Terpene	222424	243374	Phosphonoglycans_biosynthetic_gene_cluster (3% of genes show similarity)	BGC0000806_c1
Cluster 3	T2pks	2655707	2698210	Xantholipin_biosynthetic_gene_cluster (16% of genes show similarity)	BGC0000279_c1

Cluster 4	Terpene	2858687	2884730	Hopene_biosynthetic_gene_cluster (46% of genes show similarity)	BGC0000663_c1
Cluster 5	T1pks-Nrps	2903211	3036326	Neocarzilin_biosynthetic_gene_cluster (71% of genes show similarity)	BGC0000111_c1
Cluster 6	Siderophore	3064629	3076464	Desferrioxamine_B_biosynthetic_gene_cluster (80% of genes show similarity)	BGC0000941_c1
Cluster 7	T1pks	3104293	3232273	Apoptolidin_biosynthetic_gene_cluster (28% of genes show similarity)	BGC0000021_c1
Cluster 8	Terpene	3617624	3638877	Thiotetroamide_biosynthetic_gene_cluster (11% of genes show similarity)	BGC0001236_c1
Cluster 9	Nrps-T1pks	3823515	3901400	Nostopeptolide_biosynthetic_gene_cluster (37% of genes show similarity)	BGC0001028_c1
Cluster 10	T1pks-Nrps	4003979	4087905	Maduropeptin_biosynthetic_gene_cluster (64% of genes show similarity)	BGC0001008_c1
Cluster 11	Lantipeptide-Nrps	4121621	4199695	Pellasoren_biosynthetic_gene_cluster (41% of genes show similarity)	BGC0001034_c1
Cluster 12	Lantipeptide	4273410	4296295	Blasticidin_biosynthetic_gene_cluster (7% of genes show similarity)	BGC0000874_c1
Cluster 13	Nrps-T1pks-Lantipeptide	4357478	4419263	Microsclerodermins_biosynthetic_gene_cluster (21% of genes show similarity)	BGC0001231_c1
Cluster 14	Lantipeptide-T1pks	4491996	4574969	Tiacumicin_B_biosynthetic_gene_cluster (67% of genes show similarity)	BGC0000165_c1
Cluster 15	T1pks-Otherks	4556484	4602692	Tiacumicin_B_biosynthetic_gene_cluster (64% of genes show similarity)	BGC0000165_c1
Cluster 16	Ladderane-Otherks-T1pks-Ol	4651345	4897818	Chlorothricin_biosynthetic_gene_cluster (60% of genes show similarity)	BGC0000036_c1
Cluster 17	Terpene-Bacteriocin	5090870	5119161	Lymphostin_biosynthetic_gene_cluster (38% of genes show similarity)	BGC0001007_c1
Cluster 18	Terpene	5249671	5270621	-	-
Cluster 19	Ectoine	6511412	6521783	Pristinamycin_biosynthetic_gene_cluster (17% of genes show similarity)	BGC0000952_c2
Cluster 20	T3pks	6731226	6772275	Alkyl-O-Dihydrogeranyl-Methoxyhydroquinones_biosynthetic_gene (71% of genes show similarity)	BGC0001077_c1
M. echinaurantiaca DSM 43904^{T}					
Cluster 1	Terpene	1181030	1201971	-	-
Cluster 2	Bacteriocin	1347341	1358159	Lymphostin_biosynthetic_gene_cluster (30% of genes show similarity)	BGC0001007_c1
Cluster 3	Siderophore	1728180	1739964	Desferrioxamine_B_biosynthetic_gene_cluster (80% of genes show similarity)	BGC0000941_c1
Cluster 4	Other	1777644	1821708	-	-
Cluster 5	Thiopeptide-Lantipeptide	1881255	1907983	-	-
Cluster 6	Nrps	1934052	2000175	Kedarcidin_biosynthetic_gene_cluster (10% of genes show similarity)	BGC0000081_c1
Cluster 7	Nrps	1991099	2050538	Kedarcidin_biosynthetic_gene_cluster (6% of genes show similarity)	BGC0000081_c1
Cluster 8	Terpene	2214616	2235809	-	-
Cluster 9	Nrps	2480656	2529445	Thiolutin_biosynthetic_gene_cluster (8% of genes show similarity)	BGC0001193_c1
Cluster 10	Arylpolyene-T2pks	2723746	2771475	Xantholipin_biosynthetic_gene_cluster (14% of genes show similarity)	BGC0000279_c1
Cluster 11	Nrps-T1pks-Lantipeptide	3406942	3474721	Bleomycin_biosynthetic_gene_cluster (12% of genes show similarity)	BGC0000963_c1
Cluster 12	T3pks	5934023	5975075	Alkyl-O-Dihydrogeranyl-Methoxyhydroquinones_biosynthetic_gene (71% of genes show similarity)	BGC0001077_c1
Cluster 13	Terpene	6589724	6610653	Sioxanthin_biosynthetic_gene_cluster (100% of genes show similarity)	BGC0001087_c4
M. echinofusca DSM 43913^{T}					
Cluster 1	Other	879109	922237	-	-
Cluster 2	Terpene	1514224	1535177	-	-
Cluster 3	Bacteriocin-Terpene	1666938	1690918	Lymphostin_biosynthetic_gene_cluster (38% of genes show similarity)	BGC0001007_c1
Cluster 4	Lantipeptide	2015160	2037898	-	-
Cluster 5	Nrps	2044830	2097635	Mannopeptimycin_biosynthetic_gene_cluster (7% of genes show similarity)	BGC0000388_c1
Cluster 6	Bacteriocin	2124814	2135653	-	-
Cluster 7	Nrps-Lantipeptide	2143436	2256077	Jagaricin_biosynthetic_gene_cluster (13% of genes show similarity)	BGC0001127_c1
Cluster 8	Lantipeptide	2249764	2273063	Calicheamicin_biosynthetic_gene_cluster (2% of genes show similarity)	BGC0000033_c1
Cluster 9	T1pks	2299705	2345527	Sporolide_biosynthetic_gene_cluster (31% of genes show similarity)	BGC0000150_c1
Cluster 10	T2pks	2504857	2547375	Xantholipin_biosynthetic_gene_cluster (16% of genes show similarity)	BGC0000279_c1
Cluster 11	Nrps-T1pks	2694824	2749001	Oxazolomycin_biosynthetic_gene_cluster (9% of genes show similarity)	BGC0001106_c1
Cluster 12	Nrps	2773441	2854050	-	-
Cluster 13	Terpene	2902048	2923307	-	-
Cluster 14	Lantipeptide	3143197	3165797	SapB_biosynthetic_gene_cluster (75% of genes show similarity)	BGC0000551_c1
Cluster 15	T1pks-Nrps-Siderophore	3627511	3877834	Rifamycin_biosynthetic_gene_cluster (35% of genes show similarity)	BGC0000137_c1
Cluster 16	Nrps	3883372	3934758	Tetronasin_biosynthetic_gene_cluster (5% of genes show similarity)	BGC0000163_c1
Cluster 17	Lantipeptide	4398686	4424524	-	-
Cluster 18	Nrps-T1pks	4985155	5104153	Elaiophylin_biosynthetic_gene_cluster (33% of genes show similarity)	BGC0000053_c1
Cluster 19	Other	5087210	5130224	Livipeptin_biosynthetic_gene_cluster (100% of genes show similarity)	BGC0001168_c1
Cluster 20	Nrps	5650460	5714075	Azicemicin_biosynthetic_gene_cluster (11% of genes show similarity)	BGC0000202_c1
Cluster 21	Lantipeptide-T1pks-Nrps	5894124	5962666	Bleomycin_biosynthetic_gene_cluster (6% of genes show similarity)	BGC0000963_c1
Cluster 22	Terpene	6056696	6077679	Phosphonoglycans_biosynthetic_gene_cluster (3% of genes show similarity)	BGC0000806_c1
Cluster 23	Nrps	6118168	6171254	Meilingmycin_biosynthetic_gene_cluster (3% of genes show similarity)	BGC0000093_c1
Cluster 24	T1pks	6179310	6224637	Neocarzinostatin_biosynthetic_gene_cluster (6% of genes show similarity)	BGC0000112_c1
Cluster 25	Terpene	6228787	6249725	Sioxanthin_biosynthetic_gene_cluster (100% of genes show similarity)	BGC0001087_c4
Cluster 26	Nrps-T1pks	6661327	6728662	Kedarcidin_biosynthetic_gene_cluster (25% of genes show similarity)	BGC0000081_c1
Cluster 27	T3pks-Thiopeptide-Lantipept	6906359	6960767	Alkyl-O-Dihydrogeranyl-Methoxyhydroquinones_biosynthetic_gene (71% of genes show similarity)	BGC0001077_c1
M. echinospora DSM 1040					
Cluster 1	Nrps	38956	93408	Thiolutin_biosynthetic_gene_cluster (12% of genes show similarity)	BGC0001193_c1
Cluster 2	T1pks-Otherks	91976	144355	-	-
Cluster 3	Bacteriocin	148412	160298	-	-
Cluster 4	Nrps	176647	233915	Gobichelin_biosynthetic_gene_cluster (44% of genes show similarity)	BGC0000366_c1
Cluster 5	T2pks	421235	463765	Spore_pigment_biosynthetic_gene_cluster (41% of genes show similarity)	BGC0000271_c1
Cluster 6	Nrps-T1pks	503541	558122	Laspartomycin_biosynthetic_gene_cluster (6% of genes show similarity)	BGC0000379_c1
Cluster 7	Nrps	547082	598609	Fosfomycin_biosynthetic_gene_cluster (10% of genes show similarity)	BGC0000938_c1
Cluster 8	Lassopeptide	597042	619577	-	-
Cluster 9	Otherks-Nrps	607294	662175	Kedarcidin_biosynthetic_gene_cluster (4% of genes show similarity)	BGC0000081_c1
Cluster 10	Nrps-T3pks	737129	789358	Feglymycin_biosynthetic_gene_cluster (47% of genes show similarity)	BGC0001233_c1
Cluster 11	T3pks-T1pks-Nrps	795257	905003	Crocacin_biosynthetic_gene_cluster (38% of genes show similarity)	BGC0000974_c1
Cluster 12	Nrps-T1pks-Lantipeptide	1239272	1304642	Bleomycin_biosynthetic_gene_cluster (15% of genes show similarity)	BGC0000963_c1
Cluster 13	Nrps	1290459	1336759	Sibiromycin_biosynthetic_gene_cluster (84% of genes show similarity)	BGC0000428_c1
Cluster 14	T2pks-Otherks-T1pks	1635096	1694800	TLN-05220_biosynthetic_gene_cluster (86% of genes show similarity)	BGC0001062_c1
Cluster 15	Nrps	1839438	1897319	Syringopeptin_biosynthetic_gene_cluster (66% of genes show similarity)	BGC0000438_c1
Cluster 16	T1pks	3069406	3121848	Azinomycin_B_biosynthetic_gene_cluster (4% of genes show similarity)	BGC0000960_c1
Cluster 17	Lantipeptide-Fused	3282393	3315979	Pheganomycin_biosynthetic_gene_cluster (19% of genes show similarity)	BGC0001148_c1
Cluster 18	Siderophore	3331649	3343520	Desferrioxamine_B_biosynthetic_gene_cluster (80% of genes show similarity)	BGC0000941_c1
Cluster 19	Terpene	3548427	3569383	-	-
Cluster 20	Terpene	3635131	3656072	Sioxanthin_biosynthetic_gene_cluster (100% of genes show similarity)	BGC0001087_c4
Cluster 21	Lantipeptide	4058278	4083040	Gentamicin_biosynthetic_gene_cluster (18% of genes show similarity)	BGC0000696_c1
Cluster 22	Amglyccycl	4112207	4133400	Gentamicin_biosynthetic_gene_cluster (27% of genes show similarity)	BGC0000696_c1
Cluster 23	T3pks	4299095	4340144	Alkyl-O-Dihydrogeranyl-Methoxyhydroquinones_biosynthetic_gene (57% of genes show similarity)	BGC0001077_c1

Cluster 24	Bacteriocin	26797	38122	-	-
Cluster 25	Nrps-T1pks-Lantipeptide-Nuc	184698	252522	Muraymycin_biosynthetic_gene_cluster (18% of genes show similarity)	BGC0001020_c1
Cluster 26	Lantipeptide-Nrps	245342	299313	Maklamicin_biosynthetic_gene_cluster (4% of genes show similarity)	BGC0001288_c1
Cluster 27	T1pks-Nrps	292788	536302	Macbecin_biosynthetic_gene_cluster (43% of genes show similarity)	BGC0000090_c1
Cluster 28	T1pks	568209	618165	Tetronasin_biosynthetic_gene_cluster (9% of genes show similarity)	BGC0000163_c1
Cluster 29	Nrps	1231897	1277284	-	-
Cluster 30	Terpene-Bacteriocin	1317947	1346480	Lymphostin_biosynthetic_gene_cluster (30% of genes show similarity)	BGC0001007_c1
Cluster 31	Terpene	1491419	1512369	-	-
Cluster 32	Nrps	1726171	1771001	Tetronasin_biosynthetic_gene_cluster (3% of genes show similarity)	BGC0000163_c1
Cluster 33	T3pks	1879464	1920540	Herbimycin_biosynthetic_gene_cluster (10% of genes show similarity)	BGC0000074_c1
Cluster 34	Other	2095492	2138644	-	-
M. echinospora DSM 43816^T					
Cluster 1	Nrps	155307	213188	-	-
Cluster 2	T1pks	1438820	1491265	Azinomycin_B_biosynthetic_gene_cluster (4% of genes show similarity)	BGC0000960_c1
Cluster 3	Siderophore	1600797	1612647	Desferrioxamine_B_biosynthetic_gene_cluster (80% of genes show similarity)	BGC0000941_c1
Cluster 4	Terpene	1823139	1844095	-	-
Cluster 5	Terpene	1918345	1939265	Sioxanthin_biosynthetic_gene_cluster (100% of genes show similarity)	BGC0001087_c4
Cluster 6	Lantipeptide	2382382	2406944	-	-
Cluster 7	Lantipeptide	2470538	2495300	Gentamicin_biosynthetic_gene_cluster (21% of genes show similarity)	BGC0000696_c1
Cluster 8	Amglyccycl	2519709	2540902	Gentamicin_biosynthetic_gene_cluster (27% of genes show similarity)	BGC0000696_c1
Cluster 9	T3pks	2703701	2744750	Alkyl-O-Dihydrogeranyl-Methoxyhydroquinones_biosynthetic_gene (57% of genes show similarity)	BGC0001077_c1
Cluster 10	Other	3683128	3726757	-	-
Cluster 11	T3pks	3917047	3958123	Herbimycin_biosynthetic_gene_cluster (10% of genes show similarity)	BGC0000074_c1
Cluster 12	Indole	3970976	3992166	Pyoluteorin_biosynthetic_gene_cluster (15% of genes show similarity)	BGC0000128_c1
Cluster 13	Terpene	4329096	4349995	-	-
Cluster 14	Bacteriocin-Terpene	4515751	4539312	Lymphostin_biosynthetic_gene_cluster (30% of genes show similarity)	BGC0001007_c1
Cluster 15	Nrps-T1pks	4580437	4631653	-	-
Cluster 16	Nrps-Lantipeptide	4729221	4815787	Hormaomycin_biosynthetic_gene_cluster (17% of genes show similarity)	BGC0000374_c1
Cluster 17	T1pks	5369730	5419716	Herboxidiene_biosynthetic_gene_cluster (5% of genes show similarity)	BGC0001065_c1
Cluster 18	Nrps-T1pks-Lantipeptide	5450229	5761561	Rifamycin_biosynthetic_gene_cluster (38% of genes show similarity)	BGC0000137_c1
Cluster 19	Nucleoside	5764671	5785033	Fluostatin_biosynthetic_gene_cluster (3% of genes show similarity)	BGC0000223_c1
Cluster 20	Lantipeptide-Bacteriocin	5895050	5930239	SapB_biosynthetic_gene_cluster (75% of genes show similarity)	BGC0000551_c1
Cluster 21	Nrps	6001617	6055994	Thiolutin_biosynthetic_gene_cluster (12% of genes show similarity)	BGC0001193_c1
Cluster 22	T1pks-Otherks	6059504	6111916	-	-
Cluster 23	Bacteriocin	6110101	6121987	-	-
Cluster 24	Nrps	6140152	6197500	Gobichelin_biosynthetic_gene_cluster (33% of genes show similarity)	BGC0000366_c1
Cluster 25	Nrps	6383572	6430210	Pristinamycin_biosynthetic_gene_cluster (7% of genes show similarity)	BGC0000952_c1
Cluster 26	T2pks	6421413	6463940	Spore_pigment_biosynthetic_gene_cluster (41% of genes show similarity)	BGC0000271_c1
Cluster 27	Nrps-T1pks	6502620	6557903	Laspartomycin_biosynthetic_gene_cluster (6% of genes show similarity)	BGC0000379_c1
Cluster 28	Nrps	6548275	6599605	Fosfomycin_biosynthetic_gene_cluster (13% of genes show similarity)	BGC0000938_c1
Cluster 29	Lassopeptide	6598730	6621265	-	-
Cluster 30	Otherks-Nrps	6608960	6664363	Kedarcidin_biosynthetic_gene_cluster (4% of genes show similarity)	BGC0000081_c1
Cluster 31	Nrps-T3pks	6742388	6794626	Feglymycin_biosynthetic_gene_cluster (47% of genes show similarity)	BGC0001233_c1
Cluster 32	T3pks-T1pks-Nrps	6800633	6913222	Crocacin_biosynthetic_gene_cluster (38% of genes show similarity)	BGC0000974_c1
Cluster 33	Nrps-T1pks-Lantipeptide	7241848	7307282	Bleomycin_biosynthetic_gene_cluster (15% of genes show similarity)	BGC0000963_c1
Cluster 34	Nrps	7292122	7338423	Sibiromycin_biosynthetic_gene_cluster (84% of genes show similarity)	BGC0000428_c1
Cluster 35	T2pks-Otherks-T1pks	7669817	7729353	TLN-05220_biosynthetic_gene_cluster (83% of genes show similarity)	BGC0001062_c1
M. endolithica DSM 44398^T					
Cluster 1	Terpene	604801	625742	-	-
Cluster 2	Bacteriocin	776591	787409	Lymphostin_biosynthetic_gene_cluster (30% of genes show similarity)	BGC0001007_c1
Cluster 3	Otherks-T1pks-Nrps	1277400	1411869	Chlorothricin_biosynthetic_gene_cluster (48% of genes show similarity)	BGC0000036_c1
Cluster 4	Siderophore	1736123	1754019	Desferrioxamine_B_biosynthetic_gene_cluster (80% of genes show similarity)	BGC0000941_c1
Cluster 5	Bacteriocin	1809409	1819759	-	-
Cluster 6	T2pks-Arylpolyene	1976182	2023561	Xantholipin_biosynthetic_gene_cluster (14% of genes show similarity)	BGC0000279_c1
Cluster 7	Lantipeptide	2277607	2300210	SapB_biosynthetic_gene_cluster (100% of genes show similarity)	BGC0000551_c1
Cluster 8	Terpene	2521877	2543133	Meilingmycin_biosynthetic_gene_cluster (2% of genes show similarity)	BGC0000093_c1
Cluster 9	Nrps-T1pks-Linaridin	3363322	3454009	Pristinamycin_biosynthetic_gene_cluster (7% of genes show similarity)	BGC0000952_c1
Cluster 10	Terpene	5126046	5147053	Phosphonoglycans_biosynthetic_gene_cluster (3% of genes show similarity)	BGC0000806_c1
Cluster 11	Terpene	5207401	5228312	Sioxanthin_biosynthetic_gene_cluster (80% of genes show similarity)	BGC0001087_c4
Cluster 12	T3pks	6074143	6115192	Alkyl-O-Dihydrogeranyl-Methoxyhydroquinones_biosynthetic_gene (71% of genes show similarity)	BGC0001077_c1
M. haikouensis DSM 45626^T					
Cluster 1	Nrps	11788	76848	Gobichelin_biosynthetic_gene_cluster (16% of genes show similarity)	BGC0000366_c1
Cluster 2	Terpene	94741	115919	-	-
Cluster 3	Siderophore	592	11602	-	-
Cluster 4	Lantipeptide	18051	40693	SapB_biosynthetic_gene_cluster (75% of genes show similarity)	BGC0000551_c1
Cluster 5	Amglyccycl	60368	81648	Validamycin_biosynthetic_gene_cluster (22% of genes show similarity)	BGC0000722_c1
Cluster 6	Other	219615	247431	-	-
Cluster 7	T3pks	102172	143221	Alkyl-O-Dihydrogeranyl-Methoxyhydroquinones_biosynthetic_gene (57% of genes show similarity)	BGC0001077_c1
Cluster 8	Terpene	121817	142764	-	-
Cluster 9	Bacteriocin	46963	57787	Lymphostin_biosynthetic_gene_cluster (30% of genes show similarity)	BGC0001007_c1
Cluster 10	Terpene	139107	160120	-	-
Cluster 11	Oligosaccharide	23439	45340	-	-
Cluster 12	T2pks	81030	123644	Spore_pigment_biosynthetic_gene_cluster (50% of genes show similarity)	BGC0000271_c1
Cluster 13	Nrps-Lantipeptide-T1pks	120644	177392	Bleomycin_biosynthetic_gene_cluster (12% of genes show similarity)	BGC0000963_c1
Cluster 14	Arylpolyene	12998	54392	Hygromycin_A_biosynthetic_gene_cluster (24% of genes show similarity)	BGC0000698_c1
Cluster 15	Terpene	31209	52132	Sioxanthin_biosynthetic_gene_cluster (80% of genes show similarity)	BGC0001087_c4
Cluster 16	T1pks	88625	151228	ECO-02301_biosynthetic_gene_cluster (42% of genes show similarity)	BGC0000052_c1
Cluster 17	Nrps	13387	60745	-	-
Cluster 18	Nrps-T1pks	55001	106999	Virginiamycin_biosynthetic_gene_cluster (11% of genes show similarity)	BGC0001116_c1
Cluster 19	Nrps-T1pks-Siderophore	5074	134880	Tallysomycin_biosynthetic_gene_cluster (40% of genes show similarity)	BGC0001048_c1
Cluster 20	Nrps	7447	70191	Leinamycin_biosynthetic_gene_cluster (5% of genes show similarity)	BGC0001101_c1
Cluster 21	Nrps-T1pks-Aminocoumarin-	1	71072	Rubradirin_biosynthetic_gene_cluster (18% of genes show similarity)	BGC0000141_c1
Cluster 22	T1pks	1	41386	Kosinostatin_biosynthetic_gene_cluster (22% of genes show similarity)	BGC0001073_c1

Cluster 23	Nrps	65463	103145	Gentamicin_biosynthetic_gene_cluster (4% of genes show similarity)	BGC0000696_c1
Cluster 24	Amglyccycl	34384	55565	-	-
Cluster 25	T1pks	55909	98971	KS-505a_biosynthetic_gene_cluster (7% of genes show similarity)	BGC0000651_c1
Cluster 26	T1pks-Otherks	6154	52541	Tetrocarcin_A_biosynthetic_gene_cluster (20% of genes show similarity)	BGC0000162_c1
Cluster 27	Bacteriocin	22125	34062	-	-
Cluster 28	T1pks	4165	49987	Sporolide_biosynthetic_gene_cluster (23% of genes show similarity)	BGC0000150_c1
Cluster 29	Nrps	14613	60128	Asukamycin_biosynthetic_gene_cluster (6% of genes show similarity)	BGC0000187_c1
Cluster 30	Nrps	12021	45478	Albachelin_biosynthetic_gene_cluster (70% of genes show similarity)	BGC0001211_c1
Cluster 31	T1pks	1	21794	-	-
Cluster 32	T1pks	1	42683	Ansamitocin_biosynthetic_gene_cluster (9% of genes show similarity)	BGC0000020_c1
Cluster 33	Lassopeptide	500	22297	-	-
Cluster 34	T1pks	1	37643	E-837_biosynthetic_gene_cluster (62% of genes show similarity)	BGC0000050_c1
Cluster 35	Lantipeptide	3774	36421	-	-
Cluster 36	T1pks	1	15747	ECO-02301_biosynthetic_gene_cluster (35% of genes show similarity)	BGC0000052_c1
Cluster 37	T1pks	1	13186	ECO-02301_biosynthetic_gene_cluster (32% of genes show similarity)	BGC0000052_c1
Cluster 38	T1pks	1	12594	Rifamycin_biosynthetic_gene_cluster (28% of genes show similarity)	BGC0000137_c1
Cluster 39	T1pks	1	8076	-	-
Cluster 40	T1pks	1	5376	Micromonolactam_biosynthetic_gene_cluster (100% of genes show similarity)	BGC0000095_c5
Cluster 41	Nrps	1	1796	-	-

M. halophytica DSM 43171^T					
Cluster 1	Terpene	123277	144221	-	-
Cluster 2	T2pks	159535	202050	Xantholipin_biosynthetic_gene_cluster (16% of genes show similarity)	BGC0000279_c1
Cluster 3	T1pks	328329	361828	Rifamycin_biosynthetic_gene_cluster (56% of genes show similarity)	BGC0000137_c1
Cluster 4	Nrps-Lantipeptide-T1pks	51981	119614	Bleomycin_biosynthetic_gene_cluster (15% of genes show similarity)	BGC0000963_c1
Cluster 5	Terpene	11891	33096	-	-
Cluster 6	Terpene-Bacteriocin	43782	73645	Lymphostin_biosynthetic_gene_cluster (38% of genes show similarity)	BGC0001007_c1
Cluster 7	Nrps-T1pks	159622	231516	Caerulomycin_A_biosynthetic_gene_cluster (8% of genes show similarity)	BGC0000966_c1
Cluster 8	T3pks	151373	192422	Alkyl-O-Dihydrogeranyl-Methoxyhydroquinones_biosynthetic_gene (71% of genes show similarity)	BGC0001077_c1
Cluster 9	T1pks	1	136304	Midecamycin_biosynthetic_gene_cluster (11% of genes show similarity)	BGC0000096_c1
Cluster 10	T2pks	143767	186240	Arimetamycin_biosynthetic_gene_cluster (70% of genes show similarity)	BGC0000199_c1
Cluster 11	T1pks	1	53164	Rifamycin_biosynthetic_gene_cluster (25% of genes show similarity)	BGC0000137_c1
Cluster 12	T1pks	40410	86232	Sporolide_biosynthetic_gene_cluster (21% of genes show similarity)	BGC0000150_c1
Cluster 13	Arylpolyene	102439	143827	Hygromycin_A_biosynthetic_gene_cluster (20% of genes show similarity)	BGC0000698_c1
Cluster 14	T1pks	128275	187964	Lomaiviticin_biosynthetic_gene_cluster (9% of genes show similarity)	BGC0000240_c1
Cluster 15	Siderophore	135851	146725	Desferrioxamine_B_biosynthetic_gene_cluster (80% of genes show similarity)	BGC0000941_c1
Cluster 16	Other	3646	46183	Streptomycin_biosynthetic_gene_cluster (8% of genes show similarity)	BGC0000717_c1
Cluster 17	Lantipeptide	46724	68721	Diazepinomicin_biosynthetic_gene_cluster (5% of genes show similarity)	BGC0000679_c1
Cluster 18	Terpene	15573	36490	Sioxanthin_biosynthetic_gene_cluster (100% of genes show similarity)	BGC0001087_c4
Cluster 19	Nrps	13591	52822	Azinomycin_B_biosynthetic_gene_cluster (12% of genes show similarity)	BGC0000960_c1
Cluster 20	Terpene	8746	39719	Phosphonoglycans_biosynthetic_gene_cluster (3% of genes show similarity)	BGC0000806_c1
Cluster 21	Lantipeptide	13584	33619	SapB_biosynthetic_gene_cluster (100% of genes show similarity)	BGC0000551_c1
Cluster 22	T1pks	1	32182	Divergolide_biosynthetic_gene_cluster (24% of genes show similarity)	BGC0001119_c1
Cluster 23	T1pks-Nrps	1	20398	Sanglifehrin_A_biosynthetic_gene_cluster (9% of genes show similarity)	BGC0001042_c1
Cluster 24	T1pks	1	12470	-	-
Cluster 25	T1pks	1	11293	ECO-02301_biosynthetic_gene_cluster (32% of genes show similarity)	BGC0000052_c1
Cluster 26	T1pks	1	9880	Concanamycin_A_biosynthetic_gene_cluster (25% of genes show similarity)	BGC0000040_c1
Cluster 27	T1pks	1	8753	Lobosamide_biosynthetic_gene_cluster (13% of genes show similarity)	BGC0001303_c1
Cluster 28	Nrps	1	1392	-	-

M. humi DSM 45647^T					
Cluster 1	Terpene	6315	28015	Brasilicardin_A_biosynthetic_gene_cluster (27% of genes show similarity)	BGC0000632_c1
Cluster 2	Siderophore	153124	164896	Desferrioxamine_B_biosynthetic_gene_cluster (80% of genes show similarity)	BGC0000941_c1
Cluster 3	Nrps	164922	218269	Azicemicin_biosynthetic_gene_cluster (13% of genes show similarity)	BGC0000202_c1
Cluster 4	T2pks	395333	437848	Xantholipin_biosynthetic_gene_cluster (16% of genes show similarity)	BGC0000279_c1
Cluster 5	Terpene	644744	664912	Nocathiacin_biosynthetic_gene_cluster (4% of genes show similarity)	BGC0000609_c1
Cluster 6	Arylpolyene-Nrps	74290	160369	Kedarcidin_biosynthetic_gene_cluster (13% of genes show similarity)	BGC0000081_c1
Cluster 7	Lantipeptide	400477	423089	SapB_biosynthetic_gene_cluster (100% of genes show similarity)	BGC0000551_c1
Cluster 8	Terpene	393270	414220	-	-
Cluster 9	Terpene	428561	449487	Sioxanthin_biosynthetic_gene_cluster (100% of genes show similarity)	BGC0001087_c4
Cluster 10	Butyrolactone-Lassopeptide	413	22345	-	-
Cluster 11	Terpene	257341	278306	-	-
Cluster 12	T1pks	1	92039	Stambomycin_biosynthetic_gene_cluster (52% of genes show similarity)	BGC0000151_c1
Cluster 13	Nrps-Lantipeptide-T1pks	226355	294096	Bleomycin_biosynthetic_gene_cluster (15% of genes show similarity)	BGC0000963_c1
Cluster 14	T3pks	154375	185685	Alkyl-O-Dihydrogeranyl-Methoxyhydroquinones_biosynthetic_gene (71% of genes show similarity)	BGC0001077_c1
Cluster 15	T1pks	77311	118506	Mycolactone_biosynthetic_gene_cluster (33% of genes show similarity)	BGC0000103_c1
Cluster 16	Nrps-T1pks	1	56404	Bleomycin_biosynthetic_gene_cluster (6% of genes show similarity)	BGC0000963_c1
Cluster 17	Terpene-Bacteriocin	18933	48580	Lymphostin_biosynthetic_gene_cluster (33% of genes show similarity)	BGC0001006_c1
Cluster 18	T1pks	1	26084	Stambomycin_biosynthetic_gene_cluster (32% of genes show similarity)	BGC0000151_c1
Cluster 19	Terpene	1	16214	KS-505a_biosynthetic_gene_cluster (14% of genes show similarity)	BGC0000651_c1
Cluster 20	T1pks	1	16086	-	-
Cluster 21	T1pks	1	14764	Aculeximycin_biosynthetic_gene_cluster (23% of genes show similarity)	BGC0000002_c1

M. inositola DSM 43819^T					
Cluster 1	Terpene	1595662	1616627	-	-
Cluster 2	Terpene	1679924	1700850	Isorenieratene_biosynthetic_gene_cluster (28% of genes show similarity)	BGC0000664_c1
Cluster 3	T3pks	2267753	2308856	Alkyl-O-Dihydrogeranyl-Methoxyhydroquinones_biosynthetic_gene (71% of genes show similarity)	BGC0001077_c1
Cluster 4	Terpene	3798917	3819867	-	-
Cluster 5	Bacteriocin-Terpene	3948210	3973866	Lymphostin_biosynthetic_gene_cluster (38% of genes show similarity)	BGC0001007_c1
Cluster 6	T2pks	4460541	4503106	Xantholipin_biosynthetic_gene_cluster (16% of genes show similarity)	BGC0000279_c1
Cluster 7	Terpene	5011431	5032690	-	-

M. inyonensis DSM 46123^T					
Cluster 1	T3pks	942617	983666	Alkyl-O-Dihydrogeranyl-Methoxyhydroquinones_biosynthetic_gene (57% of genes show similarity)	BGC0001077_c1
Cluster 2	Amglyccycl	1189982	1211166	Gentamicin_biosynthetic_gene_cluster (30% of genes show similarity)	BGC0000696_c1

Cluster 3	Nrps-T1pks	1246535	1306732	-	-
Cluster 4	Terpene	1700161	1721084	Sioxanthin_biosynthetic_gene_cluster (100% of genes show similarity)	BGC0001087_c4
Cluster 5	Terpene	1821257	1842213	Phosphonoglycans_biosynthetic_gene_cluster (3% of genes show similarity)	BGC0000806_c1
Cluster 6	T1pks	1953968	2001482	-	-
Cluster 7	Siderophore	2112669	2124528	Desferrioxamine_B_biosynthetic_gene_cluster (80% of genes show similarity)	BGC0000941_c1
Cluster 8	Lantipeptide	2334982	2359551	-	-
Cluster 9	Arylpolyene	2361889	2403286	Hygromycin_A_biosynthetic_gene_cluster (10% of genes show similarity)	BGC0000698_c1
Cluster 10	T1pks	2458858	2511318	KS-505a_biosynthetic_gene_cluster (7% of genes show similarity)	BGC0000651_c1
Cluster 11	Other	108464	149354	Diazepinomicin_biosynthetic_gene_cluster (67% of genes show similarity)	BGC0000679_c1
Cluster 12	Nrps	260768	307391	-	-
Cluster 13	T3pks	293087	334163	Herbimycin_biosynthetic_gene_cluster (10% of genes show similarity)	BGC0000074_c1
Cluster 14	Nrps	589917	638614	-	-
Cluster 15	Terpene	714373	735323	-	-
Cluster 16	Bacteriocin	884129	894992	Lymphostin_biosynthetic_gene_cluster (23% of genes show similarity)	BGC0001007_c1
Cluster 17	Lantipeptide	1030532	1058673	-	-
Cluster 18	Nrps	1223027	1320490	Gobichelin_biosynthetic_gene_cluster (55% of genes show similarity)	BGC0000366_c1
Cluster 19	Otherks-T1pks	1341140	1393480	-	-
Cluster 20	Nrps-T1pks-T3pks	1660176	1756559	Feglymycin_biosynthetic_gene_cluster (47% of genes show similarity)	BGC0001233_c1
Cluster 21	Lantipeptide	1788780	1825597	SapB_biosynthetic_gene_cluster (75% of genes show similarity)	BGC0000551_c1
Cluster 22	T2pks	2076554	2119081	Xantholipin_biosynthetic_gene_cluster (10% of genes show similarity)	BGC0000279_c1
Cluster 23	Nrps-T1pks	2118295	2173575	Meilingmycin_biosynthetic_gene_cluster (2% of genes show similarity)	BGC0000093_c1
Cluster 24	T1pks-Nucleoside	2279675	2323641	Delftibactin_biosynthetic_gene_cluster (28% of genes show similarity)	BGC0000984_c1
Cluster 25	Nrps-T1pks-Lantipeptide	2498419	2563847	Bleomycin_biosynthetic_gene_cluster (15% of genes show similarity)	BGC0000963_c1
Cluster 26	T2pks-Otherks-T1pks	2945570	3005182	TLN-05220_biosynthetic_gene_cluster (86% of genes show similarity)	BGC0001062_c1
Cluster 27	Terpene	3075053	3096126	-	-
Cluster 28	Nrps	3209940	3267788	-	-
M. krabiensis DSM 45344^T					
Cluster 1	Nrps	103240	161895	Daptomycin_biosynthetic_gene_cluster (4% of genes show similarity)	BGC0000336_c1
Cluster 2	Thiopeptide-Lantipeptide	255102	289387	-	-
Cluster 3	Lantipeptide	617439	643427	-	-
Cluster 4	Nrps	1256215	1305049	Thiolutin_biosynthetic_gene_cluster (8% of genes show similarity)	BGC0001193_c1
Cluster 5	T2pks	1440910	1483431	Xantholipin_biosynthetic_gene_cluster (14% of genes show similarity)	BGC0000279_c1
Cluster 6	Siderophore	1589595	1601409	Desferrioxamine_B_biosynthetic_gene_cluster (83% of genes show similarity)	BGC0000940_c1
Cluster 7	Terpene	1618117	1639349	-	-
Cluster 8	Terpene	1662466	1688536	Hopene_biosynthetic_gene_cluster (53% of genes show similarity)	BGC0000663_c1
Cluster 9	Siderophore	1704242	1717444	-	-
Cluster 10	Nrps-T1pks-Lantipeptide	2147722	2222666	Laspartomycin_biosynthetic_gene_cluster (9% of genes show similarity)	BGC0000379_c1
Cluster 11	Lantipeptide	4075552	4099590	-	-
Cluster 12	Terpene	4506883	4527845	-	-
Cluster 13	Terpene	4598090	4619019	Sioxanthin_biosynthetic_gene_cluster (100% of genes show similarity)	BGC0001087_c4
Cluster 14	T3pks	5174496	5215545	Alkyl-O-Dihydrogeranyl-Methoxyhydroquinones_biosynthetic_gene (71% of genes show similarity)	BGC0001077_c1
Cluster 15	Terpene	6634900	6655844	-	-
Cluster 16	Bacteriocin-Terpene	6792741	6818716	Lymphostin_biosynthetic_gene_cluster (38% of genes show similarity)	BGC0001007_c1
M. lupini Lupac 08					
Cluster 1	Terpene	1	17227	Sioxanthin_biosynthetic_gene_cluster (100% of genes show similarity)	BGC0001087_c4
Cluster 2	T1pks	90982	136750	Sporolide_biosynthetic_gene_cluster (38% of genes show similarity)	BGC0000150_c1
Cluster 3	T2pks-Otherks	19662	64442	Rishirilide_B_biosynthetic_gene_cluster (89% of genes show similarity)	BGC0001179_c1
Cluster 4	Other	76960	120850	-	-
Cluster 5	T2pks	1	25818	Pradimicin_biosynthetic_gene_cluster (25% of genes show similarity)	BGC0000256_c1
Cluster 6	Terpene	32165	51004	Phosphonoglycans_biosynthetic_gene_cluster (3% of genes show similarity)	BGC0000806_c1
Cluster 7	Bacteriocin	173994	184812	Lymphostin_biosynthetic_gene_cluster (33% of genes show similarity)	BGC0001006_c1
Cluster 8	Terpene	340637	361515	-	-
Cluster 9	Nrps	244431	308603	Pacidamycin_biosynthetic_gene_cluster (18% of genes show similarity)	BGC0000951_c1
Cluster 10	T3pks	67075	98000	Alkyl-O-Dihydrogeranyl-Methoxyhydroquinones_biosynthetic_gene (57% of genes show similarity)	BGC0001077_c1
Cluster 11	Butyrolactone-Otherks	61911	85742	SF2575_biosynthetic_gene_cluster (6% of genes show similarity)	BGC0000269_c1
Cluster 12	Terpene	37910	59181	-	-
Cluster 13	Nrps-Otherks-T1pks	143379	213871	Naphthyridinomycin_biosynthetic_gene_cluster (17% of genes show similarity)	BGC0000394_c1
Cluster 14	Lantipeptide	296163	318760	Catenulipeptin_biosynthetic_gene_cluster (60% of genes show similarity)	BGC0000501_c1
Cluster 15	Nrps-Arylpolyene	12524	98389	Kedarcidin_biosynthetic_gene_cluster (13% of genes show similarity)	BGC0000081_c1
Cluster 16	Siderophore	200327	212195	Desferrioxamine_B_biosynthetic_gene_cluster (83% of genes show similarity)	BGC0000940_c1
M. marina DSM 45555^T					
Cluster 1	Terpene	23648	44574	Sioxanthin_biosynthetic_gene_cluster (80% of genes show similarity)	BGC0001087_c4
Cluster 2	Siderophore-Transatpks-Nrps	148010	241946	Leinamycin_biosynthetic_gene_cluster (15% of genes show similarity)	BGC0001101_c1
Cluster 3	Oligosaccharide-Nrps-Terpen	241031	290035	Lobosamide_biosynthetic_gene_cluster (10% of genes show similarity)	BGC0001303_c1
Cluster 4	T2pks	386124	422500	Xantholipin_biosynthetic_gene_cluster (16% of genes show similarity)	BGC0000279_c1
Cluster 5	Lantipeptide	161496	192605	Pentalenolactone_biosynthetic_gene_cluster (15% of genes show similarity)	BGC0000678_c1
Cluster 6	Terpene	239133	260335	Kiamycin_biosynthetic_gene_cluster (10% of genes show similarity)	BGC0000235_c1
Cluster 7	Terpene	72978	93928	-	-
Cluster 8	Terpene	34838	55803	Phosphonoglycans_biosynthetic_gene_cluster (3% of genes show similarity)	BGC0000806_c1
Cluster 9	T3pks	125450	156604	Alkyl-O-Dihydrogeranyl-Methoxyhydroquinones_biosynthetic_gene (71% of genes show similarity)	BGC0001077_c1
Cluster 10	T1pks	1	85449	Concanamycin_A_biosynthetic_gene_cluster (25% of genes show similarity)	BGC0000040_c1
Cluster 11	Terpene-Bacteriocin	80719	110283	Lymphostin_biosynthetic_gene_cluster (38% of genes show similarity)	BGC0001007_c1
Cluster 12	Otherks-Lantipeptide-Nrps-T	346	112480	Azicemicin_biosynthetic_gene_cluster (13% of genes show similarity)	BGC0000202_c1
Cluster 13	Nrps-T1pks	105161	141575	Bleomycin_biosynthetic_gene_cluster (6% of genes show similarity)	BGC0000963_c1
Cluster 14	Nrps-T1pks	1	47446	Rifamycin_biosynthetic_gene_cluster (35% of genes show similarity)	BGC0000137_c1
Cluster 15	T2pks	45641	88147	Actinorhodin_biosynthetic_gene_cluster (54% of genes show similarity)	BGC0000194_c1
Cluster 16	Nrps	10956	64575	Coelibactin_biosynthetic_gene_cluster (63% of genes show similarity)	BGC0000324_c1
Cluster 17	Nrps-T1pks-Lantipeptide	1	67515	Bleomycin_biosynthetic_gene_cluster (12% of genes show similarity)	BGC0000963_c1
Cluster 18	T1pks	1	32328	Calicheamicin_biosynthetic_gene_cluster (13% of genes show similarity)	BGC0000033_c1
Cluster 19	Nrps	1	40492	Lobosamide_biosynthetic_gene_cluster (6% of genes show similarity)	BGC0001303_c1
Cluster 20	Nrps-Lantipeptide	1	29732	Zorbamycin_biosynthetic_gene_cluster (6% of genes show similarity)	BGC0001058_c1
Cluster 21	T1pks	1	22129	Tautomycin_biosynthetic_gene_cluster (10% of genes show similarity)	BGC0000159_c1
Cluster 22	Lantipeptide	1	15163	-	-

Cluster 23	T1pks	1	13246	ECO-02301_biosynthetic_gene_cluster (35% of genes show similarity)	BGC0000052_c1
Cluster 24	T1pks	1	10509	ECO-02301_biosynthetic_gene_cluster (28% of genes show similarity)	BGC0000052_c1
Cluster 25	T1pks	1	9588	ECO-02301_biosynthetic_gene_cluster (32% of genes show similarity)	BGC0000052_c1
Cluster 26	T1pks	1	8332	-	-
Cluster 27	T1pks	1	6415	-	-
Cluster 28	T1pks	1	6207	-	-
Cluster 29	Nrps	1	3259	-	-
Cluster 30	Nrps	1	1821	-	-
M. matsumotoense DSM 44100^T					
Cluster 1	Bacteriocin	177374	188192	Lymphostin_biosynthetic_gene_cluster (33% of genes show similarity)	BGC0001006_c1
Cluster 2	Terpene	348025	369125	-	-
Cluster 3	T1pks	1	36298	-	-
Cluster 4	Nrps-Lantipeptide-T1pks	27142	95176	Bleomycin_biosynthetic_gene_cluster (15% of genes show similarity)	BGC0000963_c1
Cluster 5	Siderophore	471226	483010	Desferrioxamine_B_biosynthetic_gene_cluster (80% of genes show similarity)	BGC0000941_c1
Cluster 6	Terpene	674276	695463	-	-
Cluster 7	Lantipeptide	747288	766267	SapB_biosynthetic_gene_cluster (75% of genes show similarity)	BGC0000551_c1
Cluster 8	T1pks	37210	82969	Maduropeptin_biosynthetic_gene_cluster (28% of genes show similarity)	BGC0001008_c1
Cluster 9	T1pks	76123	121486	Neocarzinostatin_biosynthetic_gene_cluster (13% of genes show similarity)	BGC0000112_c1
Cluster 10	Nrps-Lassopeptide	173640	244675	Phoslactomycin_B_biosynthetic_gene_cluster (14% of genes show similarity)	BGC0000123_c1
Cluster 11	Nrps	282176	335688	-	-
Cluster 12	T1pks	323676	376549	A201A_biosynthetic_gene_cluster (8% of genes show similarity)	BGC0001138_c1
Cluster 13	Other	482630	508422	Auricin_deoxysugar_moieties_biosynthetic_gene_cluster (17% of genes show similarity)	BGC0000727_c1
Cluster 14	Phenazine-Indole	4627	46545	Fortimicin_biosynthetic_gene_cluster (11% of genes show similarity)	BGC0000695_c1
Cluster 15	Terpene	90017	110982	Phosphonoglycans_biosynthetic_gene_cluster (3% of genes show similarity)	BGC0000806_c1
Cluster 16	Nrps	219887	283921	Fluostatin_biosynthetic_gene_cluster (12% of genes show similarity)	BGC0000223_c1
Cluster 17	Lantipeptide	440598	458848	-	-
Cluster 18	Bacteriocin	41995	53938	-	-
Cluster 19	Other	1	28132	-	-
Cluster 20	Otherks-T1pks-Phosphoglyco	94279	174291	Gentamicin_biosynthetic_gene_cluster (10% of genes show similarity)	BGC0000696_c1
Cluster 21	Thiopeptide-Lantipeptide-Nrp	233312	307038	Leinamycin_biosynthetic_gene_cluster (5% of genes show similarity)	BGC0001101_c1
Cluster 22	T3pks	89647	130708	Alkyl-O-Dihydrogeranyl-Methoxyhydroquinones_biosynthetic_gene (71% of genes show similarity)	BGC0001077_c1
Cluster 23	T1pks	236338	259711	Monensin_biosynthetic_gene_cluster (8% of genes show similarity)	BGC0000100_c1
Cluster 24	Other	1	24351	Dactylocycline_biosynthetic_gene_cluster (42% of genes show similarity)	BGC0000216_c1
Cluster 25	T2pks	20446	62994	Dactylocycline_biosynthetic_gene_cluster (52% of genes show similarity)	BGC0000216_c1
Cluster 26	Nrps	165301	190042	Enduracidin_biosynthetic_gene_cluster (14% of genes show similarity)	BGC0000341_c1
Cluster 27	Nrps	75280	122620	-	-
Cluster 28	T2pks	58369	100923	Frankiamicin_biosynthetic_gene_cluster (28% of genes show similarity)	BGC0001197_c1
Cluster 29	T1pks-Nrps-Oligosaccharide-	1	60257	Lobosamide_biosynthetic_gene_cluster (43% of genes show similarity)	BGC0001303_c1
Cluster 30	Terpene	23369	44280	Sioxanthin_biosynthetic_gene_cluster (80% of genes show similarity)	BGC0001087_c4
Cluster 31	Nrps	1	30404	-	-
Cluster 32	Other	12912	55665	Chloramphenicol_biosynthetic_gene_cluster (94% of genes show similarity)	BGC0000893_c1
Cluster 33	Otherks	32247	73266	Galbonolides_biosynthetic_gene_cluster (33% of genes show similarity)	BGC0000065_c1
Cluster 34	Lantipeptide	36471	61050	-	-
Cluster 35	T1pks	1	58748	Salinilactam_biosynthetic_gene_cluster (88% of genes show similarity)	BGC0000142_c1
Cluster 36	Nrps	1	53106	Albachelin_biosynthetic_gene_cluster (90% of genes show similarity)	BGC0001211_c1
Cluster 37	Nrps	1	53471	Marformycins_biosynthetic_gene_cluster (12% of genes show similarity)	BGC0001214_c1
Cluster 38	T1pks	1	33367	Tetronasin_biosynthetic_gene_cluster (3% of genes show similarity)	BGC0000163_c1
Cluster 39	Nrps	1	24611	Stenothricin_biosynthetic_gene_cluster (9% of genes show similarity)	BGC0000431_c1
Cluster 40	Nrps	1	20183	-	-
Cluster 41	T1pks	1	15091	Lobosamide_biosynthetic_gene_cluster (13% of genes show similarity)	BGC0001303_c1
Cluster 42	Nrps	1	12500	Enduracidin_biosynthetic_gene_cluster (6% of genes show similarity)	BGC0000341_c1
Cluster 43	Nrps	1	10010	Friulimicin_biosynthetic_gene_cluster (9% of genes show similarity)	BGC0000354_c1
Cluster 44	Nrps	1	6769	-	-
Cluster 45	Nrps	1	5462	-	-
Cluster 46	Nrps	1	5252	-	-
Cluster 47	T1pks	1	4996	-	-
Cluster 48	Nrps	1	4922	-	-
M. mirobrigensis DSM 44830^T					
Cluster 1	Nrps	934704	995059	Heterobactin_biosynthetic_gene_cluster (18% of genes show similarity)	BGC0000371_c1
Cluster 2	Terpene	1252563	1273549	Phosphonoglycans_biosynthetic_gene_cluster (3% of genes show similarity)	BGC0000806_c1
Cluster 3	Terpene	1347915	1368829	Sioxanthin_biosynthetic_gene_cluster (80% of genes show similarity)	BGC0001087_c4
Cluster 4	Terpene	21171	42406	-	-
Cluster 5	Arylpolyene-T2pks	310514	357554	Xantholipin_biosynthetic_gene_cluster (14% of genes show similarity)	BGC0000279_c1
Cluster 6	Nrps-T1pks-Oligosaccharide-	570460	740591	Lobosamide_biosynthetic_gene_cluster (13% of genes show similarity)	BGC0001303_c1
Cluster 7	Nrps	427363	486776	Galbonolides_biosynthetic_gene_cluster (10% of genes show similarity)	BGC0000065_c1
Cluster 8	Lassopeptide	63627	86414	-	-
Cluster 9	Other	500347	537455	Streptomycin_biosynthetic_gene_cluster (10% of genes show similarity)	BGC0000717_c1
Cluster 10	T3pks	178741	219796	Alkyl-O-Dihydrogeranyl-Methoxyhydroquinones_biosynthetic_gene (57% of genes show similarity)	BGC0001077_c1
Cluster 11	T1pks-Otherks	72065	134113	Tetronasin_biosynthetic_gene_cluster (3% of genes show similarity)	BGC0000163_c1
Cluster 12	Terpene	157530	178480	-	-
Cluster 13	Bacteriocin-Terpene	37205	62841	Lymphostin_biosynthetic_gene_cluster (38% of genes show similarity)	BGC0001007_c1
M. narathiwatensis DSM 45248^T					
Cluster 1	Thiopeptide-Lantipeptide	929692	956099	Streptomycin_biosynthetic_gene_cluster (12% of genes show similarity)	BGC0000717_c1
Cluster 2	Lantipeptide	1053610	1078249	-	-
Cluster 3	Nrps-Arylpolyene	1249529	1333682	Kedarcidin_biosynthetic_gene_cluster (6% of genes show similarity)	BGC0000081_c1
Cluster 4	T2pks	1565839	1608348	Xantholipin_biosynthetic_gene_cluster (16% of genes show similarity)	BGC0000279_c1
Cluster 5	Nrps	1777323	1822622	-	-
Cluster 6	Terpene	1870388	1896431	Hopene_biosynthetic_gene_cluster (46% of genes show similarity)	BGC0000663_c1
Cluster 7	T1pks-Nrps	1916489	2051081	Neocarzilin_biosynthetic_gene_cluster (71% of genes show similarity)	BGC0000111_c1
Cluster 8	Siderophore	2094396	2106231	Desferrioxamine_B_biosynthetic_gene_cluster (80% of genes show similarity)	BGC0000941_c1
Cluster 9	Nrps-Lantipeptide-T1pks	2150746	2212579	Microsclerodermins_biosynthetic_gene_cluster (21% of genes show similarity)	BGC0001231_c1
Cluster 10	Terpene	2496067	2517314	Herbimycin_biosynthetic_gene_cluster (6% of genes show similarity)	BGC0000074_c1
Cluster 11	Nrps	2772865	2826061	-	-

Cluster 12	Nrps-T1pks	2815880	2893669	Nostopeptolide_biosynthetic_gene_cluster (37% of genes show similarity)	BGC0001028_c1
Cluster 13	T1pks-Nrps	3097985	3183111	Maduropeptin_biosynthetic_gene_cluster (58% of genes show similarity)	BGC0001008_c1
Cluster 14	Nrps-T1pks	3324594	3376569	-	-
Cluster 15	Terpene-Bacteriocin	3426015	3455971	Lymphostin_biosynthetic_gene_cluster (38% of genes show similarity)	BGC0001007_c1
Cluster 16	Terpene	3591227	3612177	-	-
Cluster 17	T3pks	4978608	5019657	Alkyl-O-Dihydrogeranyl-Methoxyhydroquinones_biosynthetic_gene (71% of genes show similarity)	BGC0001077_c1
Cluster 18	Terpene	5568192	5589145	Sioxanthin_biosynthetic_gene_cluster (80% of genes show similarity)	BGC0001087_c4
Cluster 19	Terpene	5650642	5671607	Phosphonoglycans_biosynthetic_gene_cluster (3% of genes show similarity)	BGC0000806_c1
M. nigra DSM 43818^{T}					
Cluster 1	Lantipeptide	74864	104490	-	-
Cluster 2	T1pks-Nrps	175443	402113	Macbecin_biosynthetic_gene_cluster (43% of genes show similarity)	BGC0000090_c1
Cluster 3	Lantipeptide-Nrps-T1pks	444907	731043	Friulimicin_biosynthetic_gene_cluster (15% of genes show similarity)	BGC0000354_c1
Cluster 4	Bacteriocin	943538	954422	Lymphostin_biosynthetic_gene_cluster (30% of genes show similarity)	BGC0001007_c1
Cluster 5	Terpene	1087783	1108730	-	-
Cluster 6	T3pks	2441005	2482054	Alkyl-O-Dihydrogeranyl-Methoxyhydroquinones_biosynthetic_gene (71% of genes show similarity)	BGC0001077_c1
Cluster 7	T1pks	2708251	2800911	Rifamycin_biosynthetic_gene_cluster (69% of genes show similarity)	BGC0000137_c1
Cluster 8	Terpene	3192455	3213387	Sioxanthin_biosynthetic_gene_cluster (100% of genes show similarity)	BGC0001087_c4
Cluster 9	Terpene	3267659	3288639	Phosphonoglycans_biosynthetic_gene_cluster (3% of genes show similarity)	BGC0000806_c1
Cluster 10	T1pks	3491739	3539607	Malleilactone_biosynthetic_gene_cluster (38% of genes show similarity)	BGC0001102_c1
Cluster 11	Lantipeptide	3728016	3752055	-	-
Cluster 12	Lassopeptide	4960003	4981875	-	-
Cluster 13	Other	5378747	5421305	Streptomycin_biosynthetic_gene_cluster (15% of genes show similarity)	BGC0000717_c1
Cluster 14	T1pks	5501217	5547000	Neocarzinostatin_biosynthetic_gene_cluster (50% of genes show similarity)	BGC0000112_c1
Cluster 15	T1pks	5538810	5584116	Neocarzinostatin_biosynthetic_gene_cluster (36% of genes show similarity)	BGC0000112_c1
Cluster 16	Siderophore	5612492	5624309	Desferrioxamine_B_biosynthetic_gene_cluster (83% of genes show similarity)	BGC0000940_c1
Cluster 17	Nrps	5630131	5683385	Tetronasin_biosynthetic_gene_cluster (3% of genes show similarity)	BGC0000163_c1
Cluster 18	Nrps	5680610	5738009	Friulimicin_biosynthetic_gene_cluster (15% of genes show similarity)	BGC0000354_c1
Cluster 19	T2pks	5787438	5830013	Xantholipin_biosynthetic_gene_cluster (14% of genes show similarity)	BGC0000279_c1
Cluster 20	Lassopeptide	5836399	5858171	-	-
Cluster 21	Siderophore	5926449	5939744	-	-
Cluster 22	Lantipeptide	6025242	6047839	SapB_biosynthetic_gene_cluster (100% of genes show similarity)	BGC0000551_c1
Cluster 23	Terpene	6176033	6197304	-	-
M. olivasterospora DSM 43868^{T}					
Cluster 1	Nrps-T1pks-Lantipeptide	615	64969	Calicheamicin_biosynthetic_gene_cluster (13% of genes show similarity)	BGC0000033_c1
Cluster 2	T1pks	78209	123965	Calicheamicin_biosynthetic_gene_cluster (16% of genes show similarity)	BGC0000033_c1
Cluster 3	Bacteriocin	563540	574442	Lymphostin_biosynthetic_gene_cluster (23% of genes show similarity)	BGC0001007_c1
Cluster 4	Terpene	686039	706980	-	-
Cluster 5	Terpene	1928194	1949420	2-methylisoborneol_biosynthetic_gene_cluster (75% of genes show similarity)	BGC0000657_c1
Cluster 6	T3pks	2020897	2061946	Alkyl-O-Dihydrogeranyl-Methoxyhydroquinones_biosynthetic_gene (71% of genes show similarity)	BGC0001077_c1
Cluster 7	Indole	2194822	2215952	Fortimicin_biosynthetic_gene_cluster (41% of genes show similarity)	BGC0000695_c1
Cluster 8	Terpene	2865488	2886444	Sioxanthin_biosynthetic_gene_cluster (60% of genes show similarity)	BGC0001087_c4
Cluster 9	Terpene	3009320	3030276	Phosphonoglycans_biosynthetic_gene_cluster (3% of genes show similarity)	BGC0000806_c1
Cluster 10	Nrps	3199722	3252356	Fluostatin_biosynthetic_gene_cluster (4% of genes show similarity)	BGC0000223_c1
Cluster 11	Nrps	3303054	3363427	-	-
Cluster 12	Linaridin	3853146	3873691	-	-
Cluster 13	Indole	4243162	4264319	-	-
Cluster 14	Terpene	4503533	4524627	Rifamycin_biosynthetic_gene_cluster (12% of genes show similarity)	BGC0000137_c1
Cluster 15	Phosphoglycolipid-Nucleosid	5121592	5152247	Moenomycin_biosynthetic_gene_cluster (22% of genes show similarity)	BGC0000805_c1
Cluster 16	Lantipeptide	5368878	5391481	Catenulipeptin_biosynthetic_gene_cluster (60% of genes show similarity)	BGC0000501_c1
Cluster 17	T2pks	5447450	5489988	Pradimicin_biosynthetic_gene_cluster (25% of genes show similarity)	BGC0000256_c1
Cluster 18	Terpene	5575474	5601508	Hopene_biosynthetic_gene_cluster (46% of genes show similarity)	BGC0000663_c1
Cluster 19	Terpene	5771429	5792640	-	-
Cluster 20	Other	5879353	5922373	-	-
Cluster 21	Indole	6241036	6262226	Pyoluteorin_biosynthetic_gene_cluster (15% of genes show similarity)	BGC0000128_c1
Cluster 22	Siderophore	6540652	6555092	Scabichelin_biosynthetic_gene_cluster (30% of genes show similarity)	BGC0000423_c1
M. pallida DSM 43817^{T}					
Cluster 1	T3pks	2957	44033	Herbimycin_biosynthetic_gene_cluster (20% of genes show similarity)	BGC0000074_c1
Cluster 2	Butyrolactone-T1pks	53258	90607	Pimaricin_biosynthetic_gene_cluster (29% of genes show similarity)	BGC0000125_c1
Cluster 3	Terpene	416323	437291	-	-
Cluster 4	Bacteriocin	595441	606394	Lymphostin_biosynthetic_gene_cluster (25% of genes show similarity)	BGC0001006_c1
Cluster 5	Thiopeptide-Lantipeptide	623729	653356	Lymphostin_biosynthetic_gene_cluster (16% of genes show similarity)	BGC0001006_c1
Cluster 6	Nrps-T1pks	660805	712522	-	-
Cluster 7	Lantipeptide	900723	925286	-	-
Cluster 8	Nrps	1047543	1105926	-	-
Cluster 9	Nrps-T1pks	1275911	1346248	Guadinomine_biosynthetic_gene_cluster (7% of genes show similarity)	BGC0000998_c1
Cluster 10	Fused	1366750	1392906	Leupyrrin_biosynthetic_gene_cluster (5% of genes show similarity)	BGC0000380_c1
Cluster 11	Terpene	1403880	1426045	Geosmin_biosynthetic_gene_cluster (100% of genes show similarity)	BGC0000661_c1
Cluster 12	Nrps-T1pks-T3pks	1672110	1782195	Crocacin_biosynthetic_gene_cluster (38% of genes show similarity)	BGC0000974_c1
Cluster 13	T3pks-Nrps	1768821	1821057	Feglymycin_biosynthetic_gene_cluster (47% of genes show similarity)	BGC0001233_c1
Cluster 14	Lantipeptide-Bacteriocin	1861084	1897978	Catenulipeptin_biosynthetic_gene_cluster (60% of genes show similarity)	BGC0000501_c1
Cluster 15	T2pks	2059021	2101548	Spore_pigment_biosynthetic_gene_cluster (41% of genes show similarity)	BGC0000271_c1
Cluster 16	Bacteriocin-Nrps-T1pks	2124317	2177395	Laspartomycin_biosynthetic_gene_cluster (6% of genes show similarity)	BGC0000379_c1
Cluster 17	Lassopeptide	2179457	2201986	-	-
Cluster 18	Nrps-T1pks	2251939	2330015	Calcium-dependent_antibiotic_biosynthetic_gene_cluster (10% of genes show similarity)	BGC0000315_c1
Cluster 19	Nrps-T1pks-Nucleoside-Lanti	2354805	2695554	Rifamycin_biosynthetic_gene_cluster (38% of genes show similarity)	BGC0000137_c1
Cluster 20	Nrps-T1pks	2791423	2884217	Daptomycin_biosynthetic_gene_cluster (4% of genes show similarity)	BGC0000336_c1
Cluster 21	Nrps-T1pks-Lantipeptide	2969640	3035108	Bleomycin_biosynthetic_gene_cluster (15% of genes show similarity)	BGC0000963_c1
Cluster 22	Other	3046487	3089123	A54145_biosynthetic_gene_cluster (5% of genes show similarity)	BGC0000291_c1
Cluster 23	T2pks-Otherks-T1pks	3750558	3809934	TLN-05220_biosynthetic_gene_cluster (86% of genes show similarity)	BGC0001062_c1
Cluster 24	T3pks	3954289	3999079	Furaquinocin_A_biosynthetic_gene_cluster (34% of genes show similarity)	BGC0001078_c1
Cluster 25	T1pks-Otherks	5204106	5256178	Kanamycin_biosynthetic_gene_cluster (23% of genes show similarity)	BGC0000706_c1
Cluster 26	Siderophore	5412966	5424780	Desferrioxamine_B_biosynthetic_gene_cluster (80% of genes show similarity)	BGC0000941_c1
Cluster 27	Terpene	5637113	5658069	-	-

Cluster 28	Terpene	5723481	5744419	Carotenoid_biosynthetic_gene_cluster (18% of genes show similarity)	BGC0000633_c1
Cluster 29	Amglyccycl	6288733	6309917	Gentamicin_biosynthetic_gene_cluster (27% of genes show similarity)	BGC0000696_c1
Cluster 30	T3pks	6466046	6507095	Alkyl-O-Dihydrogeranyl-Methoxyhydroquinones_biosynthetic_gene (57% of genes show similarity)	BGC0001077_c1
Cluster 31	Other	7379495	7445224	Diazepinomicin_biosynthetic_gene_cluster (67% of genes show similarity)	BGC0000679_c1

M. peucetia DSM 43363^T					
Cluster 1	Nrps	20096	66043	Nocathiacin_biosynthetic_gene_cluster (4% of genes show similarity)	BGC0000609_c1
Cluster 2	Nrps-T1pks	609468	715770	Lipomycin_biosynthetic_gene_cluster (22% of genes show similarity)	BGC0001003_c1
Cluster 3	Nrps-Lantipeptide	701091	785981	Livipeptin_biosynthetic_gene_cluster (100% of genes show similarity)	BGC0001168_c1
Cluster 4	Other	1288069	1332211	Kedarcidin_biosynthetic_gene_cluster (1% of genes show similarity)	BGC0000081_c1
Cluster 5	Nrps-Lantipeptide-T1pks	1382936	1493452	Azicemicin_biosynthetic_gene_cluster (11% of genes show similarity)	BGC0000202_c1
Cluster 6	Terpene	1735158	1756165	Phosphonoglycans_biosynthetic_gene_cluster (3% of genes show similarity)	BGC0000806_c1
Cluster 7	Terpene	1826027	1846965	Sioxanthin_biosynthetic_gene_cluster (80% of genes show similarity)	BGC0001087_c4
Cluster 8	Nrps	2248759	2296635	Gentamicin_biosynthetic_gene_cluster (6% of genes show similarity)	BGC0000696_c1
Cluster 9	T3pks-Thiopeptide-Lantipept	2489822	2544045	Alkyl-O-Dihydrogeranyl-Methoxyhydroquinones_biosynthetic_gene (71% of genes show similarity)	BGC0001077_c1
Cluster 10	Terpene	3994398	4015279	-	-
Cluster 11	Bacteriocin-Terpene	4145312	4169014	Lymphostin_biosynthetic_gene_cluster (38% of genes show similarity)	BGC0001007_c1
Cluster 12	Nrps-T1pks	4197876	4247682	Streptomycin_biosynthetic_gene_cluster (2% of genes show similarity)	BGC0000717_c1
Cluster 13	Nrps-Lantipeptide	4528059	4629302	Maduropeptin_biosynthetic_gene_cluster (22% of genes show similarity)	BGC0001008_c1
Cluster 14	Nrps-Lantipeptide	4700901	4785135	Jagaricin_biosynthetic_gene_cluster (13% of genes show similarity)	BGC0001127_c1
Cluster 15	Lantipeptide-Nrps	4779309	4851180	Calicheamicin_biosynthetic_gene_cluster (10% of genes show similarity)	BGC0000033_c1
Cluster 16	T1pks	4866021	4911843	Maduropeptin_biosynthetic_gene_cluster (24% of genes show similarity)	BGC0001008_c1
Cluster 17	T2pks	5055456	5097956	Xantholipin_biosynthetic_gene_cluster (16% of genes show similarity)	BGC0000279_c1
Cluster 18	Nrps-T1pks	5210715	5265000	Oxazolomycin_biosynthetic_gene_cluster (6% of genes show similarity)	BGC0001106_c1
Cluster 19	Terpene	5400864	5422123	-	-
Cluster 20	Lantipeptide	5606287	5628884	SapB_biosynthetic_gene_cluster (75% of genes show similarity)	BGC0000551_c1
Cluster 21	T1pks-Nrps-Siderophore	6069256	6314504	Rifamycin_biosynthetic_gene_cluster (21% of genes show similarity)	BGC0000136_c1
Cluster 22	T1pks	6378085	6437645	Tetronasin_biosynthetic_gene_cluster (9% of genes show similarity)	BGC0000163_c1
Cluster 23	Nrps	6433763	6480970	Sanglifehrin_A_biosynthetic_gene_cluster (4% of genes show similarity)	BGC0001042_c1
Cluster 24	Other	6553120	6593515	Thiocoraline_biosynthetic_gene_cluster (5% of genes show similarity)	BGC0000445_c1
Cluster 25	Lantipeptide	7201037	7226890	Chrysomycin_biosynthetic_gene_cluster (5% of genes show similarity)	BGC0000211_c1

M. purpureochromogenes DSM 43821^T					
Cluster 1	Terpene	163871	184827	Phosphonoglycans_biosynthetic_gene_cluster (3% of genes show similarity)	BGC0000806_c1
Cluster 2	Terpene	351166	372083	Sioxanthin_biosynthetic_gene_cluster (80% of genes show similarity)	BGC0001087_c4
Cluster 3	T3pks	1017815	1058882	Alkyl-O-Dihydrogeranyl-Methoxyhydroquinones_biosynthetic_gene (71% of genes show similarity)	BGC0001077_c1
Cluster 4	Terpene	2522559	2543500	-	-
Cluster 5	Bacteriocin	2683059	2693877	Lymphostin_biosynthetic_gene_cluster (33% of genes show similarity)	BGC0001006_c1
Cluster 6	T2pks	3191116	3233649	Xantholipin_biosynthetic_gene_cluster (14% of genes show similarity)	BGC0000279_c1
Cluster 7	T2pks	3313301	3355801	Alnumycin_biosynthetic_gene_cluster (25% of genes show similarity)	BGC0000195_c1
Cluster 8	Lantipeptide	3529135	3551792	SapB_biosynthetic_gene_cluster (100% of genes show similarity)	BGC0000551_c1
Cluster 9	Terpene	3777690	3798916	-	-
Cluster 10	Nrps-T1pks-Lantipeptide	4243750	4311516	Bleomycin_biosynthetic_gene_cluster (15% of genes show similarity)	BGC0000963_c1
Cluster 11	Lantipeptide	4411399	4436039	Actinomycin_biosynthetic_gene_cluster (10% of genes show similarity)	BGC0000296_c1
Cluster 12	Other	4539179	4581764	Streptomycin_biosynthetic_gene_cluster (14% of genes show similarity)	BGC0000717_c1
Cluster 13	Siderophore	4816917	4828701	Desferrioxamine_B_biosynthetic_gene_cluster (80% of genes show similarity)	BGC0000941_c1
Cluster 14	T1pks	4957593	5066681	Cremimycin_biosynthetic_gene_cluster (37% of genes show similarity)	BGC0000042_c1
Cluster 15	Nrps-T1pks	5072989	5135703	Leinamycin_biosynthetic_gene_cluster (2% of genes show similarity)	BGC0001101_c1

M. rhizosphaerae DSM 45431^T					
Cluster 1	T3pks	449438	490487	Alkyl-O-Dihydrogeranyl-Methoxyhydroquinones_biosynthetic_gene (71% of genes show similarity)	BGC0001077_c1
Cluster 2	Oligosaccharide	704839	725546	Rifamycin_biosynthetic_gene_cluster (5% of genes show similarity)	BGC0000137_c1
Cluster 3	Terpene	1127095	1148021	Sioxanthin_biosynthetic_gene_cluster (60% of genes show similarity)	BGC0001087_c4
Cluster 4	Terpene	1196055	1217059	Phosphonoglycans_biosynthetic_gene_cluster (3% of genes show similarity)	BGC0000806_c1
Cluster 5	Other	4014099	4056624	-	-
Cluster 6	Terpene	4734614	4755846	-	-
Cluster 7	Terpene-Bacteriocin	5999173	6027921	Lymphostin_biosynthetic_gene_cluster (38% of genes show similarity)	BGC0001007_c1

M. rifamycinica DSM 44983^T					
Cluster 1	Nrps	1	23290	Streptolydigin_biosynthetic_gene_cluster (10% of genes show similarity)	BGC0001046_c1
Cluster 2	Nrps	8682	59955	Griseobactin_biosynthetic_gene_cluster (11% of genes show similarity)	BGC0000368_c1
Cluster 3	T1pks	72434	165193	Rifamycin_biosynthetic_gene_cluster (34% of genes show similarity)	BGC0000136_c1
Cluster 4	Terpene	766353	787540	-	-
Cluster 5	Siderophore	992899	1004683	Desferrioxamine_B_biosynthetic_gene_cluster (80% of genes show similarity)	BGC0000941_c1
Cluster 6	Lantipeptide	1016065	1038719	SapB_biosynthetic_gene_cluster (100% of genes show similarity)	BGC0000551_c1
Cluster 7	T2pks	1172477	1215058	Xantholipin_biosynthetic_gene_cluster (10% of genes show similarity)	BGC0000279_c1
Cluster 8	T1pks	1454503	1507379	A201A_biosynthetic_gene_cluster (8% of genes show similarity)	BGC0001138_c1
Cluster 9	T2pks-Butyrolactone	1660933	1724146	Arimetamycin_biosynthetic_gene_cluster (13% of genes show similarity)	BGC0000199_c1
Cluster 10	T1pks-Otherks	1739106	1849067	Tiacumicin_B_biosynthetic_gene_cluster (80% of genes show similarity)	BGC0000165_c1
Cluster 11	Nrps-T1pks-Blactam-T3pks	1890125	2056823	Kedarcidin_biosynthetic_gene_cluster (16% of genes show similarity)	BGC0000081_c1
Cluster 12	T1pks-Nrps-Oligosaccharide-	2231871	2375211	Lobosamide_biosynthetic_gene_cluster (82% of genes show similarity)	BGC0001303_c1
Cluster 13	Bacteriocin	3212586	3245828	-	-
Cluster 14	Nrps-T3pks	3674033	3726253	Feglymycin_biosynthetic_gene_cluster (36% of genes show similarity)	BGC0001233_c1
Cluster 15	T1pks-Otherks	3716925	3782793	Neocarzilin_biosynthetic_gene_cluster (28% of genes show similarity)	BGC0000111_c1
Cluster 16	Nrps	3848439	3911787	Fluostatin_biosynthetic_gene_cluster (12% of genes show similarity)	BGC0000223_c1
Cluster 17	Terpene	4049129	4070094	-	-
Cluster 18	Indole	4107055	4128155	Fortimicin_biosynthetic_gene_cluster (11% of genes show similarity)	BGC0000695_c1
Cluster 19	Terpene	4170455	4191366	Sioxanthin_biosynthetic_gene_cluster (80% of genes show similarity)	BGC0001087_c4
Cluster 20	Nrps-T1pks	4504965	4584315	Guadinomine_biosynthetic_gene_cluster (61% of genes show similarity)	BGC0000998_c1
Cluster 21	T3pks-Bacteriocin	4591178	4655155	Furaquinocin_A_biosynthetic_gene_cluster (34% of genes show similarity)	BGC0001078_c1
Cluster 22	Indole	4675935	4697200	Echinomycin_biosynthetic_gene_cluster (11% of genes show similarity)	BGC0000339_c1
Cluster 23	T3pks	4927017	4968078	Alkyl-O-Dihydrogeranyl-Methoxyhydroquinones_biosynthetic_gene (71% of genes show similarity)	BGC0001077_c1
Cluster 24	Bacteriocin	6522607	6533425	Lymphostin_biosynthetic_gene_cluster (30% of genes show similarity)	BGC0001007_c1
Cluster 25	Other	6987350	7011269	Friulimicin_biosynthetic_gene_cluster (9% of genes show similarity)	BGC0000354_c1

M. saelicesensis DSM 44871^{T}					
Cluster 1	T2pks-Siderophore	754563	820653	Xantholipin_biosynthetic_gene_cluster (22% of genes show similarity)	BGC0000279_c1
Cluster 2	Terpene	1100468	1121454	-	-
Cluster 3	Terpene	1215510	1236433	Sioxanthin_biosynthetic_gene_cluster (80% of genes show similarity)	BGC0001087_c4
Cluster 4	Siderophore	453742	465589	Desferrioxamine_B_biosynthetic_gene_cluster (83% of genes show similarity)	BGC0000940_c1
Cluster 5	Lantipeptide	951170	978616	SRO15-3108_biosynthetic_gene_cluster (50% of genes show similarity)	BGC0000554_c1
Cluster 6	T3pks	453579	494628	Alkyl-O-Dihydrogeranyl-Methoxyhydroquinones_biosynthetic_gene (71% of genes show similarity)	BGC0001077_c1
Cluster 7	Bacteriocin	37272	48117	Lymphostin_biosynthetic_gene_cluster (33% of genes show similarity)	BGC0001006_c1
Cluster 8	Lantipeptide	572008	594605	SapB_biosynthetic_gene_cluster (100% of genes show similarity)	BGC0000551_c1
Cluster 9	Terpene	23423	44724	-	-
Cluster 10	Terpene	185480	206427	-	-
Cluster 11	T2pks	250660	273993	Xantholipin_biosynthetic_gene_cluster (12% of genes show similarity)	BGC0000279_c1

M. sagamiensis DSM 43912^{T}					
Cluster 1	Amglyccycl	40595	61779	Gentamicin_biosynthetic_gene_cluster (27% of genes show similarity)	BGC0000696_c1
Cluster 2	T3pks	235814	276863	Alkyl-O-Dihydrogeranyl-Methoxyhydroquinones_biosynthetic_gene (57% of genes show similarity)	BGC0001077_c1
Cluster 3	Other	1207770	1272397	Diazepinomicin_biosynthetic_gene_cluster (67% of genes show similarity)	BGC0000679_c1
Cluster 4	T3pks	1451624	1492700	Herbimycin_biosynthetic_gene_cluster (10% of genes show similarity)	BGC0000074_c1
Cluster 5	Terpene	1841890	1862840	-	-
Cluster 6	Bacteriocin	2021016	2031879	Lymphostin_biosynthetic_gene_cluster (23% of genes show similarity)	BGC0001007_c1
Cluster 7	Nrps-T1pks	2092060	2142915	-	-
Cluster 8	Other	2248039	2290642	Taromycin_biosynthetic_gene_cluster (6% of genes show similarity)	BGC0000439_c1
Cluster 9	Otherks-T1pks	2401456	2453697	-	-
Cluster 10	Thiopeptide-Lantipeptide	2603523	2633379	Muraymycin_biosynthetic_gene_cluster (10% of genes show similarity)	BGC0001020_c1
Cluster 11	T1pks	2659580	2709470	Lobosamide_biosynthetic_gene_cluster (13% of genes show similarity)	BGC0001303_c1
Cluster 12	Nrps-T1pks-T3pks	2878007	2985716	Crocacin_biosynthetic_gene_cluster (38% of genes show similarity)	BGC0000974_c1
Cluster 13	T3pks-Nrps	2990681	3042915	Feglymycin_biosynthetic_gene_cluster (47% of genes show similarity)	BGC0001233_c1
Cluster 14	Lantipeptide-Bacteriocin	3072679	3106783	SapB_biosynthetic_gene_cluster (75% of genes show similarity)	BGC0000551_c1
Cluster 15	T2pks	3295345	3337872	Xantholipin_biosynthetic_gene_cluster (10% of genes show similarity)	BGC0000279_c1
Cluster 16	Nrps-T1pks	3363721	3450479	Fosfomycin_biosynthetic_gene_cluster (10% of genes show similarity)	BGC0000938_c1
Cluster 17	Otherks-Nrps	3455232	3510125	Kedarcidin_biosynthetic_gene_cluster (3% of genes show similarity)	BGC0000081_c1
Cluster 18	Nrps-T1pks-Lantipeptide-Nuc	3643482	3710447	Muraymycin_biosynthetic_gene_cluster (18% of genes show similarity)	BGC0001020_c1
Cluster 19	Lantipeptide-Nrps	3701898	3755856	Maklamicin_biosynthetic_gene_cluster (6% of genes show similarity)	BGC0001288_c1
Cluster 20	T1pks	3745620	3960633	Rifamycin_biosynthetic_gene_cluster (38% of genes show similarity)	BGC0000137_c1
Cluster 21	Nrps-T1pks-Lantipeptide	4121786	4187206	Bleomycin_biosynthetic_gene_cluster (15% of genes show similarity)	BGC0000963_c1
Cluster 22	T2pks-Otherks-T1pks	4457838	4517425	TLN-05220_biosynthetic_gene_cluster (96% of genes show similarity)	BGC0001062_c1
Cluster 23	Nrps	4573781	4616549	Quartromicin_biosynthetic_gene_cluster (5% of genes show similarity)	BGC0000133_c1
Cluster 24	Nrps	4679909	4737752	-	-
Cluster 25	Other	5236461	5277186	-	-
Cluster 26	Butyrolactone-T1pks	5880613	5917928	Concanamycin_A_biosynthetic_gene_cluster (21% of genes show similarity)	BGC0000040_c1
Cluster 27	T1pks	5925381	5977856	Kanamycin_biosynthetic_gene_cluster (23% of genes show similarity)	BGC0000706_c1
Cluster 28	Siderophore	6039970	6051826	Desferrioxamine_B_biosynthetic_gene_cluster (80% of genes show similarity)	BGC0000941_c1
Cluster 29	Terpene	6258030	6278986	-	-
Cluster 30	Terpene	6353709	6374632	Sioxanthin_biosynthetic_gene_cluster (100% of genes show similarity)	BGC0001087_c4
Cluster 31	Lantipeptide	6739276	6763292	-	-

M. sediminicola DSM 45794^{T}					
Cluster 1	Terpene	37419	58384	Phosphonoglycans_biosynthetic_gene_cluster (3% of genes show similarity)	BGC0000806_c1
Cluster 2	Terpene	138174	159100	Sioxanthin_biosynthetic_gene_cluster (100% of genes show similarity)	BGC0001087_c4
Cluster 3	T3pks	764960	806018	Alkyl-O-Dihydrogeranyl-Methoxyhydroquinones_biosynthetic_gene (71% of genes show similarity)	BGC0001077_c1
Cluster 4	Terpene	2331143	2352093	-	-
Cluster 5	Bacteriocin-Terpene	2494305	2518774	Lymphostin_biosynthetic_gene_cluster (38% of genes show similarity)	BGC0001007_c1
Cluster 6	Nrps	2717818	2820800	Laspartomycin_biosynthetic_gene_cluster (13% of genes show similarity)	BGC0000379_c1
Cluster 7	Siderophore	2819851	2833062	-	-
Cluster 8	Nrps	2960718	3030084	Scabichelin_biosynthetic_gene_cluster (20% of genes show similarity)	BGC0000423_c1
Cluster 9	Siderophore	3202480	3214264	Desferrioxamine_B_biosynthetic_gene_cluster (80% of genes show similarity)	BGC0000941_c1
Cluster 10	T2pks	3231162	3273662	Actinorhodin_biosynthetic_gene_cluster (63% of genes show similarity)	BGC0000194_c1
Cluster 11	T2pks-Ladderane	3463600	3510500	Xantholipin_biosynthetic_gene_cluster (14% of genes show similarity)	BGC0000279_c1
Cluster 12	Terpene	3717000	3738187	Nocathiacin_biosynthetic_gene_cluster (4% of genes show similarity)	BGC0000609_c1
Cluster 13	T1pks	4006422	4228153	Rifamycin_biosynthetic_gene_cluster (35% of genes show similarity)	BGC0000137_c1
Cluster 14	Nrps-T1pks	4544535	4592857	-	-
Cluster 15	Nrps-T1pks	460009	522564	Tallysomycin_biosynthetic_gene_cluster (5% of genes show similarity)	BGC0001048_c1

M. siamensis DSM 45097^{T}					
Cluster 1	T3pks	210670	251725	Alkyl-O-Dihydrogeranyl-Methoxyhydroquinones_biosynthetic_gene (57% of genes show similarity)	BGC0001077_c1
Cluster 2	Terpene	863112	884026	Sioxanthin_biosynthetic_gene_cluster (100% of genes show similarity)	BGC0001087_c4
Cluster 3	Terpene	962664	983650	Phosphonoglycans_biosynthetic_gene_cluster (3% of genes show similarity)	BGC0000806_c1
Cluster 4	Nrps	1246388	1306737	Lomaiviticin_biosynthetic_gene_cluster (3% of genes show similarity)	BGC0000240_c1
Cluster 5	Nrps	1674796	1752540	WS9326_biosynthetic_gene_cluster (10% of genes show similarity)	BGC0001297_c1
Cluster 6	Otherks-T1pks	2553807	2615642	Tetronasin_biosynthetic_gene_cluster (3% of genes show similarity)	BGC0000163_c1
Cluster 7	Other	3121150	3163675	Streptomycin_biosynthetic_gene_cluster (12% of genes show similarity)	BGC0000717_c1
Cluster 8	Nrps	3311723	3371133	Herboxidiene_biosynthetic_gene_cluster (2% of genes show similarity)	BGC0001065_c1
Cluster 9	Terpene	3805182	3826417	-	-
Cluster 10	T2pks	4145637	4188149	Xantholipin_biosynthetic_gene_cluster (14% of genes show similarity)	BGC0000279_c1
Cluster 11	Nrps-T1pks	4448303	4510815	Bleomycin_biosynthetic_gene_cluster (6% of genes show similarity)	BGC0000963_c1
Cluster 12	Nrps	4500492	4556130	Kedarcidin_biosynthetic_gene_cluster (6% of genes show similarity)	BGC0000081_c1
Cluster 13	T1pks-Nrps	4542357	4627431	Kedarcidin_biosynthetic_gene_cluster (14% of genes show similarity)	BGC0000081_c1
Cluster 14	Terpene-Bacteriocin	4769548	4797833	Lymphostin_biosynthetic_gene_cluster (38% of genes show similarity)	BGC0001007_c1
Cluster 15	Terpene	4935385	4956335	-	-

M. tulbaghiae DSM 45142^{T}					
Cluster 1	Lantipeptide	63682	96492	Pentalenolactone_biosynthetic_gene_cluster (15% of genes show similarity)	BGC0000678_c1
Cluster 2	Nrps-T1pks-Lantipeptide	71511	139133	Bleomycin_biosynthetic_gene_cluster (12% of genes show similarity)	BGC0000963_c1

Cluster 3	Nrps	139934	173896	Azinomycin_B_biosynthetic_gene_cluster (19% of genes show similarity)	BGC0000960_c1
Cluster 4	Terpene	417579	438505	Sioxanthin_biosynthetic_gene_cluster (100% of genes show similarity)	BGC0001087_c4
Cluster 5	Terpene	515530	536495	Phosphonoglycans_biosynthetic_gene_cluster (3% of genes show similarity)	BGC0000806_c1
Cluster 6	Nrps-Blactam	1	51718	Pristinamycin_biosynthetic_gene_cluster (5% of genes show similarity)	BGC0000952_c1
Cluster 7	Nrps-Transatpks	43911	130990	Leinamycin_biosynthetic_gene_cluster (14% of genes show similarity)	BGC0001101_c1
Cluster 8	Arylpolyene-Nrps	230909	304772	Kedarcidin_biosynthetic_gene_cluster (6% of genes show similarity)	BGC0000081_c1
Cluster 9	Oligosaccharide-Nrps-Terpen	365544	414637	Lobosamide_biosynthetic_gene_cluster (10% of genes show similarity)	BGC0001303_c1
Cluster 10	T2pks	597042	639616	Xantholipin_biosynthetic_gene_cluster (16% of genes show similarity)	BGC0000279_c1
Cluster 11	Terpene	838131	860115	Nocathiacin_biosynthetic_gene_cluster (4% of genes show similarity)	BGC0000609_c1
Cluster 12	Terpene-Bacteriocin	17740	47320	Lymphostin_biosynthetic_gene_cluster (33% of genes show similarity)	BGC0001006_c1
Cluster 13	Terpene	194258	215208	-	-
Cluster 14	Siderophore	249511	261295	Desferrioxamine_B_biosynthetic_gene_cluster (80% of genes show similarity)	BGC0000941_c1
Cluster 15	Nrps	257619	311006	Azicemicin_biosynthetic_gene_cluster (13% of genes show similarity)	BGC0000202_c1
Cluster 16	T3pks	146953	188005	Alkyl-O-Dihydrogeranyl-Methoxyhydroquinones_biosynthetic_gene (71% of genes show similarity)	BGC0001077_c1
M. viridifaciens DSM 43909^{T}					
Cluster 1	Terpene	95305	116306	Phosphonoglycans_biosynthetic_gene_cluster (3% of genes show similarity)	BGC0000806_c1
Cluster 2	Terpene	176736	197668	Sioxanthin_biosynthetic_gene_cluster (80% of genes show similarity)	BGC0001087_c4
Cluster 3	T3pks	731601	772650	Alkyl-O-Dihydrogeranyl-Methoxyhydroquinones_biosynthetic_gene (71% of genes show similarity)	BGC0001077_c1
Cluster 4	Terpene	2129233	2150183	-	-
Cluster 5	Bacteriocin	2270135	2280995	Lymphostin_biosynthetic_gene_cluster (30% of genes show similarity)	BGC0001007_c1
Cluster 6	Nrps-T1pks	2556787	2617345	-	-
Cluster 7	Nrps	2853458	2905558	-	-
Cluster 8	Nrps-T1pks	2887509	2966247	Collismycin_A_biosynthetic_gene_cluster (14% of genes show similarity)	BGC0000973_c1
Cluster 9	T1pks	3032573	3082382	Tetrocarcin_A_biosynthetic_gene_cluster (11% of genes show similarity)	BGC0000162_c1
Cluster 10	Nrps	3084673	3149130	Daptomycin_biosynthetic_gene_cluster (6% of genes show similarity)	BGC0000336_c1
Cluster 11	Arylpolyene-Nrps	3228843	3302851	Kedarcidin_biosynthetic_gene_cluster (7% of genes show similarity)	BGC0000081_c1
Cluster 12	Nrps-Lantipeptide-T1pks	3320036	3387931	Bleomycin_biosynthetic_gene_cluster (15% of genes show similarity)	BGC0000963_c1
Cluster 13	Terpene	3612002	3638048	Hopene_biosynthetic_gene_cluster (46% of genes show similarity)	BGC0000663_c1
Cluster 14	Terpene	4010082	4031335	Meilingmycin_biosynthetic_gene_cluster (2% of genes show similarity)	BGC0000093_c1
Cluster 15	Lantipeptide	4720747	4770779	A-500359s_biosynthetic_gene_cluster (5% of genes show similarity)	BGC0000949_c1
Cluster 16	T2pks	4928976	4971482	Xantholipin_biosynthetic_gene_cluster (14% of genes show similarity)	BGC0000279_c1
Cluster 17	Thiopeptide-Lantipeptide	6434095	6465815	Nosiheptide_biosynthetic_gene_cluster (57% of genes show similarity)	BGC0000610_c1
M. yangpuensis DSM 45577^{T}					
Cluster 1	Terpene	368031	388993	Sioxanthin_biosynthetic_gene_cluster (80% of genes show similarity)	BGC0001087_c4
Cluster 2	Terpene	444108	465061	Phosphonoglycans_biosynthetic_gene_cluster (3% of genes show similarity)	BGC0000806_c1
Cluster 3	Siderophore	697880	709712	Desferrioxamine_B_biosynthetic_gene_cluster (66% of genes show similarity)	BGC0000940_c1
Cluster 4	Lantipeptide	725903	748956	Azicemicin_biosynthetic_gene_cluster (6% of genes show similarity)	BGC0000202_c1
Cluster 5	Nrps-T1pks	748793	804017	Collismycin_A_biosynthetic_gene_cluster (7% of genes show similarity)	BGC0000973_c1
Cluster 6	Blactam	1690588	1712423	Tabtoxin_biosynthetic_gene_cluster (13% of genes show similarity)	BGC0000846_c1
Cluster 7	T2pks-Terpene	2331227	2393265	Fluostatin_biosynthetic_gene_cluster (38% of genes show similarity)	BGC0000223_c1
Cluster 8	T1pks	2879854	2930792	Tetronasin_biosynthetic_gene_cluster (3% of genes show similarity)	BGC0000163_c1
Cluster 9	Nrps	2989272	3032754	-	-
Cluster 10	T1pks	3181257	3233908	Gentamicin_biosynthetic_gene_cluster (7% of genes show similarity)	BGC0000696_c1
Cluster 11	Nrps	3274981	3341198	Calicheamicin_biosynthetic_gene_cluster (8% of genes show similarity)	BGC0000033_c1
Cluster 12	Nrps	3408831	3461088	Kosinostatin_biosynthetic_gene_cluster (6% of genes show similarity)	BGC0001073_c1
Cluster 13	T2pks	3569212	3611727	Pradimicin_biosynthetic_gene_cluster (25% of genes show similarity)	BGC0000256_c1
Cluster 14	Lantipeptide-Terpene	3788276	3828149	Catenulipeptin_biosynthetic_gene_cluster (40% of genes show similarity)	BGC0000501_c1
Cluster 15	T1pks	3868287	3913989	Dynemicin_biosynthetic_gene_cluster (35% of genes show similarity)	BGC0001060_c1
Cluster 16	T1pks	4035896	4083197	Mirubactin_biosynthetic_gene_cluster (21% of genes show similarity)	BGC0000392_c1
Cluster 17	Bacteriocin	4356679	4367704	Lymphostin_biosynthetic_gene_cluster (30% of genes show similarity)	BGC0001007_c1
Cluster 18	Resorcinol	5620434	5662419	Pyrrolomycin_biosynthetic_gene_cluster (5% of genes show similarity)	BGC0000130_c1
Cluster 19	T3pks	6059724	6100776	Alkyl-O-Dihydrogeranyl-Methoxyhydroquinones_biosynthetic_gene (71% of genes show similarity)	BGC0001077_c1
Cluster 20	Linaridin	6246330	6266899	Legonaridin_biosynthetic_gene_cluster (22% of genes show similarity)	BGC0001188_c1
Cluster 21	Nrps	6299551	6385523	Azinomycin_B_biosynthetic_gene_cluster (12% of genes show similarity)	BGC0000960_c1
Cluster 22	Phenazine-Indole	6392480	6426340	Phenazine_biosynthetic_gene_cluster (44% of genes show similarity)	BGC0001080_c1
M. zamorensis DSM 45600^{T}					
Cluster 1	Nrps-T1pks	1974303	2022781	-	-
Cluster 2	T1pks	2092352	2275881	Nystatin-like_Pseudonocardia_polyene_biosynthetic_gene_cluster (34% of genes show similarity)	BGC0000116_c1
Cluster 3	Terpene	2500919	2522238	-	-
Cluster 4	T2pks	3029628	3072191	Xantholipin_biosynthetic_gene_cluster (14% of genes show similarity)	BGC0000279_c1
Cluster 5	Siderophore	3245755	3257602	Desferrioxamine_B_biosynthetic_gene_cluster (66% of genes show similarity)	BGC0000940_c1
Cluster 6	Nrps-T1pks	3779582	3842169	Bleomycin_biosynthetic_gene_cluster (9% of genes show similarity)	BGC0000963_c1
Cluster 7	Bacteriocin	4229654	4240472	Lymphostin_biosynthetic_gene_cluster (33% of genes show similarity)	BGC0001006_c1
Cluster 8	Terpene	4389453	4410403	-	-
Cluster 9	T3pks	5856920	5897969	Alkyl-O-Dihydrogeranyl-Methoxyhydroquinones_biosynthetic_gene (71% of genes show similarity)	BGC0001077_c1
Cluster 10	Terpene	6473972	6494895	Sioxanthin_biosynthetic_gene_cluster (100% of genes show similarity)	BGC0001087_c4
Cluster 11	Terpene	6605153	6626115	Phosphonoglycans_biosynthetic_gene_cluster (3% of genes show similarity)	BGC0000806_c1
Cluster 12	Siderophore	6927541	6940764	-	-

Supplementary Table 8. Origin of strains analysed in this study.

	DSMZ number	History of the strain
M. aurantiaca ATCC 27029^{T}	DSM 43813	<- NRRL <- ATCC <- G.F. Gauze, INA
M. aurantiaca L5	-	< M. Valdes; L5
M. aurantiaca DSM 45487	DSM 45487	<- J. Hamedi, Univ. Tehran, Iran <- F. Mohammadipanah; UTMC 555
M. auratinigra DSM 44815^{T}	DSM 44815	<- Ch. Thawai; TT1-11 (*Micromonospora aurantionigra*)
M. carbonacea DSM 43168^{T}	DSM 43168	<- KCC <- NRRL <- Schering AG <- A.Woyciesjes
M. chaiyaphumensis DSM 45246^{T}	DSM 45246	<- JCM/RIKEN; JCM 12873 <- S. Tanasupawat; MC5-1
M. chalcea DSM 43026^{T}	DSM 43026	<- KCC <- CBS <- ATCC <- Chas. Pfizer, 1464-217L
M. chersina DSM 44151^{T}	DSM 44151	<- ATCC <- Bristol-Meyers Co., M 956-1
M. chokoriensis DSM 45160^{T}	DSM 45160	<- JCM/RIKEN <- I. Ara; 2-19(6)
M. citrea DSM 43903^{T}	DSM 43903	<- JCM (*Micromonospora citrea*) <- KCC <- X. Yan, 71-97
M. coriariae DSM 44875^{T}	DSM 44875	<- M. E. Trujillo; NAR01
M. coxensis DSM 45161^{T}	DSM 45161	<- JCM/RIKEN <- I. Ara; 2-30-b(28)
M. cremea DSM 45599^{T}	DSM 45599	<- M. E. Trujillo, Universidad de Salamanca, Spain; CR 30 <- L. Carro
M. eburnea DSM 44814^{T}	DSM 44814	<- Ch. Thawai; LK2-10
M. echinaurantiaca DSM 43904^{T}	DSM 43904	<- JCM (*Micromonospora echinoaurantiaca*) <- KCC <- X. Yan, 65-m50
M. echinofusca DSM 43913^{T}	DSM 43913	<- JCM (*Micromonospora echinobrunnea*) <- KCC <- X. Yan, 71-m68
M. echinospora DSM 1040	DSM 43816	<- NRRL <- A. Woyciesjes
M. echinospora DSM 43816^{T}	DSM 1040	<- NRRL <- Schering-Plough Corp., JI-20 (*Micromonospora purpurea*)
M. endolithica DSM 44398^{T}	DSM 44398	<- P. Hirsch, Univ. Kiel; AA-459
M. haikouensis DSM 45626^{T}	DSM 45626	<- Q. Xie, Inst. of Tropical Bioscience & Biotech., Haikou City; 232617
M. halophytica DSM 43171^{T}	DSM 43171	<- KCC <- NRRL <- Schering AG
M. humi DSM 45647^{T}	DSM 45647	<- JCM; JCM 15292 <- S. Tanasupawat; P0402
M. inositola DSM 43819^{T}	DSM 43819	<- NRRL <- ATCC <- Kyowa Ferm. Ind. Co., Ltd., MK-41
M. inyonensis DSM 46123^{T}	DSM 46123	<- IMET <- M.A. Sveshnikova, INA (*Micromonospora inyoensis*)
M. krabiensis DSM 45344^{T}	DSM 45344	<- JCM/RIKEN <- S. Tanasupawat; MA-2
M. lupini Lupac 08	DSM 44870	<- M. E. Trujillo; Lupac 08
M. marina DSM 45555^{T}	DSM 45555	<- JCM <- S. Tanasupawat; JSM1-1
M. matsumotoense DSM 44100^{T}	DSM 44100	<- IFO <- K. Asano, Kyowa Hakko Co., 6393-C
M. mirobrigensis DSM 44830^{T}	DSM 44830	<- M. E. Trujillo, Universidad de Salamanca, Spain; WA201
M. narathiwatensis DSM 45248^{T}	DSM 45248	<- JCM/RIKEN; JCM 12394 <- C. Thawai; BTG4-1
M. nigra DSM 43818^{T}	DSM 43818	<- NRRL
M. olivasterospora DSM 43868^{T}	DSM 43868	<- ATCC <- Kyowa Ferm. Ind. Co., Ltd., MK-70
M. pallida DSM 43817^{T}	DSM 43817	<- NRRL
M. peucetia DSM 43363^{T}	DSM 43363	<- A. Grein, B-211 (*Micromonospora peucetieci*)
M. purpureochromogenes DSM 43821^{T}	DSM 43821	<- NRRL <- ATCC <- G. Luedemann <- IMRU
M. rhizosphaerae DSM 45431^{T}	DSM 45431	<- K. Hong, Inst. of Tropical Bioscience & Biotech., Haikou City; 211018 <- X. Xu
M. rifamycinica DSM 44983^{T}	DSM 44983	<- H. Huang, CGMCC
M. saelicesensis DSM 44871^{T}	DSM 44871	<- M. E. Trujillo; Lupac 09
M. sagamiensis DSM 43912^{T}	DSM 43912	<- JCM <- KCC <- NRRL <- Kyowa Hakko Co., MK-65
M. sediminicola DSM 45794^{T}	DSM 45794	<- C Suriyachadkun, BCC; BCC 45601 <- K. Supong
M. siamensis DSM 45097^{T}	DSM 45097	<- JCM <- C. Thawai; TT2-4
M. tulbaghiae DSM 45142^{T}	DSM 45142	<- P. R. Meyers, Univ. Cape Town, South Africa; TVU1 <- B. M. Kirby
M. viridifaciens DSM 43909^{T}	DSM 43909	<- JCM (*Micromonospora viridifaciens*) <- KCC <- KY 11078 <- ATCC <- Chas Pfizer & Co., FD 23988
M. yangpuensis DSM 45577^{T}	DSM 45577	<- Y. Huang, State Key Lab. Microbial Resources, Inst. Microbiol., Chinese Academy of Sciences, Beijing; FXJ6.011 <- Y. Fan
M. zamorensis DSM 45600^{T}	DSM 45600	<- M. E. Trujillo, Universidad de Salamanca, Spain; CR 38 <- L. Carro

Supplementary Table 9. Strategies used to generate the genomes of the *Micromonospora* strains.

	Study Name	Sequencing Centre	IMG Genome ID	GOLD Project ID	Assembly Method	DSMZ number	Bioproject	Sequencing Method
M. aurantiaca ATCC 27029^{T}	CSP_787436	JGI	648028042	Gp0001963	Newbler v. 2.3	DSM 43813	PRJNA37957	454, Illumina
M. aurantiaca L5	CSP_787436	JGI	649633069	Gp0006380	Velvet	-	PRJNA38291	454, Illumina
M. aurantiaca DSM 45487	ACTINO-1000	JGI	2619619011	Gp0112920	HGAP v. 2.3.0	DSM 45487	PRJNA303513	PacBio RS
M. auratinigra DSM 44815^{T}	ACTINO-1000	JGI	2617270871	Gp0112903	HGAP v. 2.3.0	DSM 44815	PRJNA303524	PacBio RS
M. carbonacea DSM 43168^{T}	GEBA(KMG-II)	JGI	2622736592	Gp0113037	ALLPATHS v. r46652	DSM 43168	PRJNA303620	Illumina HiSeq
M. chaiyaphumensis DSM 45246^{T}	GEBA(KMG-II)	JGI	2622736432	Gp0112486	ALLPATHS v. r46652	DSM 45246	PRJNA303414	Illumina HiSeq
M. chalcea DSM 43026^{T}	*M. chalcea*	NU	2675902971	Gp0146794	Spades	DSM 43026	PRJNA326336	Illumina HiSeq
M. chersina DSM 44151^{T}	ACTINO-1000	JGI	2623620611	Gp0112930	HGAP v. 2.3.0	DSM 44151	PRJNA303548	PacBio RS
M. chokoriensis DSM 45160^{T}	ACTINO-1000	JGI	2623620610	Gp0112921	HGAP v. 2.3.0	DSM 45160	PRJNA303512	PacBio RS
M. citrea DSM 43903^{T}	ACTINO-1000	JGI	2623620569	Gp0112904	HGAP v. 2.3.0	DSM 43903	PRJNA303523	PacBio RS
M. coriariae DSM 44875^{T}	ACTINO-1000	JGI	2623620612	Gp0112902	HGAP v. 2.3.0	DSM 44875	PRJNA303525	PacBio RS
M. coxensis DSM 45161^{T}	ACTINO-1000	JGI	2623620609	Gp0112922	HGAP v. 2.3.0	DSM 45161	PRJNA303511	PacBio RS
M. cremea DSM 45599^{T}	ACTINO-1000	JGI	2630968262	Gp0116630	HGAP v. 2.3.0	DSM 45599	PRJNA303682	PacBio RS
M. eburnea DSM 44814^{T}	ACTINO-1000	JGI	2622736604	Gp0112900	HGAP v. 2.3.0	DSM 44814	PRJNA303527	PacBio RS
M. echinaurantiaca DSM 43904^{T}	ACTINO-1000	JGI	2623620557	Gp0112906	HGAP v. 2.3.0	DSM 43904	PRJNA303522	PacBio RS
M. echinofusca DSM 43913^{T}	ACTINO-1000	JGI	2623620567	Gp0112907	HGAP v. 2.3.0	DSM 43913	PRJNA303521	PacBio RS
M. echinospora DSM 1040	R&D 2014	PB, JGI	2585427557	Gp0101212	HGAP v. 2.3.0	DSM 1040	PRJNA256905	PacBio RS
M. echinospora DSM 43816^{T}	ACTINO-1000	JGI	2622736613	Gp0112911	HGAP v. 2.3.0	DSM 43816	PRJNA303517	PacBio RS
M. endolithica DSM 44398^{T}	R&D 2014	PB, JGI	2585427558	Gp0101210	HGAP v. 2.3.0	DSM 44398	PRJNA256901	PacBio RS
M. haikouensis DSM 45626^{T}	GEBA(KMG-II)	JGI	2622736584	Gp0113041	ALLPATHS v. r46652	DSM 45626	PRJNA303616	Illumina HiSeq
M. halophytica DSM 43171^{T}	GEBA(KMG-II)	JGI	2622736531	Gp0113010	ALLPATHS v. r46652	DSM 43171	PRJNA303578	Illumina HiSeq
M. humi DSM 45647^{T}	GEBA(KMG-II)	JGI	2622736513	Gp0112487	ALLPATHS v. r46652	DSM 45647	PRJNA303413	Illumina HiSeq
M. inositola DSM 43819^{T}	ACTINO-1000	JGI	2623620396	Gp0112908	HGAP v. 2.3.0	DSM 43819	PRJNA303520	PacBio RS
M. inyonensis DSM 46123^{T}	ACTINO-1000	JGI	2636415978	Gp0116626	HGAP v. 2.3.0	DSM 46123	PRJNA303685	PacBio RS
M. krabiensis DSM 45344^{T}	ACTINO-1000	JGI	2617270872	Gp0112923	HGAP v. 2.3.0	DSM 45344	PRJNA303510	PacBio RS
M. lupini Lupac 08	*M. lupini* str. Lupac 08	Eurofins	2531839708	Gp0020554	Newbler	DSM 44870	PRJNA84455	454
M. marina DSM 45555^{T}	GEBA(KMG-II)	JGI	2622736534	Gp0112488	ALLPATHS v. r46652	DSM 45555	PRJNA303412	Illumina HiSeq
M. matsumotoense DSM 44100^{T}	GEBA(KMG-II)	JGI	2622736591	Gp0112489	ALLPATHS v. r46652	DSM 44100	PRJNA303502	Illumina HiSeq
M. mirobrigensis DSM 44830^{T}	GEBA(KMG-II)	JGI	2619619047	Gp0112994	ALLPATHS v. r46652	DSM 44830	PRJNA303592	Illumina HiSeq
M. narathiwatensis DSM 45248^{T}	ACTINO-1000	JGI	2617270834	Gp0112924	HGAP v. 2.3.0	DSM 45248	PRJNA303509	PacBio RS
M. nigra DSM 43818^{T}	ACTINO-1000	JGI	2619619644	Gp0112925	HGAP v. 2.3.0	DSM 43818	PRJNA303508	PacBio RS
M. olivasterospora DSM 43868^{T}	R&D 2014	PB, JGI	2585427559	Gp0101209	HGAP v. 2.3.0	DSM 43868	PRJNA256902	Illumina HiSeq
M. pallida DSM 43817^{T}	ACTINO-1000	JGI	2636415968	Gp0116625	HGAP v. 2.3.0	DSM 43817	PRJNA303686	PacBio RS
M. peucetia DSM 43363^{T}	ACTINO-1000	JGI	2622736606	Gp0112909	HGAP v. 2.3.0	DSM 43363	PRJNA303519	PacBio RS
M. purpureochromogenes DSM 43821^{T}	ACTINO-1000	JGI	2627854289	Gp0116627	HGAP v. 2.3.0	DSM 43821	PRJNA303684	PacBio RS
M. rhizosphaerae DSM 45431^{T}	ACTINO-1000	JGI	2622736626	Gp0112910	HGAP v. 2.3.0	DSM 45431	PRJNA303518	PacBio RS
M. rifamycinica DSM 44983^{T}	ACTINO-1000	JGI	2623620551	Gp0112901	HGAP v. 2.3.0	DSM 44983	PRJNA303526	PacBio RS
M. saelicesensis DSM 44871^{T}	GEBA(KMG-II)	JGI	2622736537	Gp0112995	ALLPATHS v. r46652	DSM 44871	PRJNA303591	Illumina HiSeq
M. sagamiensis DSM 43912^{T}	R&D 2014	PB, JGI	2585427560	Gp0101211	HGAP v. 2.3.0	DSM 43912	PRJNA256906	PacBio RS
M. sediminicola DSM 45794^{T}	ACTINO-1000	JGI	2622736535	Gp0112913	HGAP v. 2.3.0	DSM 45794	PRJNA303515	PacBio RS
M. siamensis DSM 45097^{T}	ACTINO-1000	JGI	2634166273	Gp0116631	HGAP v. 2.3.0	DSM 45097	PRJNA303681	PacBio RS
M. tulbaghiae DSM 45142^{T}	GEBA(KMG-II)	JGI	2622736612	Gp0113018	ALLPATHS v. r46652	DSM 45142	PRJNA303570	Illumina HiSeq
M. viridifaciens DSM 43909^{T}	ACTINO-1000	JGI	2636416073	Gp0116628	HGAP v. 2.3.0	DSM 43909	PRJNA303683	PacBio RS
M. yangpuensis DSM 45577^{T}	ACTINO-1000	JGI	2623620524	Gp0112926	HGAP v. 2.3.0	DSM 45577	PRJNA303507	PacBio RS
M. zamorensis DSM 45600^{T}	ACTINO-1000	JGI	2617270899	Gp0112914	HGAP v. 2.3.0	DSM 45600	PRJNA303514	PacBio RS

Illumina HiSeq 2500-1TB

GEBA: Genomic Encyclopedia of Bacteria and Archaea

ACTINO-1000: Exploiting the genomes of the Actinobacteria: plant growth promoters and producers of natural products and energy relevant enzymes united in a taxonomically unresolved phylum

JGI: DOE Joint Genome Institute (JGI); PB: Pacific Biosciences; NU: Northumbria University

Supplementary Table 10. Number of genes implicated in stress responses, DNA repair, central metabolism and environmental adaptation of the genomes of the *Micromonospora* strains.

Table in Supplementary tables file.

Supplementary reference list

Number	Reference number in manuscript
1	This study
2	240
3	101
4	241
5	102
6	109
7	60
8	54
9	242
10	104
11	243
12	103
13	111
14	68
15	62
16	97
17	106
18	66
19	244
20	98
21	69
22	213
23	108
24	245
25	112
26	114
27	95
28	59
29	56
30	44
31	105
32	70
33	113
34	215
35	110
